# Shiftwork, functional bowel symptoms, and the microbiome

**DOI:** 10.7717/peerj.11406

**Published:** 2021-05-11

**Authors:** Ann E. Rogers, Yi-Juan Hu, Ye Yue, Emily F. Wissel, Robert A. Petit III, Simone Jarrett, Jennifer Christie, Timothy D. Read

**Affiliations:** 1 Nell Hodgson Woodruff School of Nursing, Emory University, Atlanta, GA, United States of America; 2 Department of Biostatistics and Bioinformatics, Rollins School of Public Health, Emory University, Atlanta, GA, United States of America; 3 Investigational Clinical Microbiology Core, Emory University, Atlanta, GA, United States of America; 4 Einstein Medical Center Philadelphia, Philadelphia, PA, United States of America; 5 Division of Digestive Diseases, Emory School of Medicine, Emory University, Atlanta, GA, United States of America

**Keywords:** Shift work, Irritable bowel syndrome, Microbiome, Circadian rhythms

## Abstract

**Background:**

There are about 15 million Americans working full-time on evening, night, or rotating shifts. Between 48% and 81.9% of those working rotating or night shifts report abdominal pain, constipation, diarrhea and other symptoms of functional bowel disorders. The basis for this high prevalence of functional bowel disorders, including irritable bowel syndrome (IBS), among shift workers is unknown. Animal studies, however, suggest that circadian disruption, similar to that in shift workers, may contribute to the development of GI complaints among shift workers by altering the composition and normal diurnal rhythmicity of the resident intestinal microbes. Therefore, the present study was designed to determine if there were differences in (1) composition and diversity of the microbiome of night shift workers compared to day shift workers; and (2) the composition and diversity of the microbiome among shift workers experiencing functional bowel symptoms compared to shift workers who did not experience functional bowel symptoms.

**Methods:**

Fifty-one full time staff nurses who worked either 12-hour day or night shifts completed demographic information, and the Rome III IBS module. They also collected two samples of gut microbiota before the beginning and at the end of their last work shift on day 14, using validated field-tested methods consistent with the Human Microbiome Project. After DNA extraction, 16S rRNA sequencing and assignment to the genus level was completed, samples were then compared to determine if there were (1) differences in the diversity and profile of the microbiome by shift type; (2) if there were differences in the microbiome by time of day for collection; and (3) whether there were differences in the diversity and profile of the microbiome of nurses with IBS and those without IBS.

**Results:**

There were no differences in alpha or beta diversity of gut microbiota when specimens from day and night shift nurses were compared. There were however marginal differences in beta diversity when specimens collected at the beginning and end of the shifts were compared, with seven OTUs being differentially abundant when collected from day shift workers in the evening. There were also three OTUs to be differentially abundant in participants reporting IBS symptoms.

## Introduction

There are about 15 million Americans working full-time on evening, night, or rotating shifts, or other irregular employer-arranged schedules; 4.7% on evening shifts, 3.2% on night shifts, 3.1% on irregular schedules, and 2.5% on rotating shifts (United States Department of Labor Bureau of Labor Statistics, 2005). Night shift work is associated with increased mortality, higher risk of cardiovascular disease, cancer, diabetes, hypertension, chronic fatigue, sleep problems and higher body weight (Gu et al., 2015; Jia et al., 2013; Myers et al., 2015; Pan et al., 2011; Rajaratnam et al., 2011; Vyas et al., 2012). Night and rotating shift workers also report a higher prevalence of Irritable Bowel Syndrome (IBS), abdominal pain, constipation and diarrhea than do day shift workers (Caruso, Lusk & Gillespie, 2004; Knutsson & Boggild, 2010; Nojkov et al., 2010; Wells, Roth & Chande, 2012). In fact, between 48% and 82% of those working rotating or night shifts report abdominal pain, constipation, diarrhea and other symptoms of functional bowel disorders (Nojkov et al., 2010; Saberi & Moravveji, 2010).

The basis for this high prevalence of functional bowel disorders, including IBS, among shift workers is unknown. However, some studies suggest that inappropriate nutrition or irregularity in the timing of meals (Bilski, 2006; Lowen et al., 2010), and psychological disorders (Zhen, Ann & Yu, 2006) may contribute to the high prevalence of functional bowel symptoms among workers on rotating or night shifts.

Other studies strongly suggest that sleep deprivation or sleep disturbances are associated with the presence and severity of functional bowel symptoms reported by resident physicians and nurses (Jarrett et al., 2000; Saberi & Moravveji, 2010; Wells, Roth & Chande, 2012). Moreover, animal studies suggest that circadian disruption, similar to that in shift workers, contributes to the development of gastrointestinal (GI) complaints among shift workers by altering the composition and normal diurnal rhythmicity of the resident intestinal microbes (De Bacquer et al., 2009). Interestingly, gut microbiota community composition and diversity are malleable and sensitive to changes in diet and other environmental factors (Voigt et al., 2014), including activity and sleep patterns. For example, Thaiss and colleagues (Thaiss et al., 2014) “jet lagged” a group of mice by subjecting them to an 8-hour advance for three days before allowing them to revert to their usual schedule for three more days, then subjecting them to another 8 h advance for three days. Mice exposed to 4 weeks of this schedule lost their usual pattern of physical activity, and consumed food at irregular intervals. Significantly, this environmentally induced disruption of daily activity patterns (jet lag schedule) was associated with a loss of diurnal rhythmicity of microbiota composition in mice.

Thaiss and colleagues (2014) also found similar changes in human microbiota composition in two volunteers who flew from the US to Israel (an 8-10 h advance). Samples collected at baseline (one day pre-flight), during jet lag (one day after landing), and during recovery (2 weeks after landing) showed rapid changes in the composition of the microbiota. During jet lag (the first 24-hours after landing), there was a higher relative representation of Firmicutes, which reversed upon recovery from jet lag (2 weeks later). It is unknown it these changes would occur with the chronic circadian disruption experienced by night shift workers. Although some studies have demonstrated no differences in composition of the fecal microbiome when samples from lean and obese individuals were compared (Ley et al., 2006; Turnbaugh et al., 2009), other studies in humans have demonstrated that Firmicutes are associated with a higher propensity for obesity and metabolic disease (Finucane et al., 2014; Ley et al., 2006), conditions that are more common in night and rotating shift workers (De Bacquer et al., 2009; Suwazono et al., 2008).

Finally, multiple studies have linked reduced microbial diversity and richness in microbial communities to IBS symptoms. For example, Krogius-Kurikka and colleagues (Krogius-Kurikka et al., 2009) reported that fecal samples from patients with diarrhea-predominant IBS were enriched with Proteobacteria and Firmicutes but had reduced Actinobacteria and Bacteroidetes compared to healthy controls. Other studies (Bhattarai, Pedrogo & Kashyap, 2017; Salonen, de Vos & Palva, 2010) have shown an increase in the Firmicutes-to-Bacteriodetes-ratio, a decrease in some types of Firmicutes families (Lactobacilli, Faecalibacterium) and the Actinobacteria population (Bifidobacteria, Collinsella), and an increase in some Firmicutes families (Veillonella, Streptococci, and Ruminococcus spp.) and in Proteobacteria (Enterobacteriaceae spp.). In addition, low microbial richness, an absence of Methanobacteriales, and enrichment with Bacteroides enterotypes are associated with more severe IBS symptoms (Tap 2017). Not only is the composition of the fecal microbiota altered in patients with IBS, these imbalances in the microbial community or dysbiosis, occur more frequently in patients with IBS compared to healthy individuals. Reduced diversity was observed in nearly three-fourths of the IBS patients studied by Casén and colleagues (2015) compared to 16% in normal individuals (Collins, 2014; Jeffery et al., 2012).

While these studies suggest that shiftwork alters gut microbiota and that alterations in gut microbiota are common in patients with IBS, they do not demonstrate whether these alterations are associated with the somatic symptoms experienced by many rotating and night shift workers. Therefore, the present study is designed as the initial step in determining if there are differences in (1) composition and diversity of the microbiome of night shift workers compared to day shift workers; and (2) the composition and diversity of microbiome among night shift workers experiencing functional bowel symptoms (e.g., bloating, lower abdominal pain, constipation and diarrhea) compared to night shift workers not experiencing functional bowel symptoms.

## Materials & Methods

### Subjects

Participants in this study included 51 full-time staff nurses who worked 12-hour day or night shifts at a large university hospital. Registered nurses were eligible to participate if they were between the ages of 18 and 65 and did not report a history of inflammatory bowel disease (e.g., Crohn’s disease or ulcerative colitis) or other chronic disorder affecting the GI track (e.g., GI cancer). Those with recent antibiotic exposure were asked to delay their enrollment in the study for two weeks after their last dose of antibiotics.

As expected, the sample was predominantly female (96%), with a mean age of 32.9 ± 10.0 years and a range of 21–59 years. Just under half of the participants reported working straight day shifts (47%), with the remainder of the sample working either straight night shifts (51%) or rotating shifts (2%). For purposes of the analysis, the nurse who reported working rotating shifts was categorized as working night shift since she worked straight nights during the two-week data-gathering period. Although only three participants (6%) reported a prior diagnosis of IBS, a total 18 participants (35%) met criteria for the diagnosis of IBS using the Rome III criteria. Participant BMIs ranged from 18.2 to 39.5 with a mean BMI of 26.7 ±5.4. As illustrated in Table 1, there were no significant differences by shift type in terms of age, BMI, diagnosis of IBS or type of IBS.

**Table 1:** A Comparison of day and night shift participants.

	Day shift (*n* = 24)	Night shift (*n* = 27)	*P* value
Age (mean)	32.4	33.3	0.73^*^
BMI (mean)	27.1	26.3	0.60
BMI			
<20	1	3	0.41^**^
20–24.9	10	8	
25–29.9	5	10	
>30	8	6	
IBS (Rome III criteria)
No	17	16	0.56^**^
Yes	7	11	
IBS Symptoms			
IBS with diarrhea	1	2	1.0^**^
IBS with constipation	1	1	
IBS mixed type	5	7	
IBS un-subtyped	0	1	

**Notes.**

* Welsh two-sample *t*-test.

** Fishers exact test.

### Instruments

Data for this pilot study was obtained using a variety of subjective and objective measures. A Demographic Questionnaire and Brief Health History was used to collect information about participant age, and the usual shift worked. Participants were also asked to report any previous diagnosis of inflammatory bowel disorders or chronic diseases affecting the GI, and to list current medications and supplements used. The IBS module from the Rome III Questionnaire (Drossman et al., 2006) consists of 10 questions that ask subjects to rate the frequency of recurrent abdominal pain or discomfort, onset of pain associated with a change in frequency of stools, and the onset of pain associated with a change in the form of stools. This module is considered the gold standard for assessing functional bowel symptoms.

Samples of fecal microbiota were collected just before the beginning and just after the end participants’ work shift at the end of the two week data-collection period using validated, field-tested methods consistent with the (Human Microbiome Consortium, 2012). Four specimens (two each time) were collected using the Elution-swap system (Copan). The rectal swabs were stored in one mL of Amies transport medium (Copan) and immediately frozen and stored until DNA extraction. Prior to extraction, fecal material (200 mg) was suspended in 500-µl lysozyme (20 mg/ml in 20 mM Tris–HCl pH 8, 2 mM EDTA, 1.2% w/v Triton X-100) and incubated at 37 °C for 2 h using the QIAamp^®^ DNA Stool Mini Kit (Qiagen, Inc., Valencia, CA).

### Procedure

After obtaining approval from the Emory University’s IRB (MOD001-IRB00089064) and Emory Healthcare’s Nursing Research Council, emails describing the study were sent to all staff nurses. Those interested in participating were instructed to contact the research team to schedule an appointment to provide informed consent, review study procedures, and complete the demographic and Rome III questionnaire. After written informed consent was obtained, the participant’s work schedule was then reviewed to determine an appropriate date to collect samples of fecal microbiota at the beginning and end of the participant’s shift.

### Data analysis

### DNA extraction & 16S rRNA sequencing

DNA extraction and 16S sequencing was performed by Omega Bioservices (Norcross, GA, USA) using a standard protocol. DNA was isolated using Omega Biotek Mag-Bind^®^ Universal Pathogen DNA Kit. The V3-V4 region of the bacterial 16S rRNA gene sequences were amplified using the primer pair containing the gene-specific sequences and Illumina adapter overhang nucleotide sequences. The full length primer sequences are: 16S Amplicon PCR Forward Primer (5′-TCGTCGGCAGCGTCAGATGTGTATAAGAGACAGCC TACGGGNGGCWGCAG) and 16S Amplicon PCR Reverse Primer (5′-GTCTCGTGGGC TCGGAGATGTGTATAAGAGACAGGACTACHVGGGTATCTAATCC). For amplicon PCR, each 25 *μ*L of polymerase chain reaction (PCR) reaction contained 12.5 ng of sample DNA as input, 12.5 µL 2x KAPA HiFi HotStart ReadyMix Kapa Biosystems, Wilmington, MA) and 5 µL of 1 µM of each primer. PCR reactions were carried with an initial denaturation step performed at 95 °C for 3 min followed by 25 cycles of denaturation (95 °C, 30 s), annealing (55  °C, 30 s) and extension (72 °C, 30 sec), and a final elongation of 5 min at 72 °C. PCR product was cleaned up from the reaction mix with Mag-Bind RxnPure Plus magnetic beads (Omega Bio-tek, Norcross, GA). A second index PCR amplification, used to incorporate barcodes and sequencing adapters into the final PCR product, was performed in 25 µL reactions, using the same master mix conditions as described above. Cycling conditions were as follows: 95  °C for 3 minutes, followed by 8 cycles of 95  °C for 30′, 55 °C for 30″ and 72 °C for 30′. A final, 5 minutes’ elongation step was performed at 72 °C. The libraries were normalized with Mag-Bind^®^ EquiPure Library Normalization Kit ((Omega Bio-tek, Norcross, GA) then pooled. The pooled library ∼600 bases in size was checked using an Agilent 2200 TapeStation and sequenced (2 × 300 bp paired-end read setting) on the MiSeq (Illumina, San Diego, CA). Sequence data was submitted to the National Center for Bioinformatic Information Short Read Archive database: accession PRJNA687007.

#### Processing of sequence data and taxonomic assignment to Amplicon Sequence Variants (ASVs)

Data processing, including demultiplexing, QC filtering, contamination and sample mislabeling data checks, ASV representation, taxonomy assignment via a reference database (Caporaso et al., 2010; Wang et al., 2007), and phylogeny and diversity analysis (Lozupone et al., 2007; Lozupone, Hamady & Knight, 2006; Lozupone, 2005) was done using R packages dada2 and phyloseq (Callahan et al., 2016; McMurdie & Holmes, 2013). After filtering for low quality reads and filtering possible contaminants, we obtained a range of 12,455–119,605 reads per sample with a mean of 33,933 (SD = 10,412 and a median of 33, 452 (scripts used to generate this data have been included as supplemental files dada2-analysis 1, and dada2-analysis 2).

### Statistical analysis

All statistical analyses were conducted using R package 3.6.3. A *p*-value less than 0.05 was considered statistically significant.

We computed the Chao1 (measuring species richness) and Shannon (measuring both richness and evenness) alpha diversity indices for each sample, since loss of taxonomic diversity in general is an indicator of disease state in many ecological systems. We log-transform Chao1 to be at the same scale as Shannon. We also computed the Bray-Curtis (measuring dissimilarity/distance of each pair of samples based on relative abundance data) and Jaccard (based on presence-absence data) beta diversity distance metrics. We used Principal Coordinates Analysis (PCoA) to visualize clustering of the samples based on these distance metrics.

Given that this is a pilot study with 51 subjects, the primary focus was analyzing the microbiome profiles (in terms of alpha diversity, beta diversity, and individual genera at the relative abundance and presence-absence scales) for (a) differences between day and night shift workers, (b) differences between the beginning and the end of the shift (main effect of time) and whether the differences depend on the shift type (time and shift type interaction), and (c) differences between subjects with and without IBS symptoms. Since each subject is measured four times (two at the beginning of the shift and two at the end) for the microbiome, this study has a matched-set design. Care should be taken in analyzing such matched-set data because the samples from the same subjects tend to have positive correlations and the samples taken at the same time for the same subjects tend to have even stronger positive correlations. While the analyses in (a) and (c) consist of two-group comparisons “between” sets, the analyses in (b) consist of two-group comparison “within” sets for the main effect of time as well as two-group comparison of the change (from the beginning to the end of the shift) “between” sets. We have developed the Linear Decomposition Model (LDM) method and extended the existing PERMANOVA method that both can handle all of these analyses (Hu & Saggen, 2020; Zhu, Scotten & Hu, in press). While PERMANOVA performs comparison at the community level based on a beta-diversity distance matrix, the LDM performs the same community-level comparison based on the relative abundance or presence-absence data. In addition, the LDM performs comparison at individual-genus level (e.g., detecting individual genera that are differently “abundant” or “present” between groups) with false-discovery rate control at the nominal level 10%. We also use the LDM for comparing the alpha diversity by treating it as a single genus.

**Figure 1:**
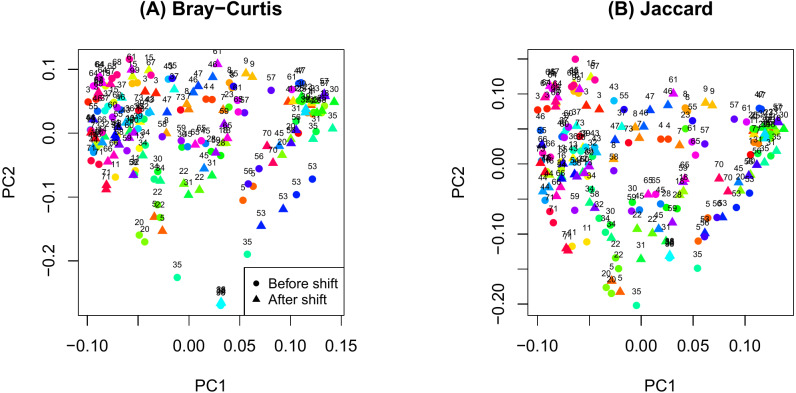
Principal components analysis plot by ID. (A) Bray-Curtis; (B) Jaccard.

## Results

The PCoA plot in Fig. 1 shows that samples obtained from the same participants tend to cluster together and samples from the same time on sample participants tend to cluster even more closely. This observation is consistent with the notion that microbiome samples are homogeneous within subjects and heterogenous across subjects, and thus confirms good quality of our data. Supplemental text (phyloseq analysis) contains plots showing the composition of the samples at taxonomic different levels

**Figure 2:**
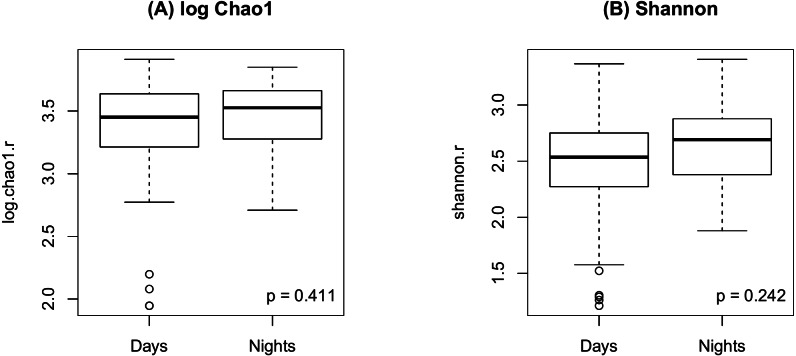
Alpha diversity by shift type. (A) log Chao1; (B) Shannon.

**Figure 3:**
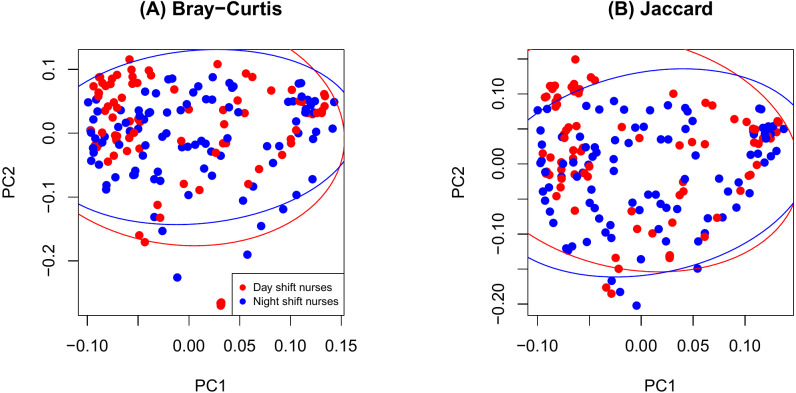
PCoA plot comparing beta diversity by shift type. (A) Bray-Curtis; (B) Jaccard.

As shown in Fig. 2, there were no significant differences in Chao1 (*p* = 0.411 by the LDM) and Shannon (*p* = 0.242) between day and night shift nurses. Nor were there differences in Bray-Curtis (*p* = 0.476 by PERMANOVA) and Jaccard distances (*p* = 0.625) by shift type (Fig. 3). Nor were there differences in relative abundance (*p* = 0.489 by the LDM) and presence-absence (*p* = 0.824) data across all genera.

**Figure 4:**
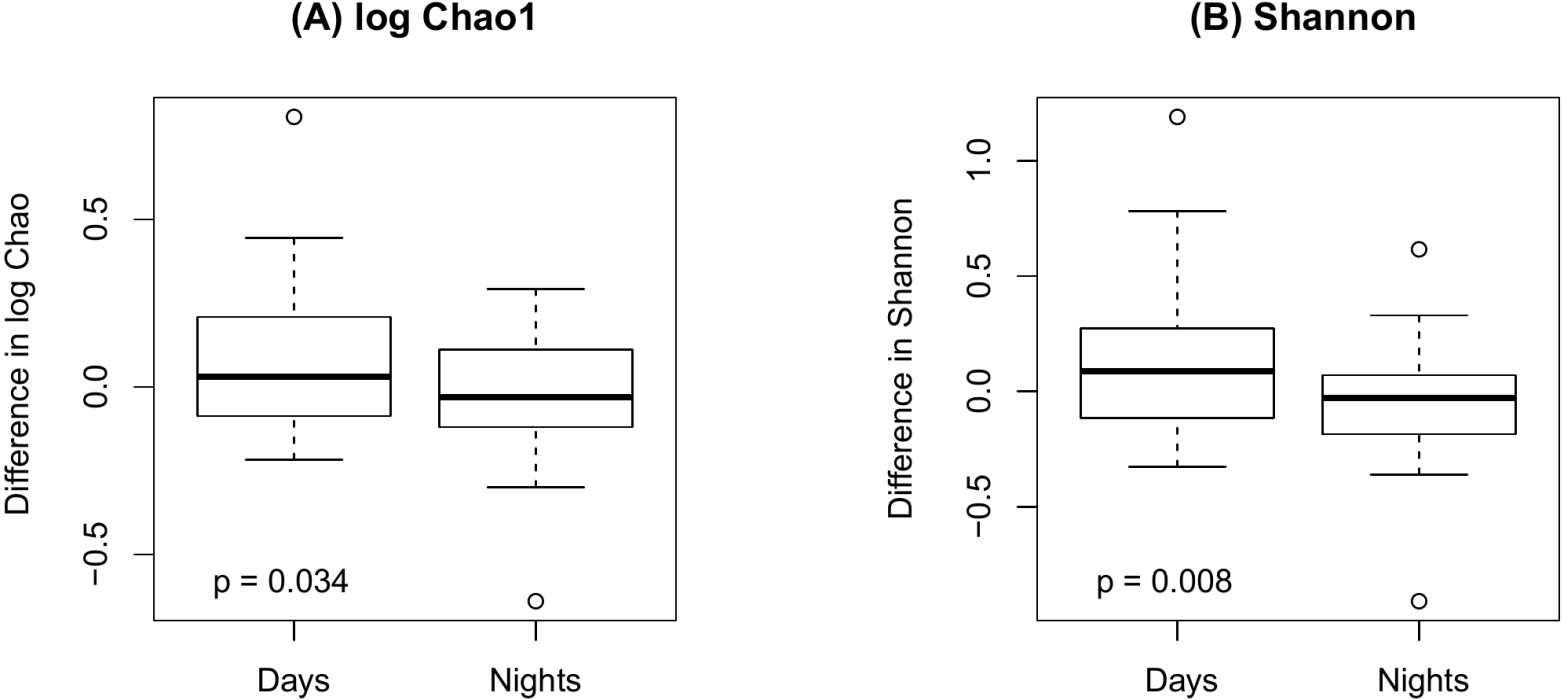
Changes in alpha diversity from the beginning to the end of the shift by shift type. (A) log Chao1; (B) Shannon.

Figure 4 shows an increase of the log Chao1 index from the beginning to the end of the shift for day-shift workers while a decrease for night-shift workers, and this difference of change is significant (interaction *p* = 0.034 by the LDM); so does the Shannon index (*p* = 0.008). However, the change among the pooled workers was not significantly different (main effect of time *p* = 0.473 for Chao1 and *p* = 0.236 for Shannon) possibly due to the cancellation of effects with opposite directions. In terms of beta diversity, the change of the beta diversity metric from the beginning to the end of the shift among the pooled workers was marginally significant or significant (main effect of time *p* = 0.056 by PERMANOVA for Bray-Curtis and 0.014 for Jaccard). Marginally significant and significant findings were also noted by the LDM (main effect of time *p* = 0.035 and 0.063 based on relative abundance and presence-absence data, respectively). However, there is not enough evidence to confirm that the change was significantly different between day shift and night shift workers possibly due to the small sample size, although there was suggestive evidence (interaction *p* = 0.192 and 0.118 by PERMANOVA based on Bray-Curtis and Jaccard, respectively; *p* = 0.320 and 0.134 by the LDM based on the relative abundance and presence-absence data). In addition, the LDM revealed seven ASVs to be differentially abundant between the beginning and the end of the shift; ASV_455(S5-A14a, more abundant at the beginning), ASV_2527(Ruminoccaceae_NK4A214_group, more abundant at the end), ASV_1304(Ruminococcus_1, more abundant at the end), ASV_221 (Mobiluncus, more abundant at the beginning), ASV_7(Campylobacter), ASV_130(Alistipes, more abundant at the end), and ASV_62(Agathobacter, more abundant at the end).

**Figure 5:**
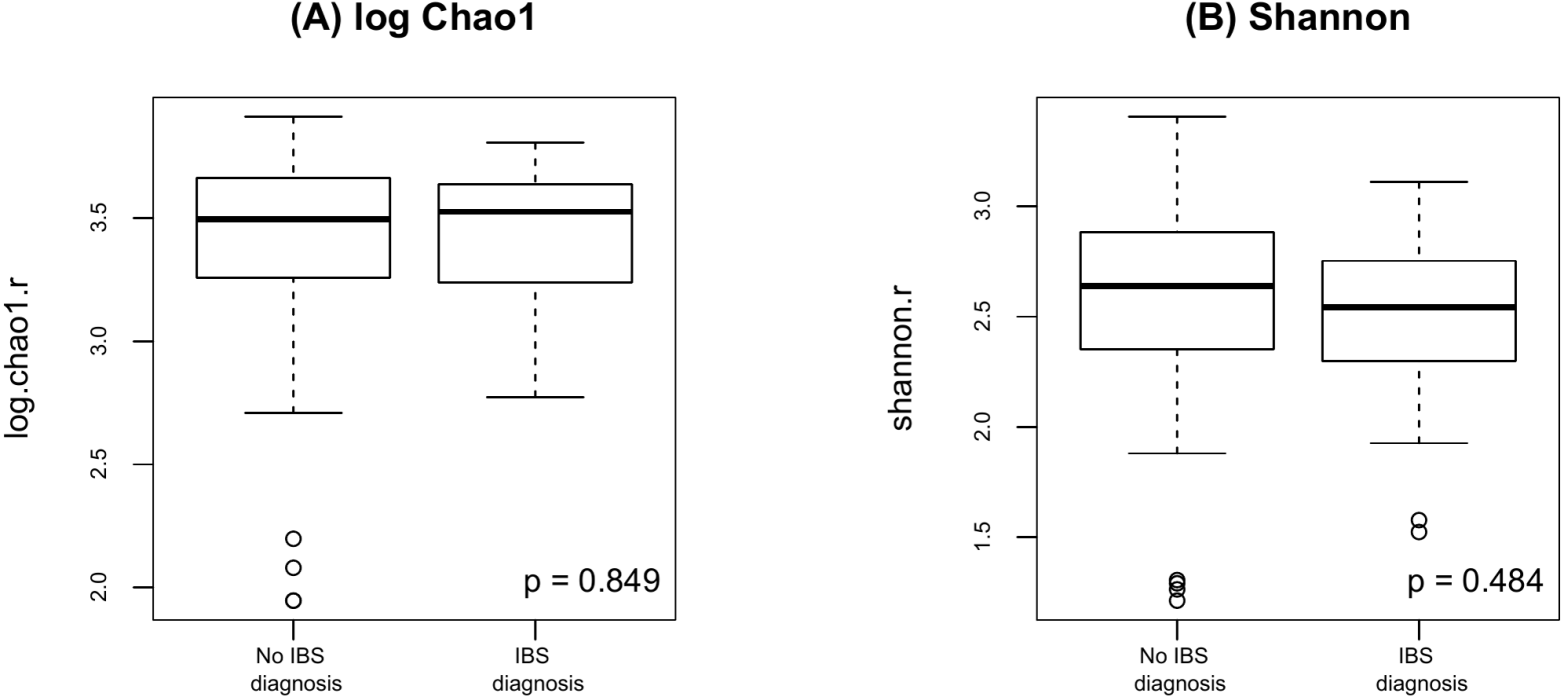
Alpha diversity by presence of absence of IBS. (A) log Chao1; (B) Shannon.

Finally, as shown in Fig. 5 there were no significant difference in alpha diversity when comparing participants without and with IBS symptoms (*p* = 0.849 for Chao 1, *p* = 0.484 for Shannon, by the LDM method). Although there were no differences in beta diversity (*p* = 0.206 for Bray-Curtis and *p* = 0.213 for Jaccard) by whether or not the participant had symptoms of IBS), there were some significant differences based on the LDM results. Specifically, three ASVs, ASV_1160 (Flavonifractor), ASV_1134 (Oscillibacter) and ASV_2379 (Ruminiclostridium_9), were detected to be differentially abundant. Three ASVs, ASV_1160 (Flavonifractor), ASV_2379 (Ruminiclostridium_9) and ASV_47 (Escherichia/Shigella), were detected to be significantly more likely to be present in participants reporting IBS symptoms.

## Discussion

The findings of this pilot study suggest that there are no differences in the richness and diversity of species when samples from nurses working day and night shifts were compared. There were however some statistically significant changes in both alpha and beta diversity metrics when specimens collected at the beginning and end of the shifts were compared and there was also some evidence that the changes were different for day shift and night shift workers, with increased alpha diversity noted at the end of the day shift and decreased alpha diversity noted at the end of the night shift. Seven ASVs were found to be differentially abundant between the beginning and end of the shifts for the entire sample. In addition, there were three ASVs to be differentially abundant in participants reporting IBS symptoms.

Studies comparing the effects of shift work on the gut microbiome are limited and somewhat contradictory. For example, a study of 10 male security guards who worked both day and night shifts found there were no significant differences in in alpha or beta diversity within and across-subject variation for both shifts (Mortas, Bilici & Karakan, 2020). In contrast, slight changes in microbial abundance and diversity were noted when 22 subjects, aged 20–35 years, delayed their sleep period for 2–4 h (Liu et al., 2020). Although there have been studies comparing circadian variation in the gut microbiota in mice (Thaiss et al., 2014) and another describing the results samples collected during multiple time points over several days by two subjects (Thaiss et al., 2014), our study is the first to compare the richness and diversity of gut microbiota collected from 51 human participants at two different time points in 24 h.

Even though there are numerous studies that have reported increased and/or decreased amounts of various gut bacteria among patients with IBS (Bhattarai, Pedrogo & Kashyap, 2017; Casén et al., 2015; Pittayanon et al., 2019; Salonen, de Vos & Palva, 2010; Tap et al., 2017), a recent systematic review found only nine studies that discussed differences in alpha-diversity in patients with IBS compared to normal controls (Pittayanon et al., 2019). Slightly over half of the studies (55.6%) reported a significant decrease in the richness and diversity in patients with IBS (Carroll et al., 2011; Carroll et al., 2012; Liu et al., 2016; Rangel et al., 2015), whereas the remaining four studies (Carroll et al., 2012; Durban et al., 2012; Rigsbee et al., 2012; Tap et al., 2017), like our current study, revealed no differences in alpha-diversity compared to healthy controls. A more elaborate study (Pozuelo et al., 2015) comparing fecal and mucosa-associated microbiome samples obtained from the sigmoid colon during colonoscopy, not only found no differences in alpha diversity when the two types of samples were compared, but that there were no differences in richness and diversity when samples obtained from healthy controls and patients with IBS or IBS subtypes were compared.

Like other studies comparing patients with IBS symptoms to healthy controls, our pilot study found increased Firmicutes (Chong et al., 2019), specifically Flavonifractor, Oscillibacter, and Ruminiclostudium among participants with IBS (Casén et al., 2015). The increased abundance of E. coli/ Shigella possibly reflects the suspected relationship between Shigellosis and IBS (Youn et al., 2016). In contrast, Pozuelo et al. (2015) reported that Methanobacteriaceae, Ruminococcaceae, Erysipleotrichacea, and one unknown Clostridiales family were all found to be decreased in patients with IBS-M (IBS-mixed type) and IBS-D (IBS-diarrhea), but not in patients with IBS-C (IBS-constipation).

IBS is estimated to have a world-wide prevalence of 10–15% (Canavan, West & Card, 2014; Sperber et al., 2017). Prevalence rates of IBS are typically higher among nurses, with rates ranging from 17.4% in China (Liu et al., 2014) to 45.2% in Nigeria (Akere & Akande, 2014). The prevalence rate of IBS among study participants was 35%, quite similar to the prevalence rate reported among nurses at the University of Michigan Medical Center (36.6%) (Nojkov et al., 2010). However, unlike the study of hospital staff nurses (Nojkov et al., 2010), and other studies of shift workers (Kim et al., 2013), there were no differences in the prevalence of IBS symptoms among day and night shift nurses in our study.

This study is limited by a number of factors. First, our study population consisted of a convenience sample of nurses that might not be representative of the larger nursing workforce or the larger population of shift workers. The overall participation rate was relatively low, which raises concerns about how representative the participants were of the total population of nurses who were invited to participate. Additionally, given the focus of the study, nurses who experienced IBS symptoms may have been more likely to participate than those who did not experience IBS symptoms. Finally, the severity of IBS symptoms and quality of life was not assessed, two factors which may have been impacted by gut microbiome diversity and richness.

## Conclusions

There were no statistically significant differences in the richness and diversity when samples of the gut microbiome from nurses working day and night shifts were compared. However, when specimens collected at the beginning and ends of the shifts were compared, there were some statistically significant or marginally differences in alpha and beta diversity. Three ASVs were more common in participants reporting IBS symptoms.

##  Supplemental Information

10.7717/peerj.11406/supp-1 Supplemental Information 1 RMarkdown document describing commands for phyloseq-based analysis of the taxomony table derived from dada2 processing

10.7717/peerj.11406/supp-2 Supplemental Information 2 RMarkdown document describing commands used for FASTQ quality filtering and dada2 analysis

10.7717/peerj.11406/supp-3 Supplemental Information 3 Demographic data raw data

## Additional Information and Declarations

### Data Availability

The following information was supplied regarding data availability: Sequence data are available at the NBI SRA database: PRJNA687007. The PDRs of R scripts used for analysis of 16S microbiome data is available at figshare: Read, Timothy; Wissel, Emily; Petit, Robert (2021): Emory SLEEP 16S scripts. figshare. Figure. https://doi.org/10.6084/m9.figshare.14390363.v1

https://www.ncbi.nlm.nih.gov/bioproject/PRJNA687007.
