# Supplementary material for: Shiftwork, functional bowel symptoms, and the microbiome
Source: PeerJ. 2021 May 11;9:e11406. doi: 10.7717/peerj.11406 (PMC8121053; doi:10.7717/peerj.11406)
Supplement: Supplemental Information 1 [file peerj-09-11406-s001.pdf]

phyloseq-analysis

Please note that this documented was created from this bioconductor document (https://bioconductor.org/help/course-materials/2017/BioC2017/Day1/Workshops/Microbiome/MicrobiomeWorkflowII.html#agglomerate_taxa). Much of the text in this document is taken from this tutorial and editted to fit this specific project.

```
#source('http://bioconductor.org/biocLite.R')
#biocLite('phyloseq')

library(ggplot2)
library(dplyr)
library(phyloseq)
library(Biostrings)
library(readxl)
library(readr)
library(metagMisc)
library(knitr)
library(lemon)
PROJECT_DIR = "/data1/home/ewissel/16S-sleep"
setwd(PROJECT_DIR)
theme_set(theme_light())
knit_print.data.frame <- lemon_print

# install packages if not already installed for preprocessing section: uncommen
t if you need to download

#.cran_packages <- c( "shiny","miniUI", "caret", "pls", "e1071", "ggplot2", "ra
ndomForest", "dplyr", "ggrepel", "nlme", "devtools",
 #                  "reshape2", "PMA", "structSSI", "ade4",
#                   "ggnetwork", "intergraph", "scales")
#.github_packages <- c("jfukuyama/phyloseqGraphTest")
#.bioc_packages <- c("genefilter", "impute")
# Install CRAN packages (if not already installed)
#.inst <- .cran_packages %in% installed.packages()
#if (any(!.inst)){
#  install.packages(.cran_packages[!.inst],repos = "http://cran.rstudio.com/")
#}
#.inst <- .github_packages %in% installed.packages()
#if (any(!.inst)){
#  devtools::install_github(.github_packages[!.inst])
#}

#.inst <- .bioc_packages %in% installed.packages()
#if(any(!.inst)){
#  source("http://bioconductor.org/biocLite.R")
#  biocLite(.bioc_packages[!.inst])
#}
```

Read in Files and Set up for phyloseq

We need to read in the files and format everything for the phyloseq package. We need to ensure each file contains the same sample IDs. Sample IDs

```
meta_dat <- read_excel("Sample_metadata_table AER adds.xlsx")
```

```
## New names:
## * `` -> ...12
```

```
asvs_taxa <- read_tsv("results/ASVs-decontam-taxonomy.tsv")  #taxonomy table
```

```
## Warning: Missing column names filled in: 'X1' [1]
```

```
## Parsed with column specification:
## cols(
##   X1 = col_character(),
##   Kingdom = col_character(),
##   Phylum = col_character(),
##   Class = col_character(),
##   Order = col_character(),
##   Family = col_character(),
##   Genus = col_character(),
##   Species = col_character()
## )
```

```
asvs_count <- read_tsv("results/ASVs-decontam-counts.tsv") # this is the asv/ot
u matrix
```

```
## Warning: Missing column names filled in: 'X1' [1]
```

```
## Parsed with column specification:
## cols(
##   .default = col_double(),
##   X1 = col_character()
## )
```

```
## See spec(...) for full column specifications.
```

```
# sample_dat for phyloseq
as.data.frame(meta_dat) -> meta_dat
length( unique(meta_dat$Sample) ) # 186
```

```
## [1] 186
```

```
ncol(asvs_count)                  # 188
```

```
## [1] 188
```

```
# find what sample is different between the two and remove it
met_samps <- unique(meta_dat$Sample)
asv_samps <- names(asvs_count)

table( met_samps %in% asv_samps) # one file in meta data not in ASVs.
```

```
##
## FALSE  TRUE
##     1   185
```

```
table( asv_samps %in%  met_samps) # three files in asvs not in meta data.
```

```
##
## FALSE  TRUE
##     3   185
```

```
# this tells us we should have 185 samples total included in the analysis.

# if I remove the one false file from meta_samps, the following should work
met_samps<- met_samps %>% subset(met_samps %in% asv_samps)
length(met_samps) # this is now correct
```

```
## [1] 185
```

```
#filter otu table
asv_x1 <- asvs_count$X1 #need this for matrix below
asvs_count <- asvs_count[,met_samps] # only take columns whose name is in short
ened list
meta_dat <- subset(meta_dat, Sample %in% met_samps)
meta_dat <- meta_dat %>%
  mutate(IBS_type = ifelse(IBS_type==NA, "none", IBS_type)) #need this for pca
plot to work below
```

Now that the data has been read in, we need to create the phyloseq object.

```
#need to count/otu as a matrix from dataframe
only_num <- asvs_count
only_name <- asv_x1
only_name <- as.list(only_name)
mat <- as.matrix(only_num)
rownames(mat) <- only_name

## taxa table also need to be a matrix
asv_taxa <- asvs_taxa %>% select(-X1)
tname <- asvs_taxa%>% select(X1)
tax_mat <- as.matrix(asv_taxa)
rownames(tax_mat) <- as.list( tname$X1)

#metadata
met_dat <- sample_data(meta_dat)
sample_names(met_dat) <- met_samps
#tax table = asvs_taxa
#otu matrix = asvs_coutns
OTU = otu_table(mat, taxa_are_rows = TRUE)
TAX = tax_table(tax_mat)

physeq = phyloseq(OTU, TAX, met_dat)
rank_names(physeq)
```

```
## [1] "Kingdom" "Phylum"  "Class"   "Order"   "Family"  "Genus"   "Species"
```

```
# Create table, number of features for each phyla
table(tax_table(physeq)[, "Phylum"], exclude = NULL)
```

```
##
##      Actinobacteria       Bacteroidetes         Chloroflexi
##                1584                4901                   1
##       Cyanobacteria Epsilonbacteraeota          Euglenozoa
##                  20                 244                   1
##          Firmicutes        Fusobacteria Kiritimatiellaeota
##                9004                 466                   4
##       Lentisphaerae    Patescibacteria      Planctomycetes
##                  20                  24                   2
##      Proteobacteria             Retaria        Spirochaetes
##                 690                   1                  16
##       Synergistetes        Tenericutes     Verrucomicrobia
##                  45                  74                 130
##                <NA>
##                 231
```

Here we see all the phyla and the number of features observed. First, notice that in this case, 231 features were annotated with a Phylum of NA. These features are probably artifacts in a dataset like this, and should likely be removed. Also, if any of the Phyla have only one feature, this is likely noise and should be removed.

```
physeq <- subset_taxa(physeq, !is.na(Phylum) & !Phylum %in% c("", "uncharacteri
zed"))
#remove columns . taxa for which every observation is NA
physeq <- phyloseq_rm_na_tax(physeq)
# add tree
library("ape")
```

```
##
## Attaching package: 'ape'
```

```
## The following object is masked from 'package:Biostrings':
##
##     complement
```

```
my_tree = rtree(ntaxa(physeq), rooted=TRUE, tip.label=taxa_names(physeq))
# merge tree with physeq object
phy_tree(physeq) <- my_tree
```

A useful next step is to explore feature prevalence in the dataset, which we will define here as the number of samples in which a taxon appears at least once.

```
# Compute prevalence of each feature, store as data.frame
prevdf = apply(X = otu_table(physeq),
               MARGIN = ifelse(taxa_are_rows(physeq), yes = 1, no = 2),
               FUN = function(x){sum(x > 0)})
# Add taxonomy and total read counts to this data.frame
prevdf = data.frame(Prevalence = prevdf,
                    TotalAbundance = taxa_sums(physeq),
                    tax_table(physeq))
# examine if any phyla only have low abundance features
## 1 is total prevalence, 2 is average prevalence
plyr::ddply(prevdf, "Phylum", function(df1){cbind(mean(df1$Prevalence),sum(df
1$Prevalence))})
```

Phylum	1	2
Actinobacteria	3.669192	5812
Bacteroidetes	9.511324	46615

Phylum	1	2
Chloroflexi	1.000000	1
Cyanobacteria	1.050000	21
Epsilonbacteraeota	19.069672	4653
Euglenozoa	1.000000	1
Firmicutes	5.384496	48482
Fusobacteria	5.871245	2736
Kiritimatiellaeota	1.000000	4
Lentisphaerae	1.000000	20
Patescibacteria	1.166667	28
Planctomycetes	1.000000	2
Proteobacteria	4.921739	3396
Retaria	1.000000	1
Spirochaetes	1.750000	28
Synergistetes	1.311111	59
Tenericutes	1.270270	94
Verrucomicrobia	5.492308	714

For the table, if any of the columns contain the same value, this indicates that these phyla occur in low abundance and may be noise. It could be useful to check each feature manually to determine if it's real or noise. In our case, Retaria (https://en.wikipedia.org/wiki/Rhizaria) and Chloroflexi (https://en.wikipedia.org/wiki/Chloroflexi_(phylum)) appear to be artifacts and should be filtered out as they are in low abundance and wouldn't be expected to be in the gut. While Planctomycetes looks moderately suspicious, it is not surprising to see it in the gut so we will leave it.

```
# Define phyla to filter
filterPhyla = c("Chloroflexi", "Retarias", "Euglenozoa") #any of the above that
were
# Filter entries with unidentified Phylum.
physeq = subset_taxa(physeq, !Phylum %in% filterPhyla)
physeq
```

```
## phyloseq-class experiment-level object
## otu_table()   OTU Table:         [ 17225 taxa and 185 samples ]
## sample_data() Sample Data:       [ 185 samples by 12 sample variables ]
## tax_table()   Taxonomy Table:    [ 17225 taxa by 7 taxonomic ranks ]
## phy_tree()    Phylogenetic Tree: [ 17225 tips and 17224 internal nodes ]
```

Prevalence Filtering

Whereas the above filtering relied on a priori knowledge, this filtering relies only on what is in the dataset. We will first examine the relationship between prevalence and total read count per feature, as this may reveal outliers to be removed. This aspect depends quite a lot on the experimental design and goals of the downstream inference, so keep these in mind. It may even be the case that different types of downstream inference require different choices here. There is no reason to expect ahead of time that a single filtering workflow is appropriate for all analyses.

```
# Subset to the remaining phyla
prevdf1 = subset(prevdf, Phylum %in% get_taxa_unique(physeq, "Phylum"))

#make a pretty palette
library(pals)
```

```
##
## Attaching package: 'pals'
```

```
## The following object is masked from 'package:Biostrings':
##
##     alphabet
```

```
ggplot(prevdf1, aes(TotalAbundance, Prevalence / nsamples(physeq),color=Phylu
m)) +
  # Include a guess for parameter
  geom_hline(yintercept = 0.03, alpha = 0.5, linetype = 2) +
  geom_point(size = 2, alpha = 0.7) +
  scale_x_log10() +
  xlab("Total Abundance") +
  ylab("Prevalence [Frac. Samples]") +
  facet_wrap(~Phylum) +
  theme(legend.position="none")+
  scale_color_manual(values=as.vector(stepped2(18)))
```

```
## Warning: Transformation introduced infinite values in continuous x-axis
```

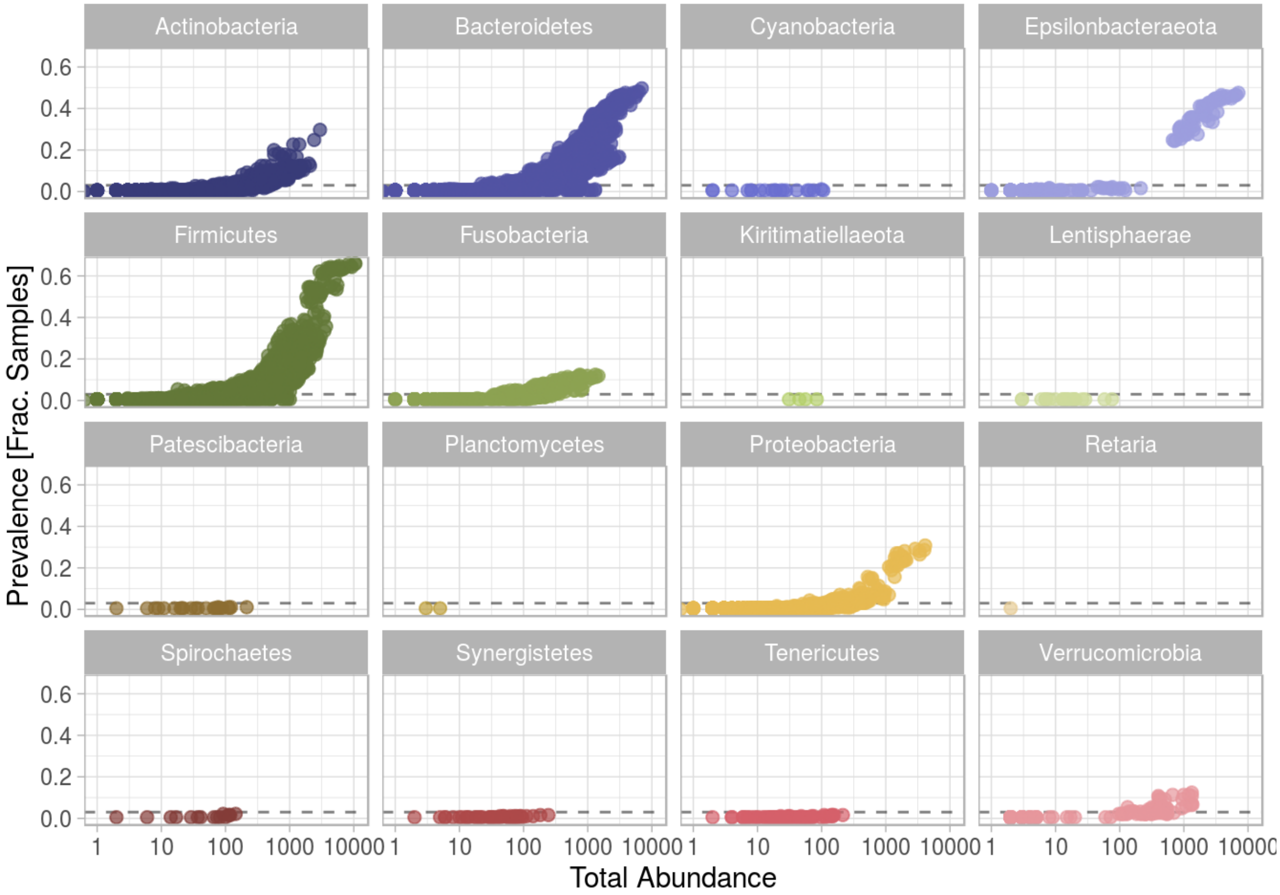

Each point in this figure is a feature. We will use these graphs to select what minimum prevalence should be required for our filtering. A natural break may present itself, but it may not. Be aware that this choice could produce introduction bias into downstream analysis of association of differential abundance if not careful. The dashed line is 0.03, indicating the filtering that will occur if a 3% prevelance threshold is used.

This graph can be used to test how good we think all the filtering has been up to this point. We see high levels of Bacteroidetes, Firmicutes, Fusobacteria,and Proteobacteria, which is what we would expect for a 16S gut microbiome sample. We also see high levels of Esilonbacteraeota - note that this is a new phylum of bacteria (https://www-ncbi-nlm-nih-gov.proxy.library.emory.edu/pmc/articles/PMC5401914/), previously a class in Proteobaceria, that has been documented in high levels of the gut microbiome, so this is as expected.

```
# Define prevalence threshold as 3% of total samples
prevalenceThreshold = 0.03 * nsamples(physeq)
prevalenceThreshold
```

```
## [1] 5.55
```

```
# Execute prevalence filter, using `prune_taxa()` function
keepTaxa = rownames(prevdf1)[(prevdf1$Prevalence >= prevalenceThreshold)]
ps2 = prune_taxa(keepTaxa, physeq)
```

Agglomerate Taxa

Here we will agglomerate the data features corresponding to closely related taxa. Ideally this will reduce functional redundancy in the dataset. While not necessarily the most useful or functionally-accurate criteria for grouping microbial features (sometimes far from accurate), taxonomic agglomeration has the advantage of being much easier to define ahead of time. This is because taxonomies are usually defined with a comparatively simple tree-like graph structure that has a fixed number of internal nodes, called “ranks”. This structure is simple enough for the phyloseq package to represent taxonomies as table of taxonomy labels. Taxonomic agglomeration groups all the “leaves” in the hierarchy that descend from the user-prescribed agglomerating rank, this is sometimes called ‘glomming’.

The following should how to combine all features that descend from the same genus. As this is 16S data, we can’t reliable go deeper than the genus level.

```
# how many genera would be present after filtering?
length(get_taxa_unique(ps2, taxonomic.rank = "Genus")) #94
```

```
## [1] 94
```

```
ps3 = tax_glom(ps2, "Genus", NArm = TRUE)
```

This is a taxonomy-free alternative to combine features of closely-related taxa. Below, the user specifies a tree height corresponding to phylogenetic distance between features that should define their ranking. This is similar to “OTU clustering” except that OTU clustering algorithms often do not have the same (or any) evolutionary definition.

```
h1 = 0.4
ps4 = tip_glom(ps2, h = h1)

multiPlotTitleTextSize = 15
p2tree = plot_tree(ps2, method = "treeonly",
                   ladderize = "left",
                   title = "Before Agglomeration") +
  theme(plot.title = element_text(size = multiPlotTitleTextSize))
p3tree = plot_tree(ps3, method = "treeonly",
                   ladderize = "left", title = "By Genus") +
  theme(plot.title = element_text(size = multiPlotTitleTextSize))
p4tree = plot_tree(ps4, method = "treeonly",
                   ladderize = "left", title = "By Height") +
  theme(plot.title = element_text(size = multiPlotTitleTextSize))

# group plots together
#using gridExtra:grid.arrange
library(gridExtra)
```

```
##
## Attaching package: 'gridExtra'
```

```
## The following object is masked from 'package:BiocGenerics':
##
##     combine
```

```
## The following object is masked from 'package:dplyr':
##
##     combine
```

```
grid.arrange(nrow = 1, p2tree, p3tree, p4tree)
```

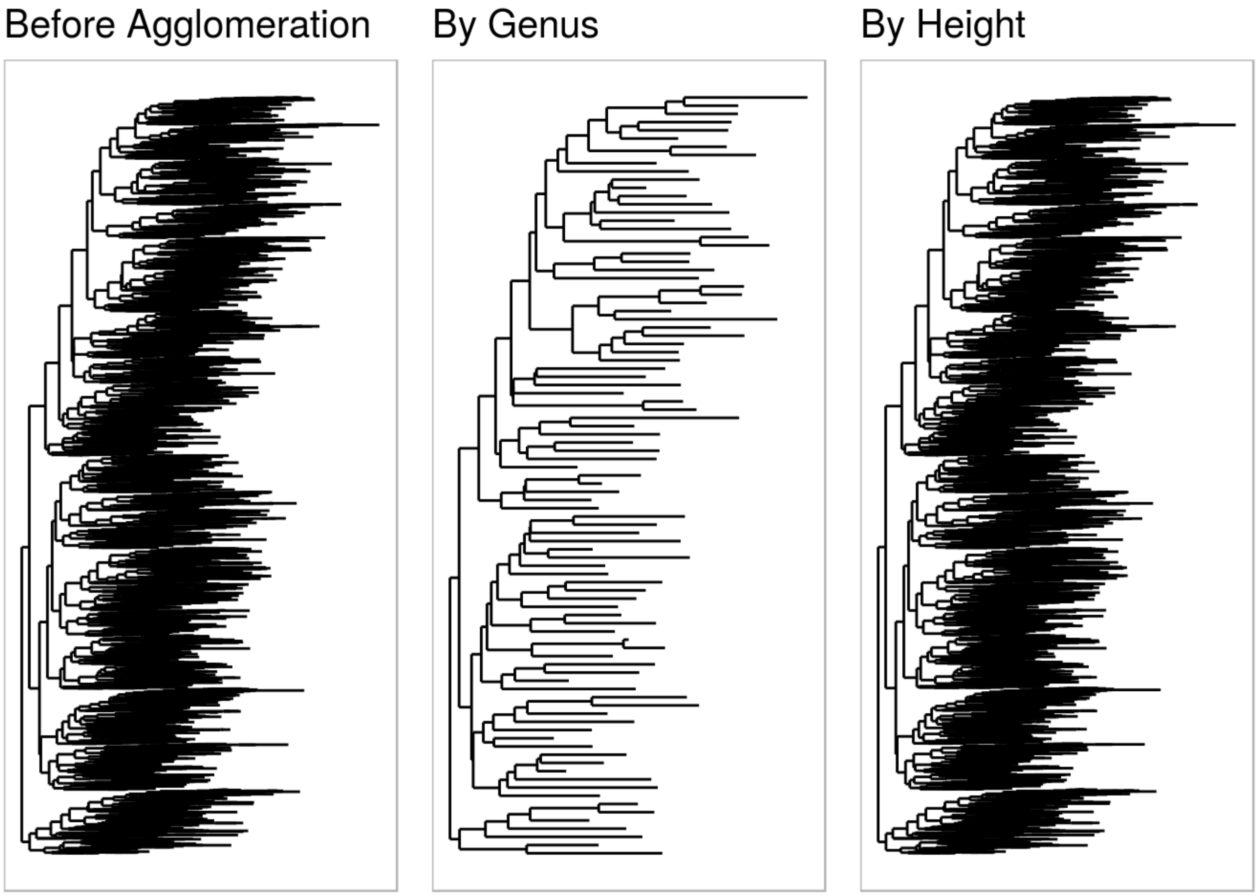

On the left is the original tree, in the middle is the taxonomic agglomeration at Genus level, and on the right is the phylogenetic agglomeration at a fixed distance of .

Abundance Value Transformation

It is often required to transform the data to account for differences in library size, variance, etc. We will use `phyloseq::transform_sample_counts()` to do this. The first argument to this function is the phyloseq object you want to transform, and the second argument is an R function that defines the transformation. The R function is applied sample-wise, expecting that the first unnamed argument is a vector of taxa counts in the same order as the phyloseq object.

This example begins by defining a custom plot function, plot_abundance(), that uses phyloseq's function to define a relative abundance graphic. We will use this to compare more easily differences in scale and distribution of the abundance values in our phyloseq object before and after transformation.

The transformation in this case converts the counts from each sample into their frequencies, often referred to as proportions or relative abundances. This function is so simple that it is easiest to define it within the function call to `transform_sample_counts()` .

```
plot_abundance = function(physeq,title = "",
                          Facet = "Order", Color = "Phylum"){
  # Arbitrary subset, based on Phylum, for plotting
  p1f = subset_taxa(physeq, Phylum %in% c("Firmicutes"))
  mphyseq = psmelt(p1f)
  mphyseq <- subset(mphyseq, Abundance > 0)
  ggplot(data = mphyseq, mapping = aes_string(x = "Time_collected",y = "Abundan
ce",
                              color = Color, fill = Color)) +
    geom_violin(fill = NA) +
    geom_point(size = 1, alpha = 0.3,
               position = position_jitter(width = 0.3)) +
    facet_wrap(facets = Facet) + scale_y_log10()+
    theme(legend.position="none")
}
# Transform to relative abundance. Save as new object.
ps3ra = transform_sample_counts(ps3, function(x){x / sum(x)})

plotBefore = plot_abundance(ps3,"")
```

```
## Warning in psmelt(p1f): The sample variables:
## Sample
##  have been renamed to:
## sample_Sample
## to avoid conflicts with special phyloseq plot attribute names.
```

```
plotAfter = plot_abundance(ps3ra,"")
```

```
## Warning in psmelt(p1f): The sample variables:
## Sample
##  have been renamed to:
## sample_Sample
## to avoid conflicts with special phyloseq plot attribute names.
```

```
# Combine each plot into one graphic.
grid.arrange(nrow = 2,  plotBefore, plotAfter)
```

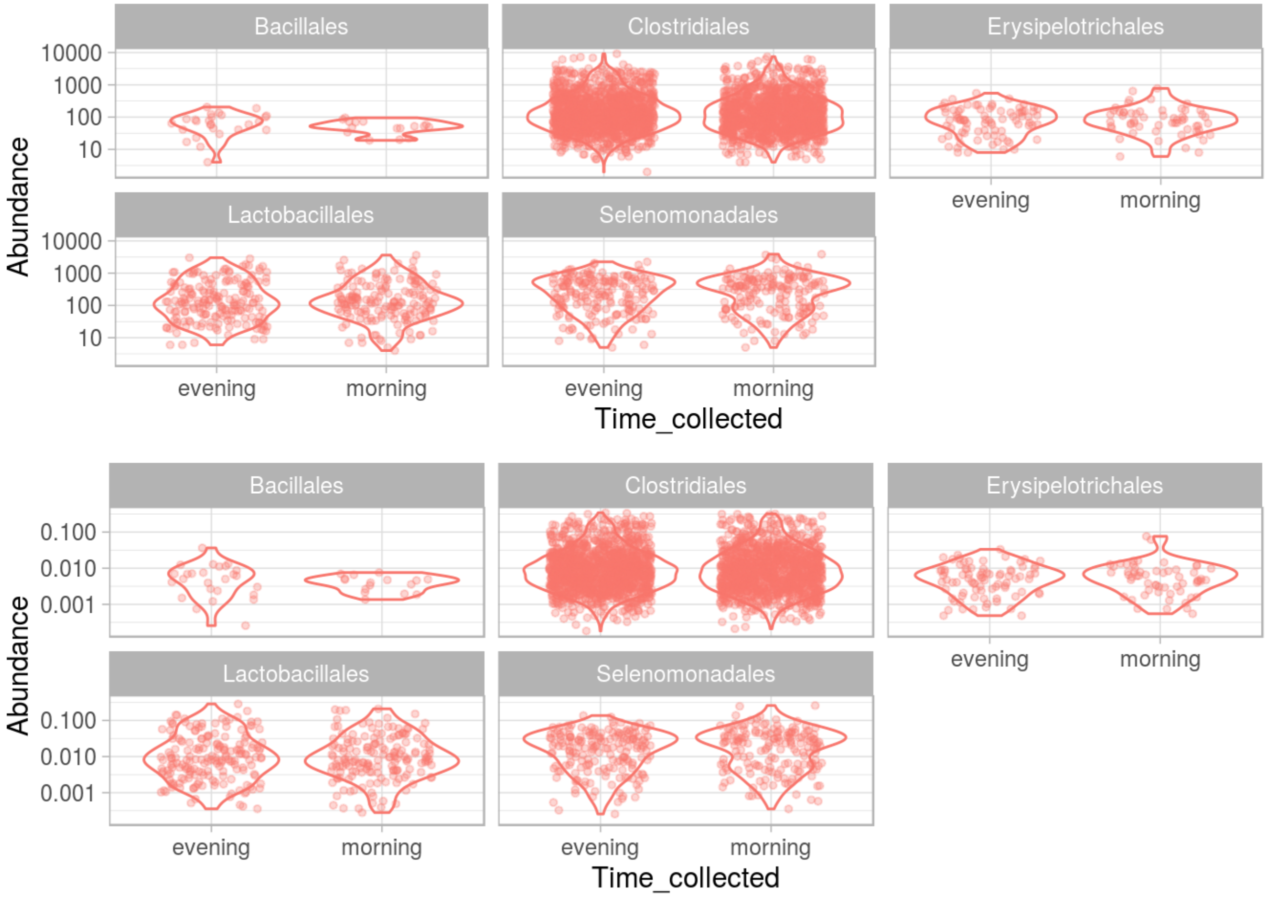

This figure shows the comparison of original abundances (top panel) and the relative abundances (lower) of the bacterial Orders.

Subset by Taxonomy

Notice if any of the Orders in the previous plot appear bimodal in abundance. We can check for a taxonomic explanation of any odd abundance patterns. Ours looks pretty evenly distributed, but let's examine Selenomonadales as an example.

```
psOrd = subset_taxa(ps3ra, Order == "Selenomonadales") #Selenomonadales and Lac
tobacillales
plot_abundance(psOrd, Facet = "Genus", Color = NULL)
```

```
## Warning in psmelt(p1f): The sample variables:
## Sample
##  have been renamed to:
## sample_Sample
## to avoid conflicts with special phyloseq plot attribute names.
```

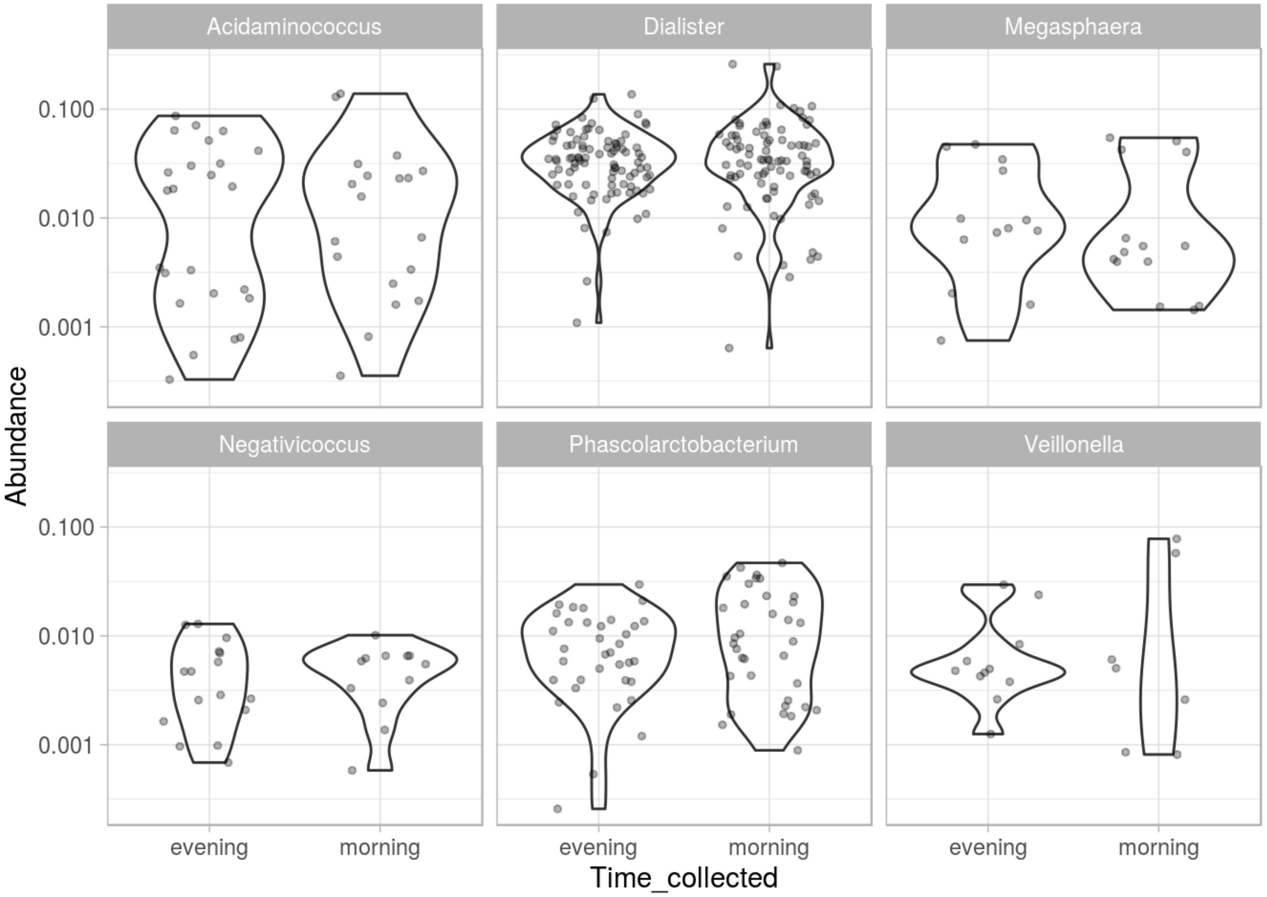


```
psOrd2 = subset_taxa(ps3ra, Order == "Lactobacillales") #Selenomonadales and La
ctobacillales
plot_abundance(psOrd2, Facet = "Genus", Color = NULL)
```

```
## Warning in psmelt(p1f): The sample variables:
## Sample
##  have been renamed to:
## sample_Sample
## to avoid conflicts with special phyloseq plot attribute names.
```

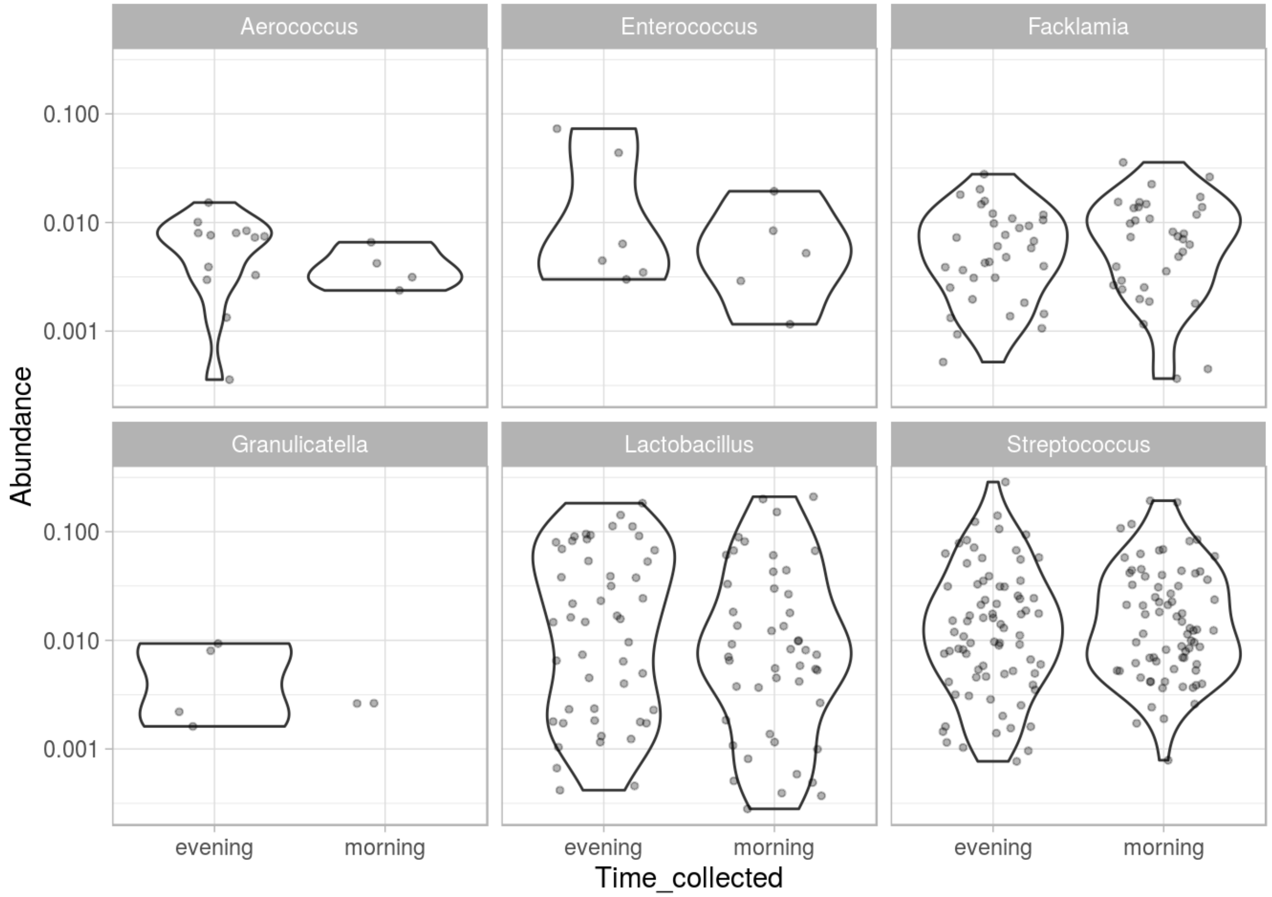

Here we can see the distribution of Genuses in the Order Selenomonadales in our data. If the Order distributions were bimodal, we could see what was contributing to that.

Preprocessing

Before doing multivariate projects, we will add a few columns to the dataset to annotate the plots. In the graph below, change the variable `SS_no` depending on what graph you want to see.

```
qplot(sample_data(ps3ra)$SS_no, geom = "histogram",binwidth=20) + xlab("SS_no")
```

```
## Warning: Removed 3 rows containing non-finite values (stat_bin).
```

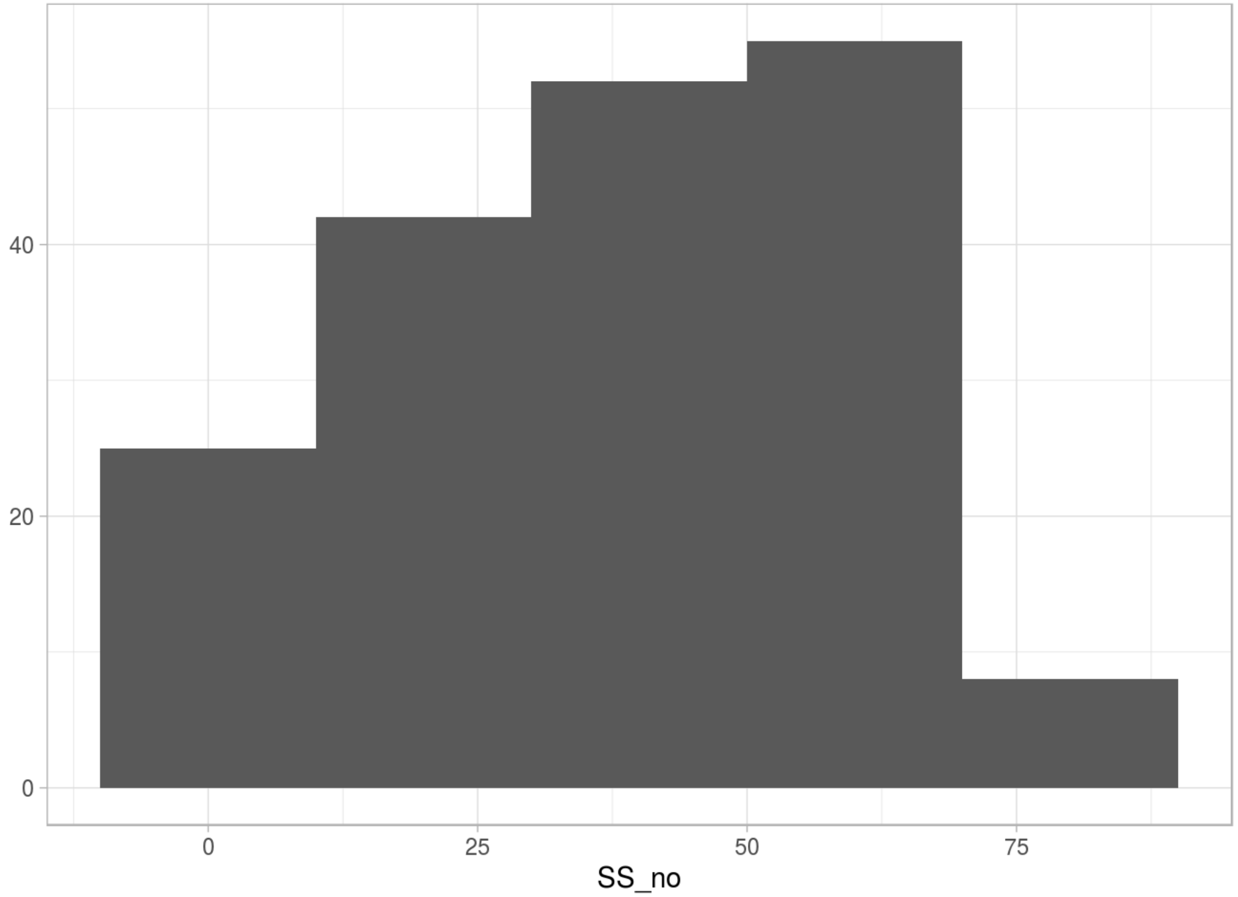


```
qplot(log10(rowSums(otu_table(physeq))),binwidth=0.2) +
  xlab("Logged counts-per-sample")
```

```
## Warning: Removed 8 rows containing non-finite values (stat_bin).
```

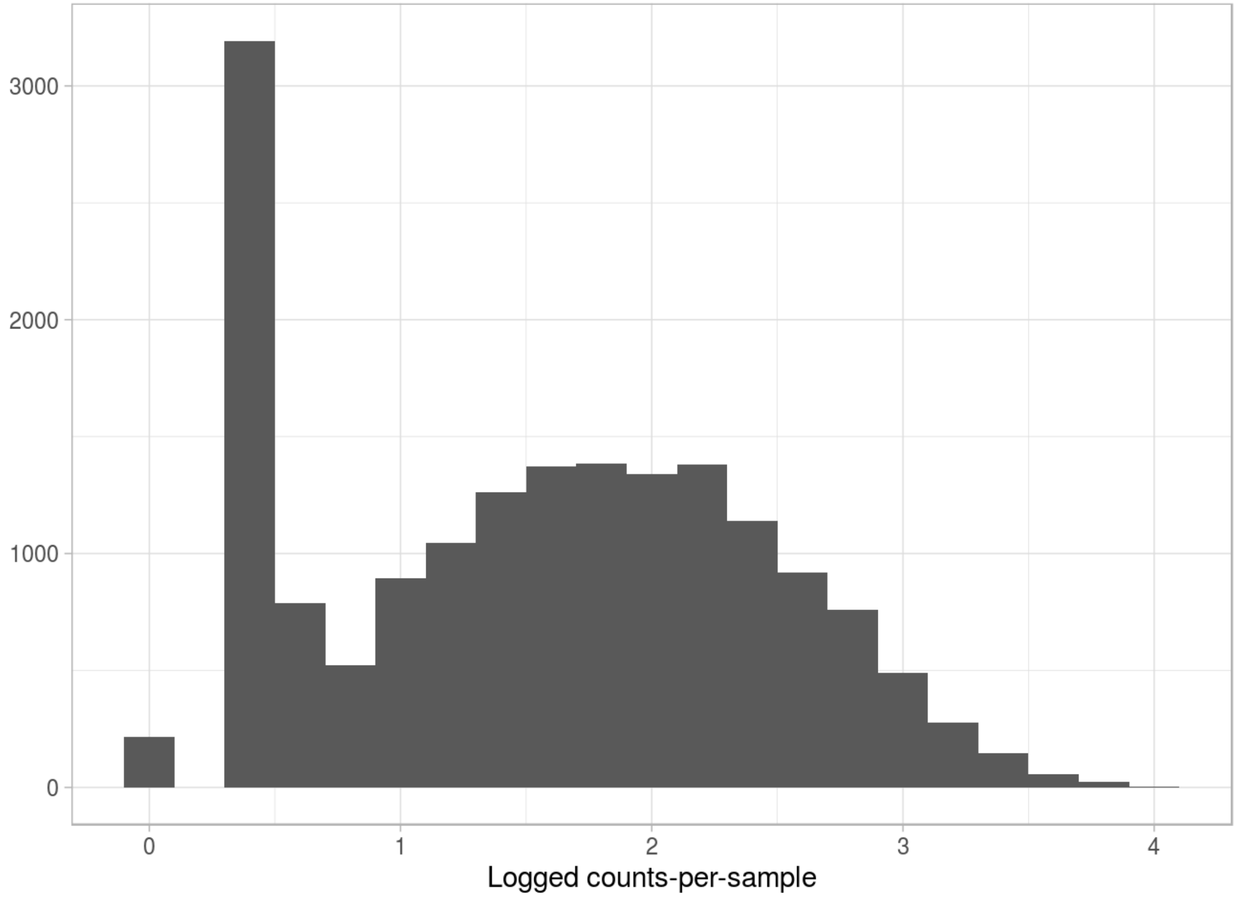

It looks like a `log` transformation may be good for transoforming the data, but there is a left-skew that would not be normal. We will proceed without transofrmation.

PCA

```
### pca my way
#note that we are using the original physeq object, not the one that has relati
ve abundance.
# if you want to do the PCA with relative abundances, use ps3ra
otu_table(ps3) %>% #select(-Species) %>% # remove Species column
  #scale() %>%                 # scale to 0 mean and unit variance
  prcomp() ->                 # do PCA
  pca                         # store result as `pca`
```

The main results from PCA are the standard deviations and the rotation matrix. First, let's plot the data in the principal components. Specifically, we will plot PC2 vs. PC1. The rotated data are available as `pca$x` :

```
# add species information back into PCA data
pca_data <- data.frame(pca$x, Taxa=tax_table(ps3))
ggplot(pca_data, aes(x=PC2, y=PC1)) + geom_point()
```

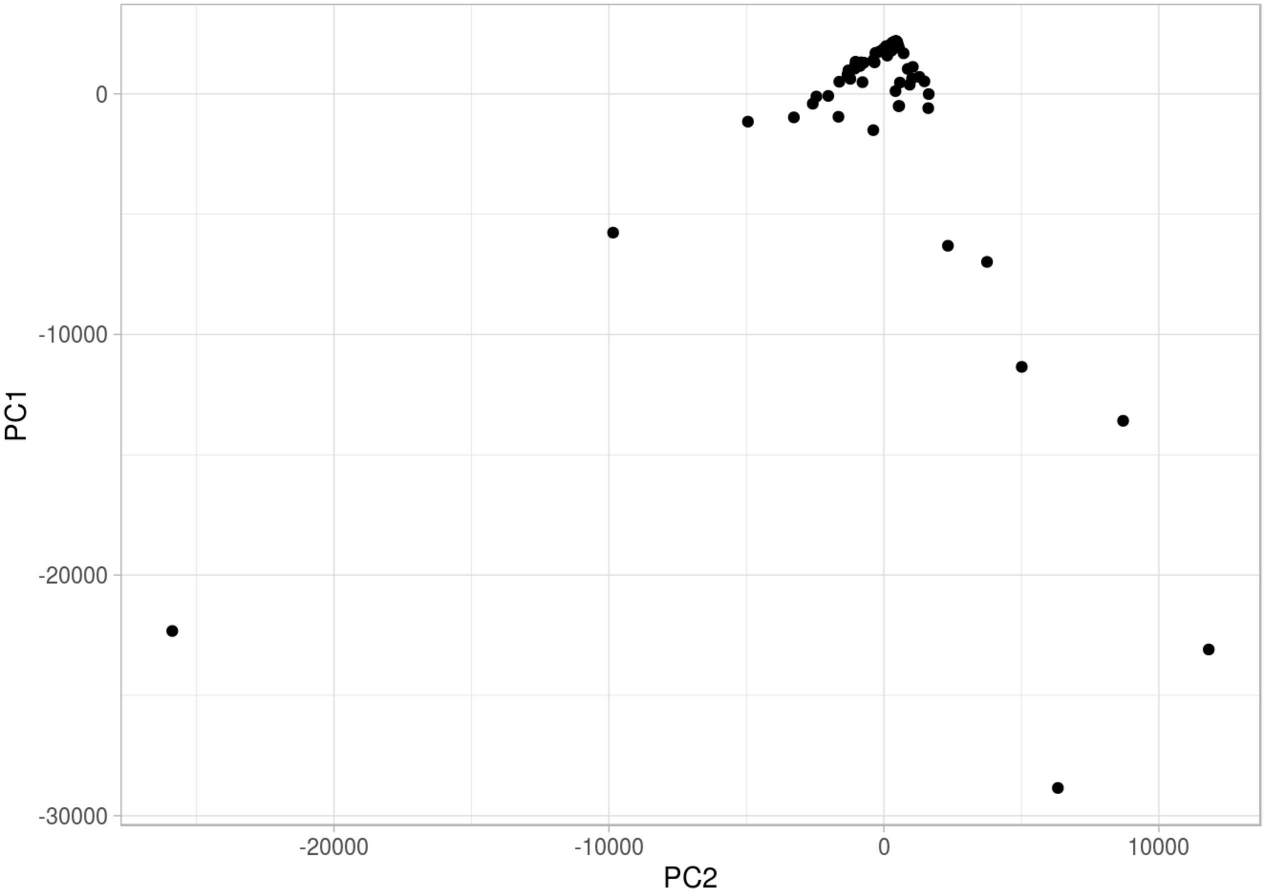

Now lets look at the rotation matrix to understand how each variable is contributing to the observed variation.

```
# capture the rotation matrix in a data frame
rotation_data <- data.frame(pca$rotation, variable=row.names(pca$rotation))
# define a pleasing arrow style
arrow_style <- arrow(length = unit(0.05, "inches"),
                     type = "closed")
# now plot, using geom_segment() for arrows and geom_text for labels
ggplot(rotation_data) +
  geom_segment(aes(xend=PC3, yend=PC2), x=0, y=0, arrow=arrow_style) +
  geom_text(aes(x=PC3, y=PC2, label=variable), hjust=0, size=3, color='red') +
  xlim(-0.3,0.3) +
  ylim(-0.2,0.2) +
  coord_fixed() # fix aspect ratio to 1:1
```

```
## Warning: Removed 2 rows containing missing values (geom_segment).
```

```
## Warning: Removed 2 rows containing missing values (geom_text).
```

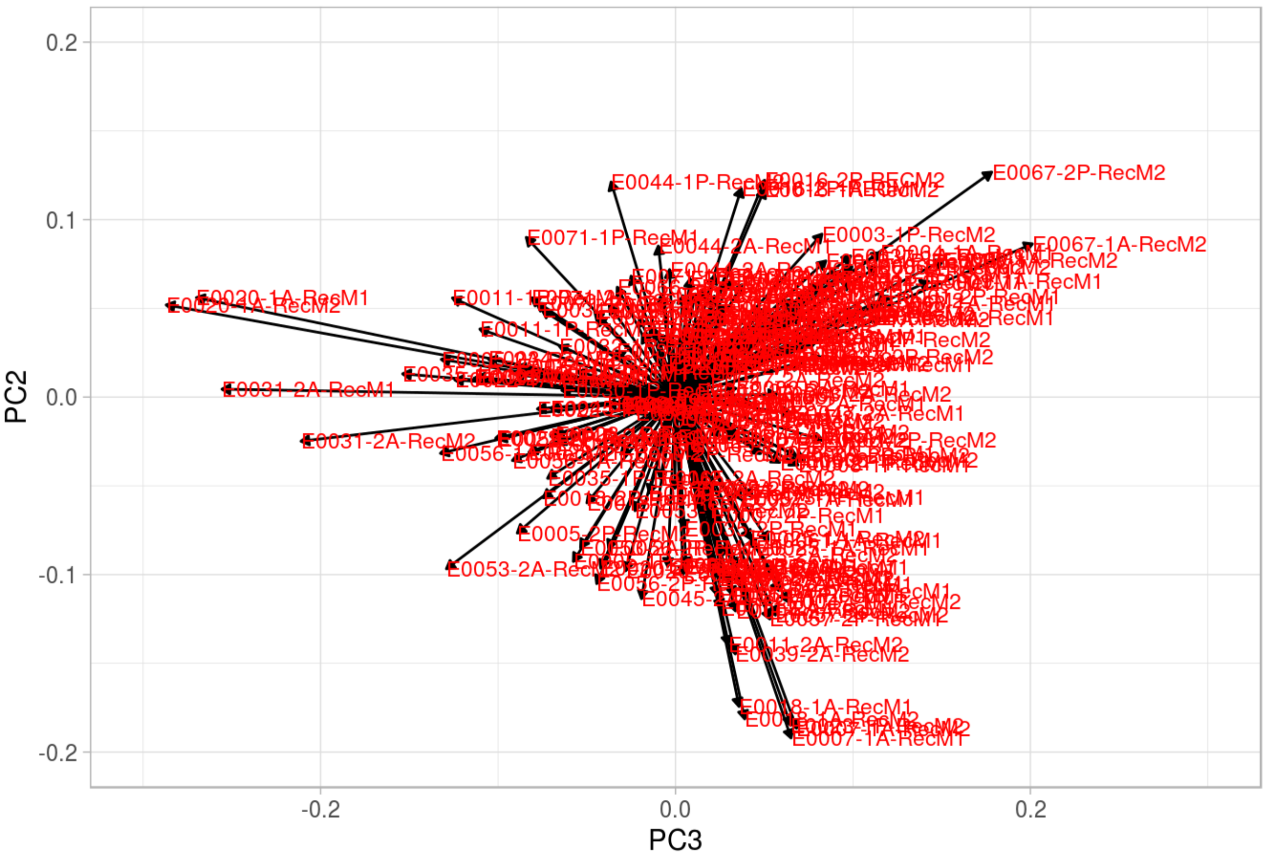

Finally, we want to look at the percent variance explained. The prcomp() function gives us standard deviations (stored in pca$sdev). To convert them into percent variance explained, we square them and then divide by the sum over all squared standard deviations:

```
percent <- 100*pca$sdev^2/sum(pca$sdev^2)

#prep graph of percent variance
perc_data <- data.frame(percent=percent, PC=1:length(percent))
ggplot(perc_data, aes(x=PC, y=percent)) +
  geom_bar(stat="identity") +
  geom_text(aes(label=round(percent, 2)), size=4, vjust=-.5) +
  ylim(0, 20)
```

```
## Warning: Removed 2 rows containing missing values (position_stack).
```

```
## Warning: Removed 2 rows containing missing values (geom_text).
```

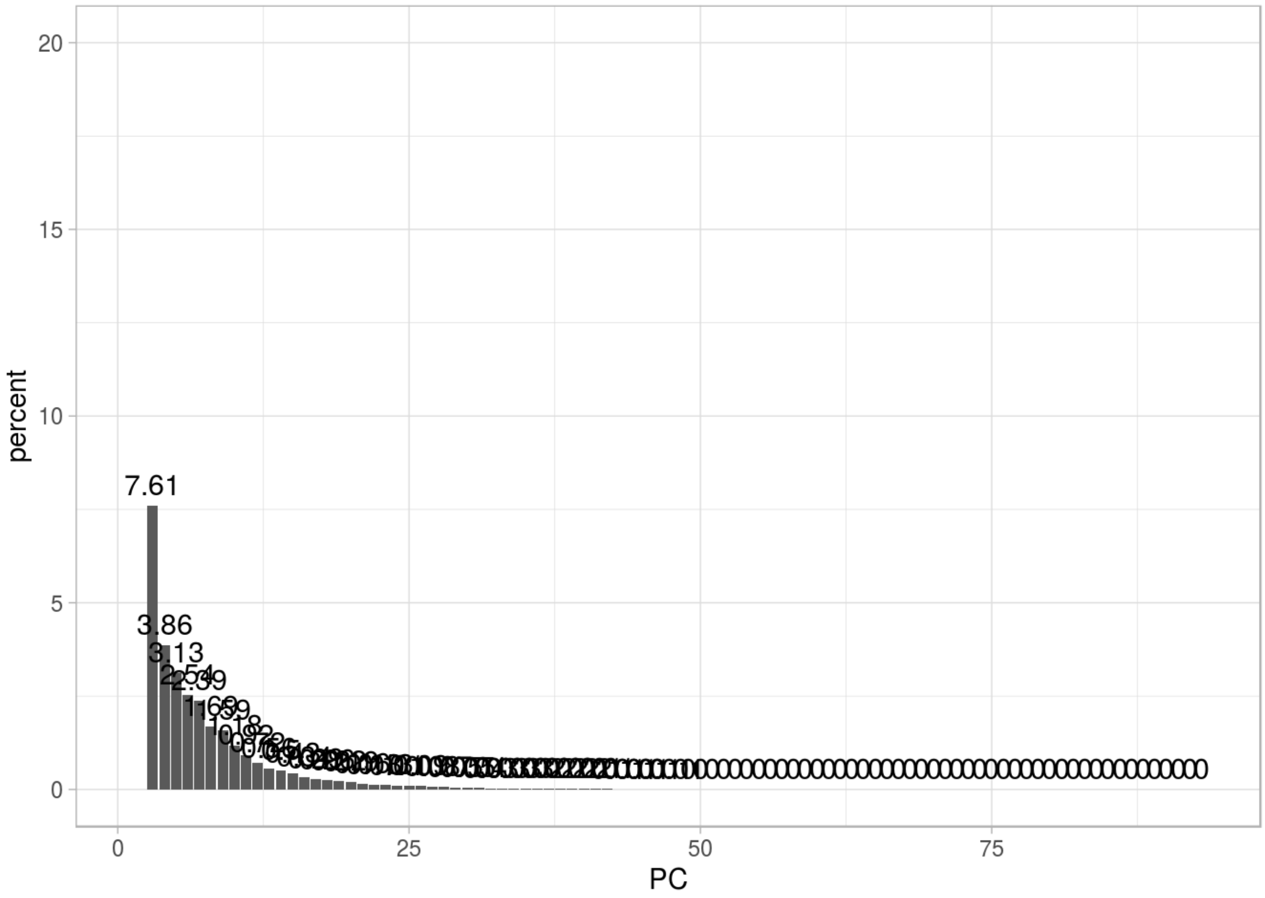

When the variable being plotted is the Subject ID, the percent variance per PC is very low (not over 15% for PC1). This isn't very insightful, so I am going to try the PCA another way.

```
#pslog <- sample_counts(ps3 function(x) log(1 + x))  # use this line for transo
frmation,
otu_table(ps3) <- na.omit( otu_table(ps3) )


out.wuf.log <- ordinate(ps3, method = "MDS", distance = "wunifrac")
evals <- out.wuf.log$values$Eigenvalues
plot_ordination(ps3, out.wuf.log, color = "IBS") +
  coord_fixed(sqrt(evals[2] / evals[1]))+
  scale_color_manual(values=tol(2))+
  labs(title="PCA, outliers included, not transformed")
```

We are going to take out the control samples to see if that changes the results at all.

```
outliers <- c("Postive-control", "negCtrl", "negative-control")
ps3 <- prune_samples(!(sample_names(ps3) %in% outliers), ps3)
```

Now we are going to try the PCA again and hope for better results.

```
#created binned age
sample_data(ps3)$age_binned <- cut(sample_data(ps3)$Age,
                          breaks = c(20, 30, 40, 50,60))
levels(sample_data(ps3)$age_binned) <- list(Twenties="(20,30]", Thirties="(30,4
0]", Fourties="(40,50]", Fifties="(50,60]")

out.pcoa<- ordinate(ps3,  method = "MDS", distance = "bray")
evals <- out.pcoa$values[,1]
plot_ordination(ps3, out.pcoa, color = "IBS",
                  shape = "Scheduled_shift") +
  labs( title="PCA, controls removed")+
  coord_fixed(sqrt(evals[2] / evals[1])) +
  scale_color_manual(values=as.vector(tol(4)))+
  #scale_shape_manual(values=c(7, 0)) +
  theme_light()
```

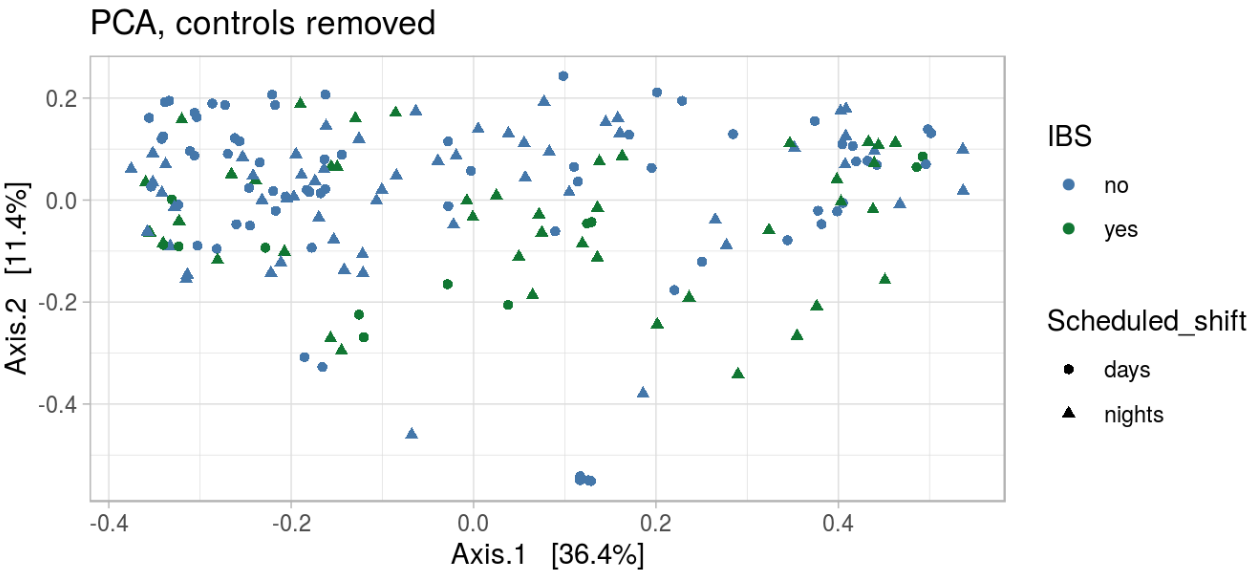

```
per_var <- round( out.pcoa$values$Relative_eig*100, 2)
```

This graph does not contain any clear clustering. I've examined by age (binned by 10 years), IBS, time collected, and scheduled_shift. The present graph shows IBS status and scheduled shift (days vs nights). Further, there is low percent variance explained (36.36 for Axis 1, `r per_var[2] for Axis 2). Ideally, The percent variance explained should be over 50% for one of the axes.

We are going to look at a different kind of PCA now - we will examine which taxa are responsible for Axis 1 and Axis 2. Given that our PCA does not show clear grouping, we can anticipate this graph to appear messy and for phyla not to fall cleanly along a certain axis.

```
plot_ordination(ps3, out.pcoa, type = "species", color = "Phylum") +
  coord_fixed(sqrt(evals[2] / evals[1]))+
  scale_color_manual(values=stepped2(18)) +
  labs(title = "Species level Ordination Plot")
```

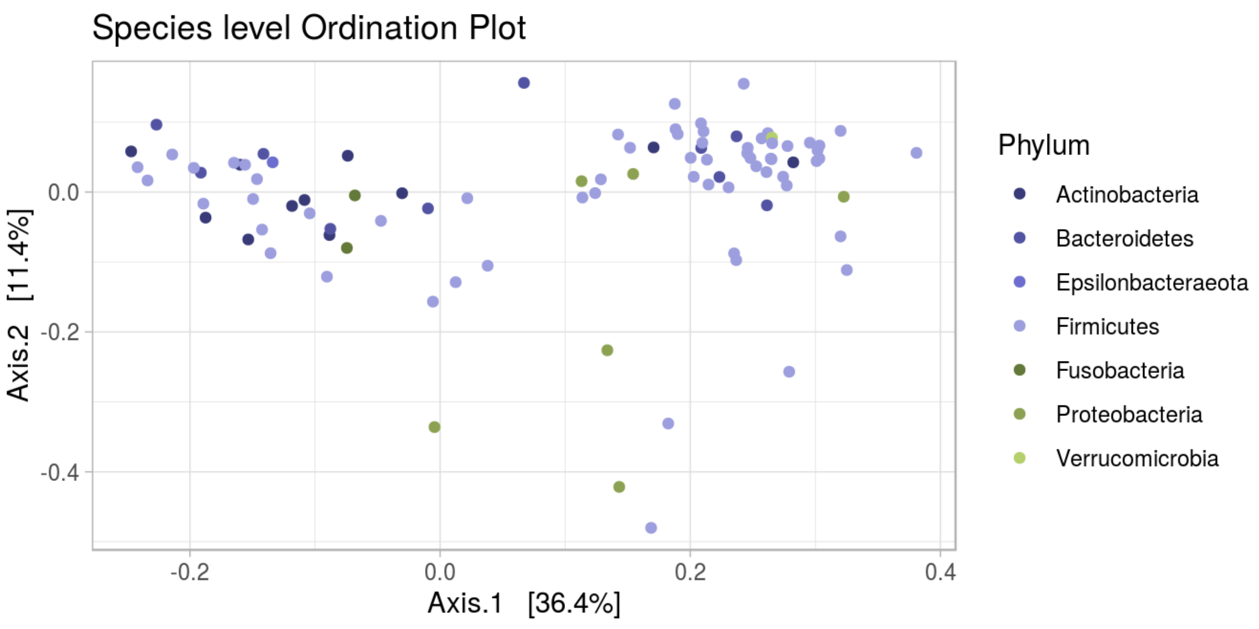

As suspected, this PCA is not more insightful. It is messy, and the bacteria do not appear to contribute to variance in any pattern.

But we won't give up that easily! Let's look at a PCA using Weighted Unifrac.

```
out.wuf.log <- ordinate(ps3, method = "PCoA", distance ="wunifrac")
evals <- out.wuf.log$values$Eigenvalues
plot_ordination(physeq, out.wuf.log, color = "age_binned") +
  coord_fixed(sqrt(evals[2] / evals[1])) +
  scale_color_manual(values=tol(4))
```

```
## Warning in plot_ordination(physeq, out.wuf.log, color = "age_binned"):
## Color variable was not found in the available data you provided.No color
## mapped.
```

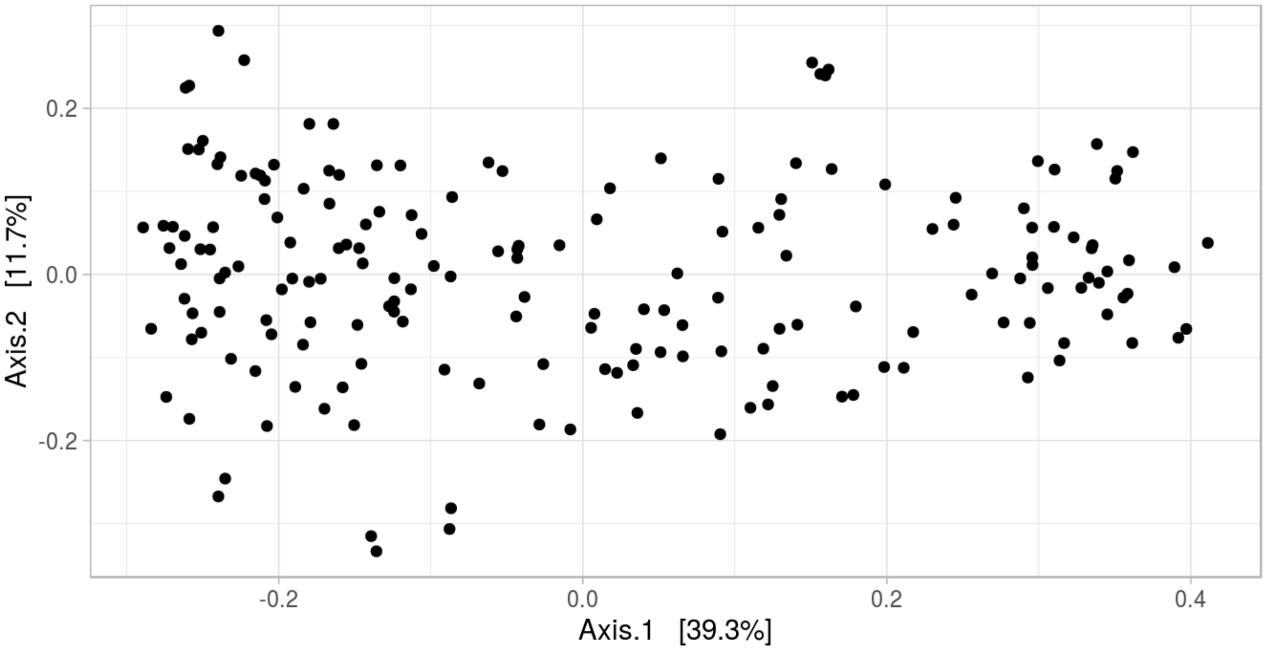

Again, this PCA does not show any clear clustering, and even less percent variance is explained than before!

Bar plot

First I need to set up the color palette.

```
#large palette sorted by difference
pal_diff_93 <- c("#d95297",
"#6ae367",
"#733d39",
"#66d03e",
"#89596f",
"#99c840",
"#952f60",
"#54aa36",
"#602542",
"#318124",
"#371589",
"#494626",
"#6775ee",
"#d38869",
"#4823ae",
"#7e8d26",
"#447893",
"#ea5d18",
"#3c7d78",
"#d05827",
"#3a6e4b",
"#c057c2",
"#b9aa87",
"#4c50c9",
"#dc9960",
"#492e65",
"#c5ea29",
"#9c2e46",
"#abd533",
"#d6a1b7",
"#47dd74",
"#514d71",
"#c5bc43",
"#e13e79",
"#4d6b2e",
"#d248eb",
"#8dccb4",
"#e23128",
"#6fb1e0",
"#d74939",
"#a0b5d8",
"#dc9328",
"#61cdd6",
"#d73e50",
"#6894d9",
```

```
"#954f1c",
"#9c5ff1",
"#d7c38d",
"#462c82",
"#93e37c",
"#883b82",
"#4dad55",
"#cc8bd3",
"#75e73a",
"#ce84ad",
"#dec92e",
"#be99cf",
"#9d7b1e",
"#e135ac",
"#cbbd71",
"#9e33c8",
"#499251",
"#bc65e2",
"#806b27",
"#653dd9",
"#998653",
"#724fb1",
"#5b7a28",
"#e279d8",
"#5d4b1c",
"#5486e1",
"#cb6563",
"#4dd5b5",
"#7a2f20",
"#812ca2",
"#5fcd92",
"#c754a4",
"#c0cf66",
"#4e58a3",
"#8fbe8c",
"#d53bc6",
"#a4d68f",
"#a67dde",
"#dcae57",
"#5c6da1",
"#b3442e",
"#53e393",
"#de738f",
"#abcb73",
"#e72754",
"#cd8e86",
```

```
"#c3742e",
"#df6a5c")

#same palette sorted by hue
pal_hue_93 <- c("#e13e79", "#de738f", "#9c2e46","#e72754","#d73e50","#cb6563","
#733d39","#cd8e86","#df6a5c","#d74939",
               "#e23128","#7a2f20","#b3442e","#d05827","#d38869","#ea5d18","#95
4f1c","#c3742e","#dc9960","#dc9328",
               "#dcae57","#9d7b1e","#5d4b1c","#806b27","#998653","#b9aa87","#d7
c38d","#dec92e","#cbbd71","#c5bc43",
               "#494626","#7e8d26","#c0cf66","#c5ea29","#abd533","#99c840","#5b
7a28","#abcb73","#4d6b2e","#75e73a","#66d03e",
               "#54aa36","#a4d68f","#93e37c","#318124","#6ae367","#8fbe8c","#4d
ad55","#499251","#47dd74","#3a6e4b",
               "#53e393","#5fcd92","#8dccb4","#4dd5b5","#3c7d78","#61cdd6","#44
7893","#6fb1e0","#a0b5d8","#6894d9","#5486e1",
               "#5c6da1","#4e58a3","#6775ee","#514d71","#4c50c9","#653dd9","#46
2c82","#4823ae","#724fb1","#371589",
               "#a67dde","#9c5ff1","#492e65","#be99cf","#bc65e2","#9e33c8","#81
2ca2","#d248eb","#cc8bd3","#c057c2",
               "#883b82","#e279d8","#d53bc6","#c754a4","#e135ac","#ce84ad","#89
596f","#d95297","#602542","#d6a1b7","#952f60")
```

Finally, let’s develop a bar graph as is typical for this work.

```
#the default is plot_bar but I don't like this graph, so we will do a little ex
tra work to get a prettier graph
# first we need to make a tidy dataset that is not a phyloseq object
## We want to does this with the relative abundance to make a prettier graph so
using ps3ra instead od ps3
ps <- psmelt(ps3ra) #join by Sample
```

```
## Warning in psmelt(ps3ra): The sample variables:
## Sample
##  have been renamed to:
## sample_Sample
## to avoid conflicts with special phyloseq plot attribute names.
```

```
ggplot(ps, aes(x=Sample, y=Abundance, fill=Phylum, order=as.factor(Phylum)))+
  geom_bar(stat = "identity", color = "black")+
  ggtitle("All Samples - Relative Abuncance, sorted by Phylum abundance") +
 theme(#legend.position = "none",
      axis.text.x = element_blank()) +
  scale_fill_manual(values=tol(12))
```

```
## Warning: Removed 279 rows containing missing values (position_stack).
```

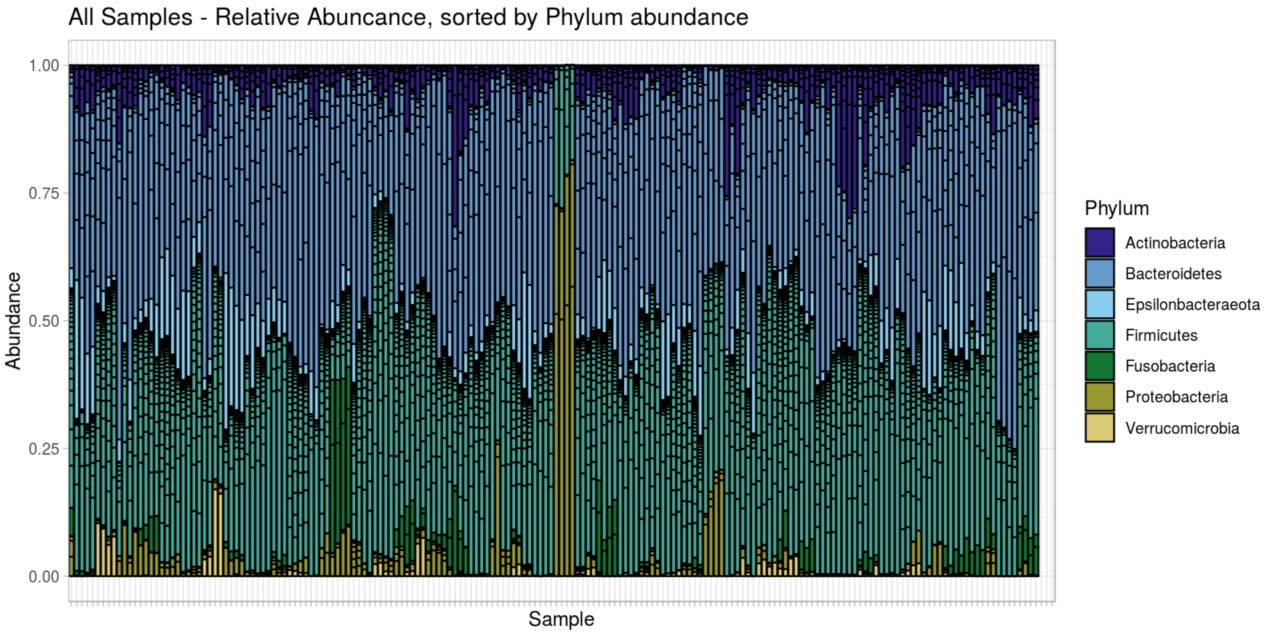


```
ggplot(ps, aes(x=Sample, y=Abundance, fill=Class, order=as.factor(Phylum)))+
  geom_bar(stat = "identity", color = "black")+
  ggtitle("All Samples - Relative Abuncance, sorted by Class abundance") +
 theme(#legend.position = "none",
       axis.text.x = element_blank()) +
  scale_fill_manual(values=tol(12))
```

```
## Warning: Removed 279 rows containing missing values (position_stack).
```

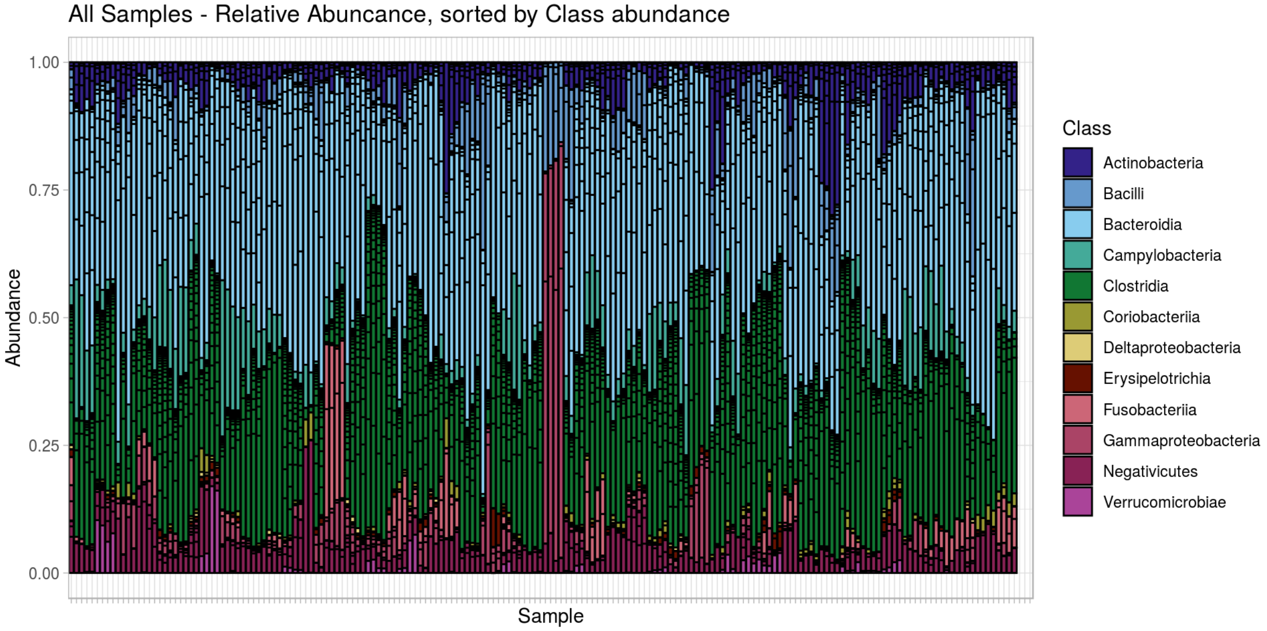


```
ggplot(ps, aes(x=Sample, y=Abundance, fill=Order, order=as.factor(Phylum)))+
  geom_bar(stat = "identity", color = "black")+
  ggtitle("All Samples - Relative Abuncance, sorted by Order abundance") +
 theme(#legend.position = "none",
       axis.text.x = element_blank()) +
  scale_fill_manual(values=stepped2(17))
```

```
## Warning: Removed 279 rows containing missing values (position_stack).
```

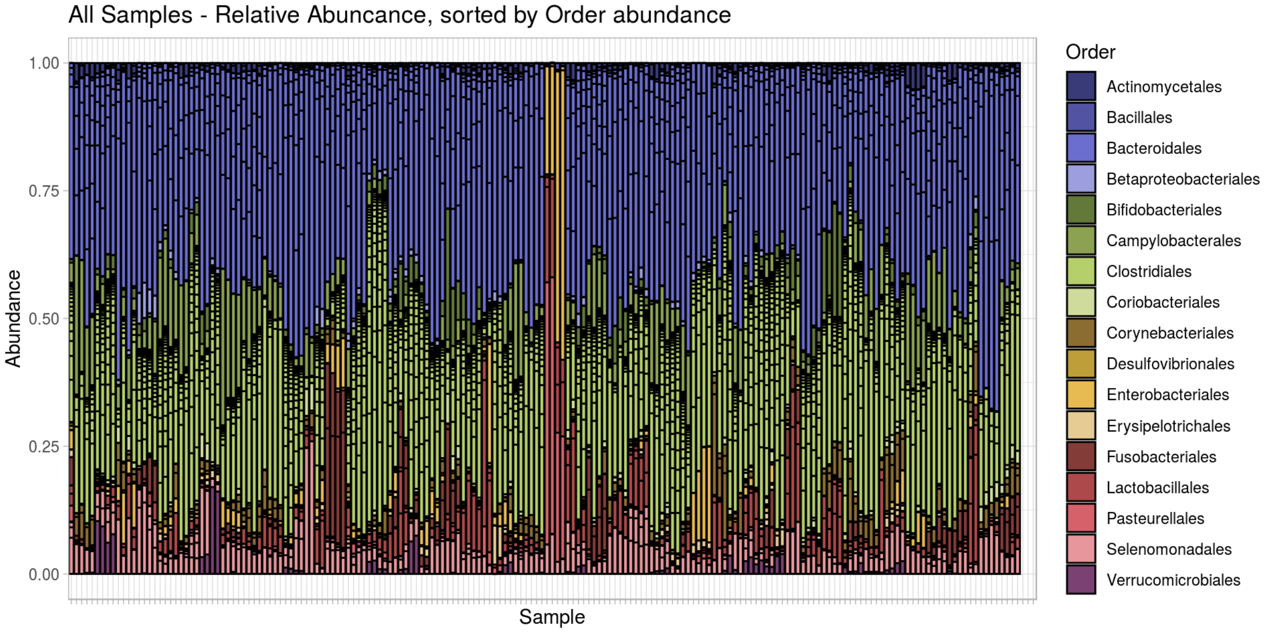

```
ggplot(ps, aes(x=Sample, y=Abundance, fill=Family, order=as.factor(Phylum)))+
  geom_bar(stat = "identity", color = "black")+
  ggtitle("All Samples - Relative Abuncance, sorted by Family abundance") +
 theme(#legend.position = "none",
      axis.text.x = element_blank()) +
  scale_fill_manual(values=pal_diff_93)
```

```
## Warning: Removed 279 rows containing missing values (position_stack).
```

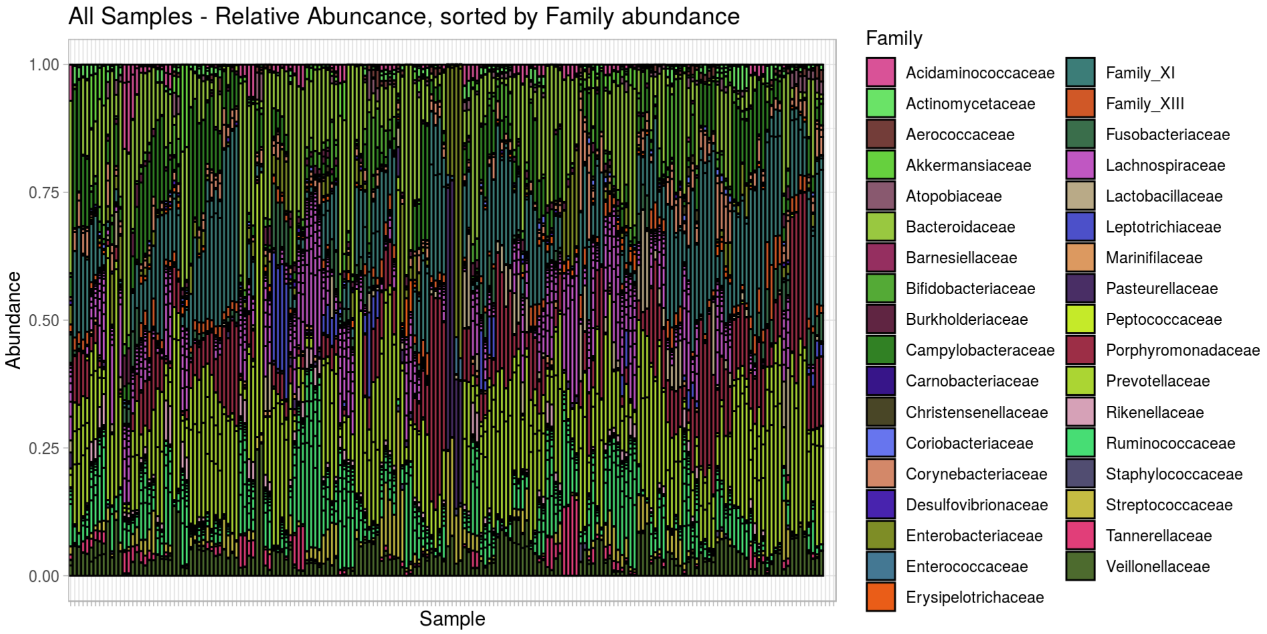


```
ggplot(ps, aes(x=Sample, y=Abundance, fill=Genus, order=as.factor(Phylum)))+
  geom_bar(stat = "identity", color = "black")+
  ggtitle("All Samples - Relative Abuncance, sorted by Genus abundance") +
 theme(legend.position = "none",
      axis.text.x = element_blank()) +
  scale_fill_manual(values=pal_hue_93)
```

```
## Warning: Removed 279 rows containing missing values (position_stack).
```

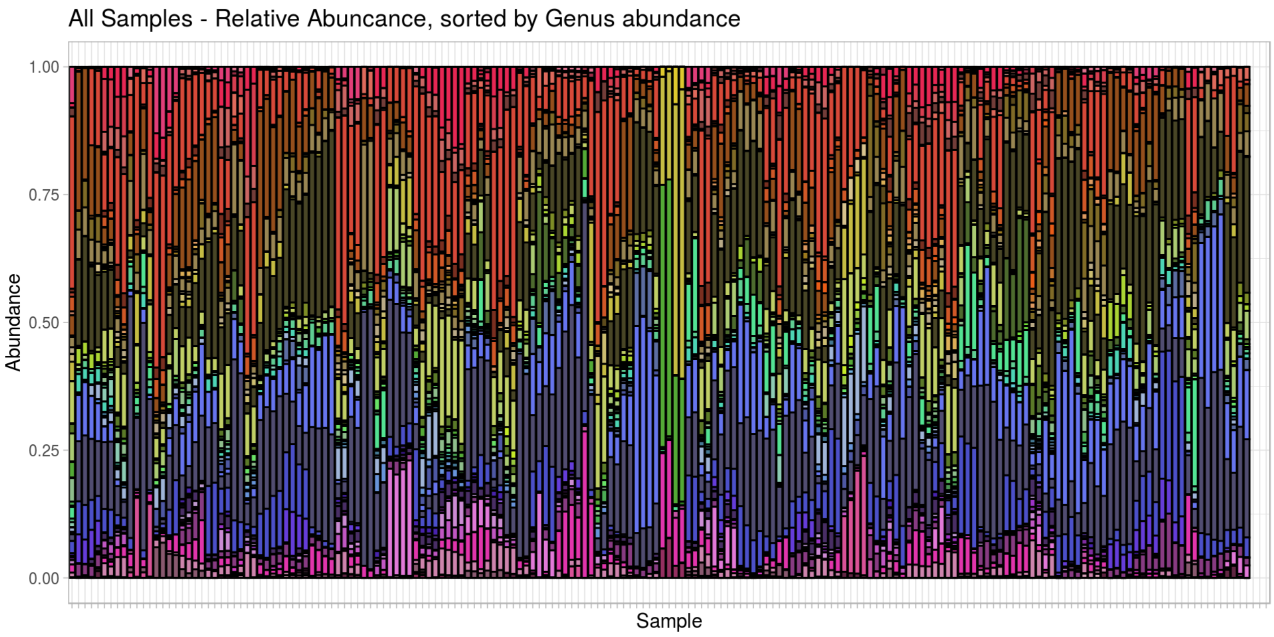


```
#get the legend for genus plot
w_legend_2 <-ggplot(ps, aes(x=Sample, y=Abundance, fill=Genus, order=as.factor
(Phylum)))+
  geom_bar(stat = "identity", color = "black")+
  ggtitle("All Samples - Relative Abuncance, sorted by Genus abundance") +
 theme(axis.text.x = element_blank(), legend.position = "bottom") +
  scale_fill_manual(values=pal_hue_93) +
  guides(fill=guide_legend(nrow=20, byrow=TRUE))

genus_legend<- g_legend(w_legend_2) #gridExtra
```

```
## Warning: Removed 279 rows containing missing values (position_stack).
```

```
grid.arrange(genus_legend)
```

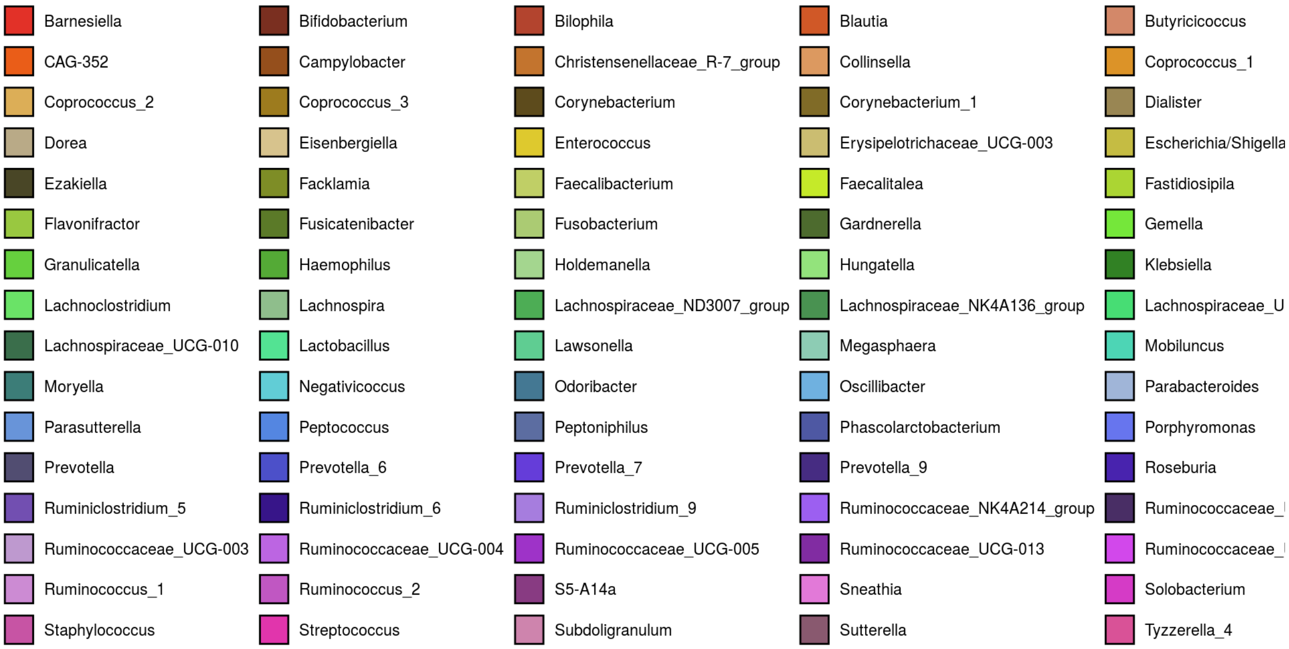
Barnesiella
Bifidobacterium
Bilophila
Blautia
Butyricicoccus
CAG-352
Campylobacter
Christensenellaceae_R-7_group
Collinsella
Coprococcus_1
Coprococcus_2
Coprococcus_3
Corynebacterium
Corynebacterium_1
Dialister
Dorea
Eisenbergiella
Enterococcus
Erysipelotrichaceae_UCG-003
Escherichia/Shigella
Ezakiella
Facklamia
Faecalibacterium
Faecalitalea
Fastidiosipila
Flavonifractor
Fusicatenibacter
Fusobacterium
Gardnerella
Gemella
Granulicatella
Haemophilus
Holdemanella
Hungatella
Klebsiella
Lachnoclostridium
Lachnospira
Lachnospiraceae_ND3007_group
Lachnospiraceae_NK4A136_group
Lachnospiraceae_U
Lachnospiraceae_UCG-010
Lactobacillus
Lawsonella
Megasphaera
Mobiluncus
Moryella
Negativicoccus
Odoribacter
Oscillibacter
Parabacteroides
Parasutterella
Peptococcus
Peptoniphilus
Phascolarctobacterium
Porphyromonas
Prevotella
Prevotella_6
Prevotella_7
Prevotella_9
Roseburia
Ruminiclostridium_5
Ruminiclostridium_6
Ruminiclostridium_9
Ruminococcaceae_NK4A214_group
Ruminococcaceae_
Ruminococcaceae_UCG-003
Ruminococcaceae_UCG-004
Ruminococcaceae_UCG-005
Ruminococcaceae_UCG-013
Ruminococcaceae_
Ruminococcus_1
Ruminococcus_2
S5-A14a
Sneathia
Solobacterium
Staphylococcus
Streptococcus
Subdoligranulum
Sutterella
Tyzzerella_4
