# Supplementary material for: Shiftwork, functional bowel symptoms, and the microbiome
Source: PeerJ. 2021 May 11;9:e11406. doi: 10.7717/peerj.11406 (PMC8121053; doi:10.7717/peerj.11406)
Supplement: Supplemental Information 2 [file peerj-09-11406-s002.pdf]

Note: this code was modified from a web tutorial: https://benjjneb.github.io/dada2/tutorial.html

supernova 16s Notebook

```
library(dada2)
```

```
## Loading required package: Rcpp
```

```
library(decontam)
library(ggplot2)
library(jsonlite)
library(lattice)
library(lemon)
library(reshape)
library(zoo)
```

```
##
## Attaching package: 'zoo'

## The following objects are masked from 'package:base':
##
##     as.Date, as.Date.numeric
```

```
knit_print.data.frame <- lemon_print
PROJECT_DIR = "/home/rpetit/projects/supernova-16s"
setwd(PROJECT_DIR)
getN <- function(x) sum(getUniques(x))
```

Input FASTQs

Input FASTQs had adapters removed using BBDuk prior to DADA2 analysis. This was accomplished with the following command:

The adapter cleaned FASTQ files were stored in `data/adapter-cleaned`

Dada2 Analysis

Setting Up Files and Naming

```
# Input FASTQs
fastq_path = paste0(PROJECT_DIR, "/data/adapter-cleaned")
fastq_r1 <- sort(
  list.files(fastq_path, pattern="_R1.fastq.gz", full.names = TRUE)
)
fastq_r2 <- sort(
  list.files(fastq_path, pattern="_R2.fastq.gz", full.names = TRUE)
)
sample_names <- sapply(strsplit(basename(fastq_r1), "_"), `[`, 1)
```

Plot Project FASTQ Quality Profile

```
json_r1 <- sort(list.files(fastq_path, pattern="_R1.json", full.names = TRUE))
json_r2 <- sort(list.files(fastq_path, pattern="_R2.json", full.names = TRUE))
r1_profile = NULL
r2_profile = NULL
for (i in 1:length(json_r1)){
  r1_df = as.data.frame(fromJSON(txt=json_r1[i])$per_base_quality)
  r2_df = as.data.frame(fromJSON(txt=json_r2[i])$per_base_quality)
  if (is.null(r1_profile)) {
    r1_profile = r1_df
    r2_profile = r2_df
  }
  else {
    r1_profile = rbind(r1_profile, r1_df)
    r2_profile = rbind(r2_profile, r2_df)
  }
}

colnames(r1_profile) <- as.integer(gsub("X", "", colnames(r1_profile)))
colnames(r2_profile) <- as.integer(gsub("X", "", colnames(r2_profile)))
means = data.frame(
  position=1:ncol(r1_profile),
  R1=as.data.frame(colMeans(r1_profile))[,1],
  R2=as.data.frame(colMeans(r2_profile))[,1]
)
means$"R1 Rolling Mean" <- rollmean(means$R1, 5, fill="extend")
means$"R2 Rolling Mean" <- rollmean(means$R2, 5, fill="extend")
melted_vals = melt(
  means,
  id.vars = "position",
  measure.vars = c("R1", "R2", "R1 Rolling Mean", "R2 Rolling Mean")
)
p <- ggplot(melted_vals, aes(x=position, y=value)) +
  geom_line() +
  facet_wrap(~ variable, ncol=2) +
  xlab("Base Position") +
  ylab("PHRED Score")
print(p)
```

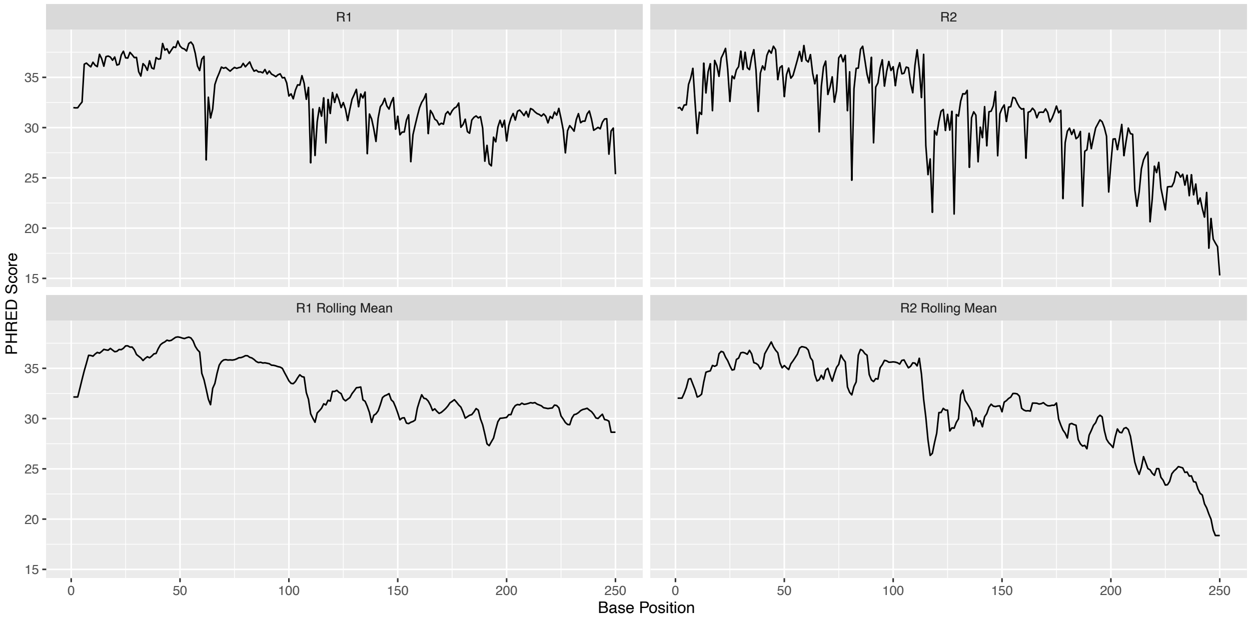

With these plots, we can get a general idea of the per-base mean quality across all samples in the project. There are two plots for forward (R1) and reverse (R2) reads. The top plots ("R1" and "R2") are the per-base mean quality across all samples, and the bottom plots ("R1 Rolling Mean" and "R2 Rolling Mean") use a rolling mean that is based on a centered 5bp sliding window.

As expected the quality tails off in the R2 reads, but overall the quality is good.

Plot Sample FASTQ Quality Profiles (Subset)

Below is an example of the quality profile for a single sample. At the end of this document you can find the quality profile for all 111 samples available in this project.

```
print(plotQualityProfile(c(fastq_r1[1], fastq_r2[1])))
```

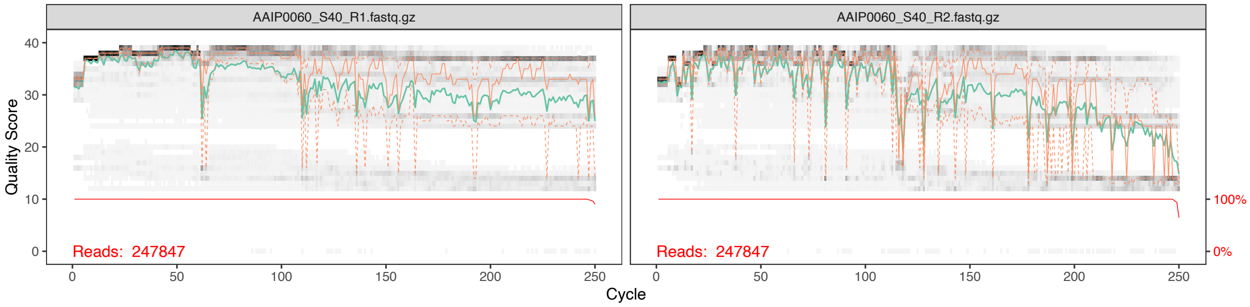

Filter and Trim

For this project the V1 and V2 region of the 16s rRNA gene was sequenced. This region is roughly ~360bp and our project includes 2x250 Illumina MiSeq sequencing. For filtering and trimming we need to maintain a total length greater than 360bp plus a 20bp overlap, so ~380bp. Given 2x250bp reads, this gives 120bp to play with for trimming.

```
filt_r1 <- file.path(
  PROJECT_DIR, "data", "filtered", paste0(sample_names, "_R1_filt.fastq.gz")
)
filt_r2 <- file.path(
  PROJECT_DIR, "data", "filtered", paste0(sample_names, "_R2_filt.fastq.gz")
)
names(filt_r1) <- sample_names
names(filt_r2) <- sample_names
out <- as.data.frame(
  filterAndTrim(fastq_r1, filt_r1, fastq_r2, filt_r2, truncLen=c(250,210),
                minLen=190, maxN=0, maxEE=c(2,5), truncQ=2, rm.phix=TRUE,
                compress=TRUE, multithread=TRUE, verbose=FALSE)
)
filtered_stats <- out
colnames(filtered_stats) <- c("input", "filtered")
rownames(filtered_stats) <- sample_names
filtered_stats$percent_filtered_out <- 1 - (filtered_stats$filtered / filtered_stats$input)
```

Using the quality profile of the complete set of samples, the following parameters were using for `filterAndTrim` (fitering and trimming):

Parameters	Description
`truncLen=c(250,210)`	Trim length of forward (250bp) and reverse (210bp) reads
`minLen=185`	Remove reads that are less than 190bp after trimming
`maxN=0`	Filter reads containing ambiguous (e.g. N) base calls
`maxEE=c(2,5)`	Filter reads with more than expected basecall errors (R1: 2, R2: 5)
`truncQ=2`	Trim reads at the position with a PHRED score of Q2
`rm.phix=TRUE`	Remove any reads matching PhiX (Illumina control)
`compress=TRUE`	Gzip output FASTQs
`multithread=TRUE`	Use multiple processors
`verbose=FALSE`	Do not output status messages

```
print(histogram(
  filtered_stats$percent_filtered_out,
  nint=50,
  type="count",
  xlab="Percent of Reads Filtered Out"
))
```

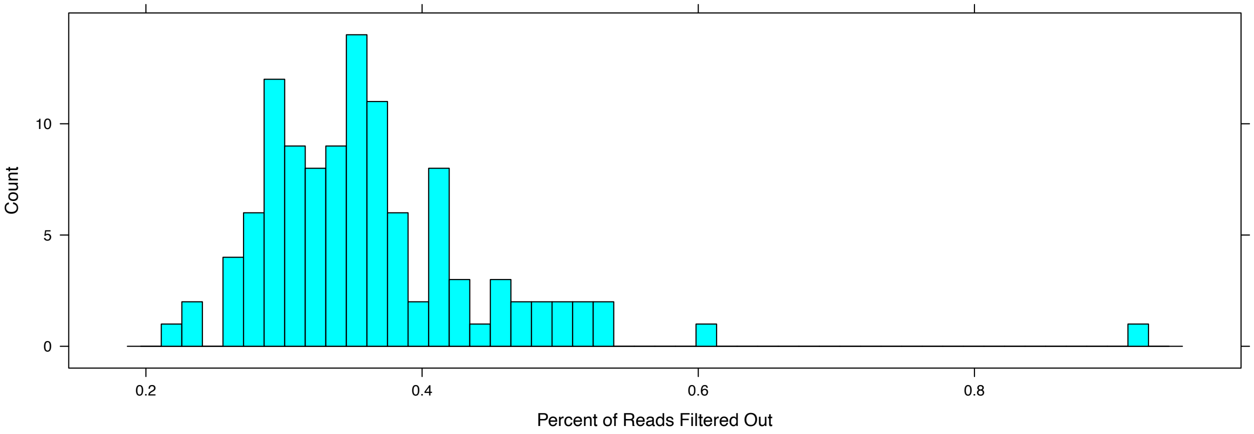


```r
summary(filtered_stats$percent_filtered_out)
```

```
##    Min. 1st Qu.  Median    Mean 3rd Qu.    Max.
##  0.2238  0.3042  0.3485  0.3633  0.3939  0.9133
```

Above is histogram and summary of the percentage of reads filtered out by `filterAndTrim` for each sample. Ideally it should be left-skewed, meaning most reads passed filtering and trimming and were not filtered out. If you find most of the reads are being filtered out, please review the `filterAndTrim` parameters used above. In cases were a large percentage of reads were filtered out, manual review might be necessary to determine the cause.

Below is the top 10 samples with the most reads filtered out to review.

```r
head(filtered_stats[order(filtered_stats$percent_filtered_out, decreasing=TRUE),], n=10)
```

```
##           input filtered percent_filtered_out
## AAIP0064 196609    17039            0.9133356
## LCIP0045 162647    64724            0.6020584
## ECOP0011 384927   179039            0.5348754
## AAIP0093 218572   102759            0.5298620
## LCIP0013 298525   144575            0.5157022
## LCIP0076 193939    94509            0.5126870
## LAIP0007 250777   123132            0.5089980
## BLANK7    12662     6291            0.5031591
## EAIP0032  14378     7437            0.4827514
## ECIP0257 264601   137051            0.4820466
```

Determine and Plot Error Rates

```r
error_r1 <- learnErrors(filt_r1, multithread=TRUE, verbose=FALSE)
```

```
## 131659000 total bases in 526636 reads from 5 samples will be used for learning the error rates.
```

```r
error_r2 <- learnErrors(filt_r2, multithread=TRUE, verbose=FALSE)
```

```
## 110593560 total bases in 526636 reads from 5 samples will be used for learning the error rates.
```

```r
print(plotErrors(error_r1, nominalQ=TRUE))
```

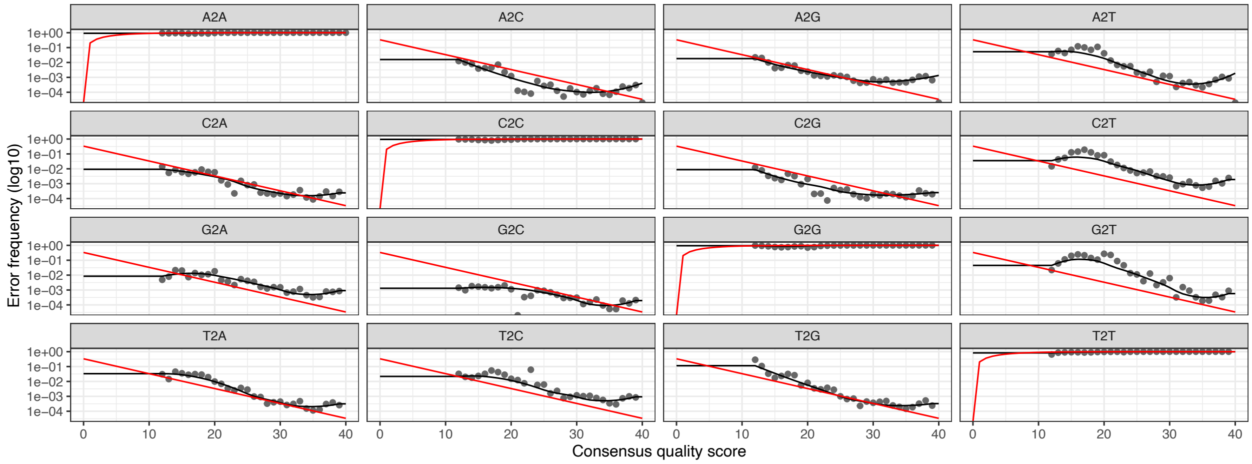

In the plot above the dots are the observed error rates for each consensus quality score. The redline shows the expected error rates based and the black line fit to the observed error rates.

Sample Inference (Denoise) and Merge Reads

```r
dada_r1 <- dada(filt_r1, err=error_r1, multithread=TRUE, verbose=FALSE)
dada_r2 <- dada(filt_r2, err=error_r2, multithread=TRUE, verbose=FALSE)
merged_reads <- mergePairs(dada_r1, filt_r1, dada_r2, filt_r2, verbose=TRUE)
print(histogram(
  sapply(merged_reads, getN) / filtered_stats$filtered,
  nint=50,
  endpoints=c(0,1),
  type="count",
  xlab="Percent of Reads Merged"
))
```

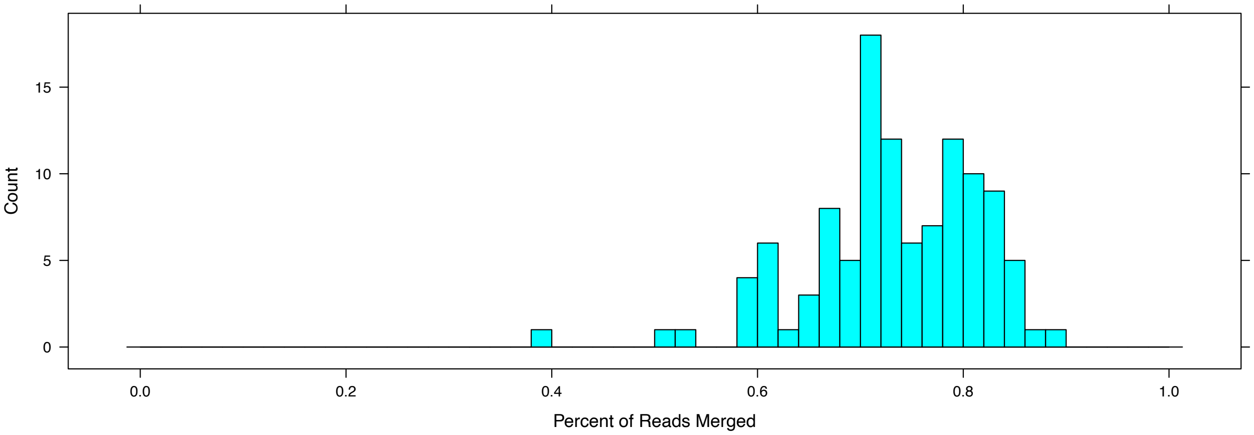


```r
summary(sapply(merged_reads, getN) / filtered_stats$filtered)
```

```
##    Min. 1st Qu.  Median    Mean 3rd Qu.    Max.
##  0.3952  0.6903  0.7321  0.7319  0.7958  0.8911
```

For the above histogram (and summary stats), we are expecting it to be right-skewed, that is to say most of the read pairs were merged. If the percentage of read pairs merged is low across all samples, it might be necessary to review the parameters used with `filterAndTrim`. By default, `mergePairs` requires only a 12bp overlap to merge read pairs. Please keep this in mind if it does become necessary to adjust `filterAndTrim` parameters.

Construct Amplicon Sequence Variant (ASV) Table

```
original_sequence_table <- makeSequenceTable(merged_reads)
print(histogram(
  nchar(getSequences(original_sequence_table)),
  nint=50,
  type="count",
  xlab="Sequence Length"
))
```

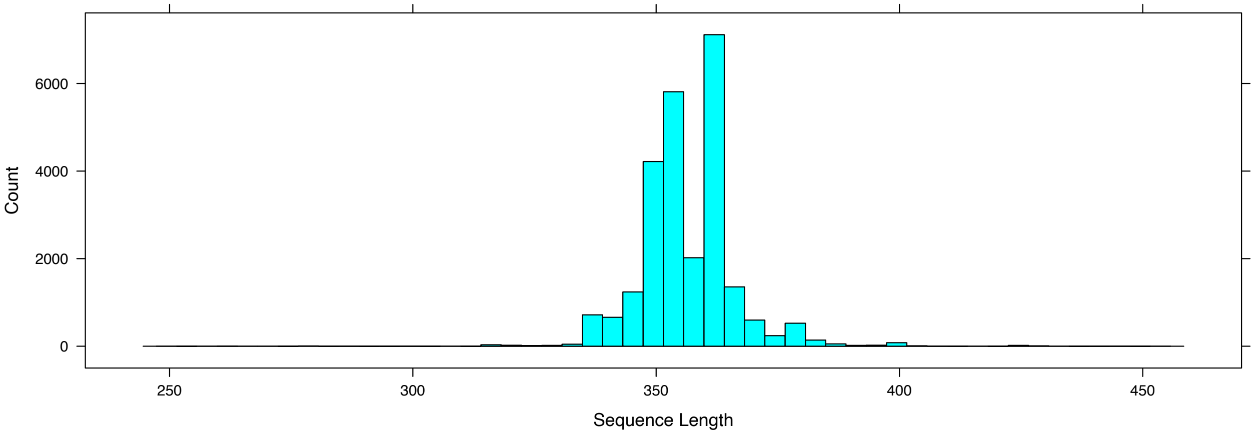


```
summary(nchar(getSequences(original_sequence_table)))
```

```
##    Min. 1st Qu.  Median    Mean 3rd Qu.    Max.
##   255.0   350.0   355.0   356.6   362.0   448.0
```

The above histogram (and summary stats), is showing the distribution of ASV sequence lengths for the merged reads. For this project, V1 and V2 was sequenced which is expected to be around ~360bp. In our case we have ASV lengths spanning from 255bp to 448bp.

Since some of these ASVs could be the result of non-specific priming, ASVs with lengths more than 20bp from the expected 360bp will be filtered out.

```
sequence_table <- original_sequence_table[,nchar(colnames(original_sequence_table)) %in% 340:380]

print(histogram(
  nchar(getSequences(sequence_table)),
  nint=50,
  type="count",
  xlab="Sequence Length"
))
```

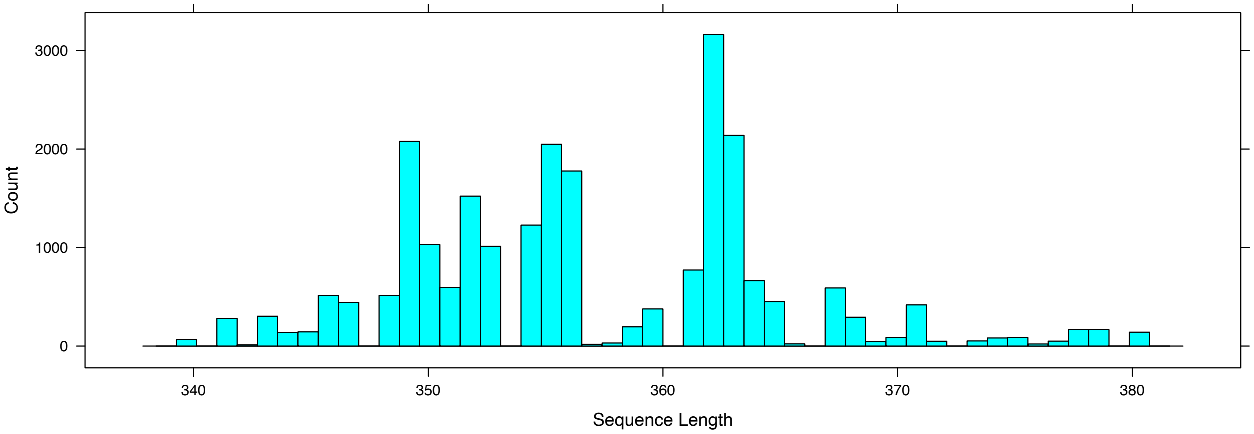


```r
summary(nchar(getSequences(sequence_table)))
```

```
##    Min. 1st Qu.  Median    Mean 3rd Qu.    Max.
##   340.0   351.0   355.0   356.9   362.0   380.0
```

After filtering ASVs based on sequence lengths, we can see in the above histogram (and summary stats) all our ASVs fall within 20bp of the expected 360bp. Before filtering there were 25025 ASVs, now after filtering there are 23784 ASVs remaining.

Remove Chimeras

```r
sequence_table_nochim <- removeBimeraDenovo(
  sequence_table, method="consensus", multithread=TRUE, verbose=TRUE
)
```

During the process of removing chimeras, it is likely for a lot of ASVs to drop out. That is ok. What is important is the abundance that these chimeras account for. In our case, 9613 ASVs, or 40.417928%, remain after identifying chimeras. These remaining ASVs account for 95.7904145% of the merged reads and the ASVs marked as chimeras accounted for 4.2095855 of the merged reads.

If you find that a majority of the merged reads were filtered as chimeras, it may be necessary to revisit the parameters used with `filterAndTrim`.

Track Reads

The following table will give an idea at which steps reads were lost throughout the analysis. Reviewing this table might be helpful to determine if it is necessary to revisit parameters used during the analysis.

```r
track <- as.data.frame(cbind(
  out,
  sapply(dada_r1, getN),
  sapply(dada_r2, getN),
  sapply(merged_reads, getN),
  rowSums(sequence_table_nochim)
))
colnames(track) <- c("input", "filtered", "denoisedR1", "denoisedR2", "merged", "nonchim")
```

```r
rownames(track) <- sample_names
track$percent_filtered_out <- 1 - (track$filtered / track$input)
track$percent_merged <- track$merged / track$filtered
track$percent_nochim <- track$nonchim / track$merged
print(track)
```

```
##                input filtered denoisedR1 denoisedR2 merged nonchim
## AAIP0060      247847   155660     153810     154071 109639  102150
## AAIP0064      196609    17039      16701      16648  13460   11563
## AAIP0093      218572   102759     102384     102243  72766   69665
## AAIP0094      189536   119207     118594     118631  98417   90301
## AAIP0119      208551   131971     130578     130753 106655   97371
## AAIP0125      460087   279408     277133     277521 198688  143910
## AAIP48        232049   164982     164120     163981 130317  120207
## ACIP0080      276447   197292     196166     195497 165687  156761
## ACIP0082      215189   133398     132279     132083 110070  104302
## ACIP0093      233884   155264     154072     154162 128154  124615
## ACIP0107      216024   140748     139545     139298 110217  106965
## ACIP0112      232745   162560     161449     160948 110491   98702
## ACIP0123      226376   159666     157911     158241 127589  117428
## ATCCCONTROL   228612   152738     152592     152191  91988   64073
## BAIP0040      191573   148259     147869     147544  99314   76890
## BLANK1          9216     5357       5262       5223   3564    3379
## BLANK2         11678     7217       7147       7070   5205    5092
## BLANK3          9286     5445       5339       5306   3170    2980
## BLANK4         15573     8575       8448       8434   5190    5034
## BLANK5         22601    12788      12689      12600   7915    7569
## BLANK6          7363     4334       4256       4190   3251    3201
## BLANK7         12662     6291       6196       6159   4318    4153
## BLANK8          7733     4122       4067       4003   3202    3038
## EAIP0027      214879   115953     114585     114881  82363   80763
## EAIP0028      241822   148092     146297     146633 107689  102159
## EAIP0029      255635   182454     180959     181217 122664   53978
## EAIP0032       14378     7437       7354       7149   4381    4349
## EAIP0067      287613   202524     200265     200683 148260  137157
## EAIP0074      294855   199124     198093     197772 142612  133992
## EAIP0083      240451   158487     155928     157086 103608   97396
## EAIP0094      260911   175309     173517     173613 113952  108057
## EAIP0104      166735   115618     114302     113372  86141   76444
## EAIP0118      198808   138525     137670     137697  85113   79293
## EAIP0122      194380   133484     131726     132349  92027   87054
## EAIP0126      207776   158220     156803     156341 116347  110044
## EAIP0128      209518   149480     147578     147457 107294  101796
## EAIP0131      220426   157621     155602     155347 111798  102589
## EAIP0135      140652    90733      89085      89342  61052   57551
## EAIP0202      292067   196379     193728     194204 129923  122485
## EAIP0208      254576   189327     188171     188153 162754  110000
## EAIP0247      221110   159060     157824     157681 117497   91611
## EAIP0285      197466   132942     131239     131123 101150   98540
## EAIP0289      196788   131743     130600     130289  91745   89988
## EAIP0298      215891   145789     143739     142706 110643  103655
## EAIP0325      204622   133436     131860     131284  87070   65974
## EAIP0339       19267    13202      13058      13089  10872   10776
```

```
## EAIP0348      218802   141465      139354      140155 111270   108739
## EAIP0373      155624   113745      112761      113023  79641    73644
## EAIP0374      154184    98910       97675       97489  49793    39043
## EAIP0405      229932   149866      148657      124412  59231    56172
## EAIP0426      261839   169408      167199      167589  98435    92586
## EAIP0429      219802   137921      137026      137303 100108    87328
## EAOP0011      185668   121228      119670      119895  99944    92536
## EAOP0018      154736   108650      107111      107407  77432    71522
## EAOP0021      135867    88758       87097       87862  62260    60176
## EAOP0046      188247   139977      139259      138533 100206    45206
## EAOP0051      258537   181547      179948      180078 143172   135696
## EAOP0055      239297   157745      156276      156714 114946   105200
## EAOP0058      195026   119525      118077      118274  83754    76723
## EAOP0066      240790   175188      173493      174094  94330    62523
## EAOP0073      193225   123971      122076      122602  96348    91002
## EAOP0074      174639   110005      108622      108993  64145    57304
## EAOP0083      185616   109315      107108      108000  77851    69612
## EAOP0086      192275   127193      125659      126292  80412    77633
## EAOP0088      268383   191249      190115      189556 132201   120293
## EAOP0090      152540   108214      107455      107492  74264    69409
## EAOP0201      140249    83023       82001       81399  51370    48274
## EAOP0203      187934   128757      127114      127373  98478    92039
## EAOP0205      171811   121813      120439      120918  93877    87779
## EAOP0216      162339   112792      111288      111602  69447    64768
## EAOP0218      271808   186013      184013      184785 139444   128350
## EAOP0236      287741   180985      179085      179854 130006    83174
## EAOP0237      193939   132051      131116      131038 111634   106212
## EAOP0241      176624   125121      122919      123430 105969   101603
## EAOP0245      386132   244462      242287      242841 174072   163851
## EAOP0249      293101   211721      209960      210193 155489   146387
## EAOP0252      284235   183545      181966      182237 146455   131296
## EAOP0258      338791   213749      211669      211250 175840   167547
## EAOP0260      273165   183542      182066      181683 149465   142913
## EAOP0276      270380   173196      171019      171507 130916   126903
## ECIP0007      275286   149162      146509      147377 109177   103212
## ECIP0253      209015   152144      151243      151318 121764   114676
## ECIP0256      258678   150804      149301      149593 119697   114599
## ECIP0257      264601   137051      135943      135747 109952   107003
## ECIP0262      272280   186536      185219      185374 166222   156837
## ECIP0264      295003   191379      189776      189657 153340   116950
## ECOP0003      313847   205827      204228      204141 165128   156813
## ECOP0011      384927   179039      177459      177862 132842   127084
## ECOP0026      381644   230864      229037      228949 190988   157047
## ECOP0032      355803   236965      234947      235061 180327   163875
## ECOP0208      248118   155508      154247      154469 109114    94880
## ECOP0223      177901   102747      101845      101656  68044    57338
## ECOP0231      144839    88543       87626       87685  67893    66244
## ECOP0236      149736    93914       93107       92869  73835    70069
## LAIP0004      145947    79109       78610       78151  63703    49783
## LAIP0005      149541   116071      115910      115537  94746    91432
## LAIP0006      206852   149965      149236      149012 117341   109164
## LAIP0007      250777   123132      122128      122529  82781    82000
## LAIP0018      231767   123013      121498      121785  97570    95493
## LAIP0037      179843   103398      102610      102690  89221    83931
```

```
## LAIP0067     102548    63005      62425      62200  53294   52344
## LCIP0011     176291   130687     130297     130283 110852  103248
## LCIP0013     298525   144575     143446     143657 102543   99883
## LCIP0045     162647    64724      63627      64220  47494   45518
## LCIP0051     274359   159287     157551     157646 115088  105922
## LCIP0059     240693   153837     152354     151248 109213   90891
## LCIP0066     265862   157789     156287     156688 130508  126255
## LCIP0071     227842   146559     145571     145578 115773  114265
## LCIP0076     193939    94509      93690      93632  77112   68748
## LCIP0085     337934   236042     235294     234584 189097  182798
## zEPTOCONTROL 221327   146723     145934     146158 106801   93990
##              percent_filtered_out percent_merged percent_nochim
## AAIP0060                0.3719512      0.7043492      0.9316940
## AAIP0064                0.9133356      0.7899525      0.8590639
## AAIP0093                0.5298620      0.7081229      0.9573839
## AAIP0094                0.3710588      0.8255975      0.9175346
## AAIP0119                0.3672003      0.8081700      0.9129530
## AAIP0125                0.3927062      0.7111035      0.7243014
## AAIP48                  0.2890209      0.7898862      0.9224199
## ACIP0080                0.2863297      0.8398060      0.9461273
## ACIP0082                0.3800891      0.8251248      0.9475970
## ACIP0093                0.3361495      0.8253942      0.9723848
## ACIP0107                0.3484613      0.7830804      0.9704946
## ACIP0112                0.3015532      0.6796937      0.8933035
## ACIP0123                0.2946867      0.7990994      0.9203615
## ATCCCONTROL             0.3318898      0.6022601      0.6965365
## BAIP0040                0.2260966      0.6698683      0.7742111
## BLANK1                  0.4187283      0.6652977      0.9480920
## BLANK2                  0.3820003      0.7212138      0.9782901
## BLANK3                  0.4136334      0.5821855      0.9400631
## BLANK4                  0.4493675      0.6052478      0.9699422
## BLANK5                  0.4341843      0.6189396      0.9562855
## BLANK6                  0.4113812      0.7501154      0.9846201
## BLANK7                  0.5031591      0.6863774      0.9617879
## BLANK8                  0.4669598      0.7768074      0.9487820
## EAIP0027                0.4603800      0.7103137      0.9805738
## EAIP0028                0.3875991      0.7271763      0.9486484
## EAIP0029                0.2862714      0.6723010      0.4400476
## EAIP0032                0.4827514      0.5890816      0.9926957
## EAIP0067                0.2958455      0.7320614      0.9251113
## EAIP0074                0.3246714      0.7161969      0.9395563
## EAIP0083                0.3408761      0.6537319      0.9400432
## EAIP0094                0.3280889      0.6500066      0.9482677
## EAIP0104                0.3065763      0.7450483      0.8874288
## EAIP0118                0.3032222      0.6144234      0.9316203
## EAIP0122                0.3132833      0.6894235      0.9459615
## EAIP0126                0.2385069      0.7353495      0.9458258
## EAIP0128                0.2865529      0.7177816      0.9487576
## EAIP0131                0.2849256      0.7092837      0.9176282
## EAIP0135                0.3549114      0.6728754      0.9426554
## EAIP0202                0.3276235      0.6615931      0.9427507
## EAIP0208                0.2563046      0.8596450      0.6758666
## EAIP0247                0.2806296      0.7386961      0.7796880
## EAIP0285                0.3267600      0.7608581      0.9741967
```

```
## EAIP0289                    0.3305334      0.6963937      0.9808491
## EAIP0298                    0.3247102      0.7589256      0.9368419
## EAIP0325                    0.3478903      0.6525226      0.7577122
## EAIP0339                    0.3147869      0.8235116      0.9911700
## EAIP0348                    0.3534565      0.7865550      0.9772535
## EAIP0373                    0.2691037      0.7001714      0.9246996
## EAIP0374                    0.3584937      0.5034172      0.7841062
## EAIP0405                    0.3482160      0.3952264      0.9483547
## EAIP0426                    0.3530070      0.5810528      0.9405801
## EAIP0429                    0.3725216      0.7258358      0.8723379
## EAOP0011                    0.3470711      0.8244300      0.9258785
## EAOP0018                    0.2978363      0.7126737      0.9236750
## EAOP0021                    0.3467288      0.7014579      0.9665275
## EAOP0046                    0.2564184      0.7158748      0.4511307
## EAOP0051                    0.2977910      0.7886222      0.9477831
## EAOP0055                    0.3407983      0.7286824      0.9152124
## EAOP0058                    0.3871330      0.7007237      0.9160518
## EAOP0066                    0.2724449      0.5384501      0.6628114
## EAOP0073                    0.3584112      0.7771818      0.9445136
## EAOP0074                    0.3701006      0.5831099      0.8933510
## EAOP0083                    0.4110691      0.7121712      0.8941696
## EAOP0086                    0.3384839      0.6322046      0.9654405
## EAOP0088                    0.2874027      0.6912507      0.9099250
## EAOP0090                    0.2905861      0.6862698      0.9346251
## EAOP0201                    0.4080314      0.6187442      0.9397314
## EAOP0203                    0.3148818      0.7648361      0.9346148
## EAOP0205                    0.2910058      0.7706649      0.9350427
## EAOP0216                    0.3052070      0.6157086      0.9326249
## EAOP0218                    0.3156456      0.7496465      0.9204412
## EAOP0236                    0.3710142      0.7183247      0.6397705
## EAOP0237                    0.3191106      0.8453855      0.9514306
## EAOP0241                    0.2915968      0.8469322      0.9587993
## EAOP0245                    0.3668953      0.7120616      0.9412829
## EAOP0249                    0.2776517      0.7344052      0.9414621
## EAOP0252                    0.3542491      0.7979242      0.8964938
## EAOP0258                    0.3690830      0.8226471      0.9528378
## EAOP0260                    0.3280911      0.8143368      0.9561637
## EAOP0276                    0.3594349      0.7558835      0.9693468
## ECIP0007                    0.4581562      0.7319357      0.9453640
## ECIP0253                    0.2720905      0.8003207      0.9417890
## ECIP0256                    0.4170204      0.7937256      0.9574091
## ECIP0257                    0.4820466      0.8022707      0.9731792
## ECIP0262                    0.3149111      0.8910988      0.9435394
## ECIP0264                    0.3512642      0.8012373      0.7626842
## ECOP0003                    0.3441804      0.8022660      0.9496451
## ECOP0011                    0.5348754      0.7419724      0.9566553
## ECOP0026                    0.3950802      0.8272749      0.8222873
## ECOP0032                    0.3339994      0.7609858      0.9087657
## ECOP0208                    0.3732498      0.7016617      0.8695493
## ECOP0223                    0.4224484      0.6622480      0.8426606
## ECOP0231                    0.3886798      0.7667800      0.9757118
## ECOP0236                    0.3728028      0.7861980      0.9489944
## LAIP0004                    0.4579608      0.8052560      0.7814860
## LAIP0005                    0.2238182      0.8162762      0.9650223
```

```
## LAIP0006                  0.2750131     0.7824559     0.9303142
## LAIP0007                  0.5089980     0.6722948     0.9905655
## LAIP0018                  0.4692385     0.7931682     0.9787127
## LAIP0037                  0.4250652     0.8628890     0.9407090
## LAIP0067                  0.3856048     0.8458694     0.9821744
## LCIP0011                  0.2586859     0.8482251     0.9314040
## LCIP0013                  0.5157022     0.7092720     0.9740597
## LCIP0045                  0.6020584     0.7337927     0.9583947
## LCIP0051                  0.4194213     0.7225197     0.9203566
## LCIP0059                  0.3608580     0.7099267     0.8322361
## LCIP0066                  0.4065004     0.8271046     0.9674120
## LCIP0071                  0.3567516     0.7899413     0.9869745
## LCIP0076                  0.5126870     0.8159223     0.8915344
## LCIP0085                  0.3015145     0.8011159     0.9666891
## zEPTOCONTROL              0.3370759     0.7279091     0.8800479
```

Assign Taxonomy

The SILVA (v132) DADA2-formatted reference database was used to assign taxonomy (including species) to the ASVs. The SILVA (v132) DADA2 formatted databases are available at DADA2 Reference Databases.

```
taxa <- assignTaxonomy(
  sequence_table_nochim,
  paste0(PROJECT_DIR, "/data/reference/silva_nr_v132_train_set.fa.gz"),
  tryRC=TRUE,
  multithread=TRUE
)
taxa <- addSpecies(
  taxa,
  paste0(PROJECT_DIR, "/data/reference/silva_species_assignment_v132.fa.gz")
)

asv_seqs <- colnames(sequence_table_nochim)
asv_headers <- vector(dim(sequence_table_nochim)[2], mode="character")
for (i in 1:dim(sequence_table_nochim)[2]) {
  asv_headers[i] <- paste(">ASV", i, sep="_")
}
asv_fasta <- c(rbind(asv_headers, asv_seqs))
write(asv_fasta, paste0(PROJECT_DIR, "/results/ASVs-withcontams.fa"))

asv_tab <- t(sequence_table_nochim)
row.names(asv_tab) <- sub(">", "", asv_headers)
write.table(
  asv_tab,
  paste0(PROJECT_DIR, "/results/ASVs-withcontams-counts.tsv"),
  sep="\t",
  quote=F,
  col.names=NA
)

asv_tax <- taxa
row.names(asv_tax) <- sub(">", "", asv_headers)
write.table(
```

```
  asv_tax,
  paste0(PROJECT_DIR, "/results/ASVs-withcontams-taxonomy.tsv"),
  sep="\t",
  quote=F,
  col.names=NA
)

taxa_print <- as.data.frame(taxa)
rownames(taxa_print) <- NULL
head(taxa_print)
```

```
##    Kingdom         Phylum               Class             Order
## 1 Bacteria  Bacteroidetes         Bacteroidia     Bacteroidales
## 2 Bacteria     Firmicutes          Clostridia     Clostridiales
## 3 Bacteria Proteobacteria Gammaproteobacteria Enterobacteriales
## 4 Bacteria Proteobacteria Gammaproteobacteria Enterobacteriales
## 5 Bacteria  Bacteroidetes         Bacteroidia     Bacteroidales
## 6 Bacteria     Firmicutes             Bacilli   Lactobacillales
##               Family              Genus    Species
## 1     Bacteroidaceae        Bacteroides   vulgatus
## 2    Ruminococcaceae    Subdoligranulum       <NA>
## 3 Enterobacteriaceae Pseudocitrobacter       <NA>
## 4 Enterobacteriaceae               <NA>       <NA>
## 5      Rikenellaceae          Alistipes putredinis
## 6   Lactobacillaceae      Lactobacillus       <NA>
```

Taxonomy was assigned to 9613 ASVs, of these, 375 also had a species assigned. The results of these assignments were written to the following files:

ASV Sequences (FASTA)
Each ASV sequence was written to *results/ASVs-withcontams.fa* in FASTA format

ASV Counts Per Sample
A TSV of the count for each ASV within each sample was written to *results/ASVs-withcontams-counts.tsv*

Taxonomy Assignment Per ASV
The taxonomic assignment for each ASV (similar to the table above) was written to *results/ASVs-withcontams-taxonomy.tsv*

Remove Contaminants

decontam is used to identify ASVs that are likely contaminants. decontam includes two methods to identify contaminants, frequency and prevalence. In this analysis, the prevalence method is used. This method compares the *prevalence* (presence/absence across samples) of each ASV in true positive samples against negative controls (e.g. *BLANK* samples) to identify contaminants.

```
vector_for_decontam <- grepl("BLANK", colnames(asv_tab))
contam_asvs <- isContaminant(t(asv_tab), neg=vector_for_decontam)
table(contam_asvs$contaminant)
```

```
##
## FALSE  TRUE
##  9589    24
```

```r
is_contaminant <- row.names(contam_asvs[contam_asvs$contaminant == TRUE,])
asv_tax[row.names(asv_tax) %in% is_contaminant, ]
```

```
##          Kingdom    Phylum           Class
## ASV_86   "Bacteria" "Proteobacteria" "Gammaproteobacteria"
## ASV_176  "Bacteria" "Proteobacteria" "Gammaproteobacteria"
## ASV_200  "Bacteria" "Firmicutes"     "Bacilli"
## ASV_388  "Bacteria" "Firmicutes"     "Bacilli"
## ASV_541  "Bacteria" "Actinobacteria" "Actinobacteria"
## ASV_702  "Bacteria" "Proteobacteria" "Gammaproteobacteria"
## ASV_943  "Bacteria" "Proteobacteria" "Gammaproteobacteria"
## ASV_1097 "Bacteria" "Firmicutes"     "Bacilli"
## ASV_1364 "Bacteria" "Firmicutes"     "Bacilli"
## ASV_1713 "Bacteria" "Firmicutes"     "Bacilli"
## ASV_2000 "Bacteria" "Proteobacteria" "Gammaproteobacteria"
## ASV_2036 "Bacteria" "Proteobacteria" "Gammaproteobacteria"
## ASV_2441 "Bacteria" "Actinobacteria" "Actinobacteria"
## ASV_2624 "Bacteria" "Actinobacteria" "Actinobacteria"
## ASV_2695 "Bacteria" "Firmicutes"     "Bacilli"
## ASV_2735 "Bacteria" "Firmicutes"     "Bacilli"
## ASV_2768 "Bacteria" "Proteobacteria" "Gammaproteobacteria"
## ASV_3246 "Bacteria" "Proteobacteria" "Gammaproteobacteria"
## ASV_3859 "Bacteria" "Firmicutes"     "Bacilli"
## ASV_3898 "Bacteria" "Firmicutes"     "Bacilli"
## ASV_4253 "Bacteria" "Firmicutes"     "Bacilli"
## ASV_4441 "Bacteria" "Firmicutes"     "Bacilli"
## ASV_4442 "Bacteria" "Proteobacteria" "Gammaproteobacteria"
## ASV_5226 "Bacteria" "Proteobacteria" "Gammaproteobacteria"
##          Order                     Family
## ASV_86   "Betaproteobacteriales" "Burkholderiaceae"
## ASV_176  "Betaproteobacteriales" "Burkholderiaceae"
## ASV_200  "Bacillales"            "Bacillaceae"
## ASV_388  "Bacillales"            "Bacillaceae"
## ASV_541  "Micrococcales"         "Microbacteriaceae"
## ASV_702  "Betaproteobacteriales" "Burkholderiaceae"
## ASV_943  "Xanthomonadales"       "Xanthomonadaceae"
## ASV_1097 "Bacillales"            "Bacillaceae"
## ASV_1364 "Bacillales"            "Bacillaceae"
## ASV_1713 "Bacillales"            "Bacillaceae"
## ASV_2000 "Betaproteobacteriales" "Burkholderiaceae"
## ASV_2036 "Betaproteobacteriales" "Burkholderiaceae"
## ASV_2441 "Propionibacteriales"   "Propionibacteriaceae"
## ASV_2624 "Corynebacteriales"     "Nocardiaceae"
## ASV_2695 "Bacillales"            "Bacillaceae"
## ASV_2735 "Bacillales"            "Bacillaceae"
## ASV_2768 "Betaproteobacteriales" "Burkholderiaceae"
## ASV_3246 "Betaproteobacteriales" "Burkholderiaceae"
## ASV_3859 "Bacillales"            "Bacillaceae"
## ASV_3898 "Bacillales"            NA
## ASV_4253 "Bacillales"            "Bacillaceae"
## ASV_4441 "Bacillales"            "Bacillaceae"
## ASV_4442 "Oceanospirillales"     "Halomonadaceae"
## ASV_5226 "Pseudomonadales"       "Pseudomonadaceae"
```

```
##           Genus                                          Species
## ASV_86   "Delftia"                                      NA
## ASV_176  "Achromobacter"                                NA
## ASV_200  "Anaerobacillus"                               NA
## ASV_388  "Anaerobacillus"                               NA
## ASV_541  "Microbacterium"                               NA
## ASV_702  "Ralstonia"                                    NA
## ASV_943  "Stenotrophomonas"                             "maltophilia"
## ASV_1097 "Anaerobacillus"                               NA
## ASV_1364 "Anaerobacillus"                               NA
## ASV_1713 "Anaerobacillus"                               NA
## ASV_2000 "Herbaspirillum"                               "huttiense"
## ASV_2036 "Acidovorax"                                   NA
## ASV_2441 "Cutibacterium"                                NA
## ASV_2624 "Rhodococcus"                                  NA
## ASV_2695 NA                                             NA
## ASV_2735 "Bacillus"                                     "pseudofirmus"
## ASV_2768 "Herbaspirillum"                               NA
## ASV_3246 "Burkholderia-Caballeronia-Paraburkholderia" NA
## ASV_3859 "Anaerobacillus"                               NA
## ASV_3898 NA                                             NA
## ASV_4253 NA                                             NA
## ASV_4441 NA                                             NA
## ASV_4442 "Halomonas"                                    NA
## ASV_5226 "Pseudomonas"                                  NA
```

```r
contam_indices <- which(asv_fasta %in% paste0(">", is_contaminant))
dont_want <- sort(c(contam_indices, contam_indices + 1))

asv_fasta_no_contam <- asv_fasta[- dont_want]
write(asv_fasta_no_contam, paste0(PROJECT_DIR, "/results/ASVs-decontam.fa"))

asv_tab_no_contam <- asv_tab[!row.names(asv_tab) %in% is_contaminant, ]
write.table(
  asv_tab_no_contam,
  paste0(PROJECT_DIR, "/results/ASVs-decontam-counts.tsv"),
  sep="\t",
  quote=F,
  col.names=NA
)

asv_tax_no_contam <- asv_tax[!row.names(asv_tax) %in% is_contaminant, ]
write.table(
  asv_tax_no_contam,
  paste0(PROJECT_DIR, "/results/ASVs-decontam-taxonomy.tsv"),
  sep="\t",
  quote=F,
  col.names=NA
)
```

24 ASVs were identified as contaminants and their taxonmic assignments were filtered out. The decontaminated results were written to the following files:

ASV Sequences (FASTA)
Each decontaminated ASV sequence was written to *results/ASVs-decontam.fa* in FASTA format

ASV Counts Per Sample
A TSV of the count for each decontaminated ASV within each sample was written to *results/ASVs-decontam-counts.tsv*

Taxonomy Assignment Per ASV
The taxonomic assignment for each decontaminated ASV (similar to the table above) was written to *results/ASVs-decontam-taxonomy.tsv*

Plot Sample FASTQ Quality Profiles (Full Set)

```
for (i in 1:length(fastq_r1)) {
  print(plotQualityProfile(c(fastq_r1[i], fastq_r2[i])))
}
```

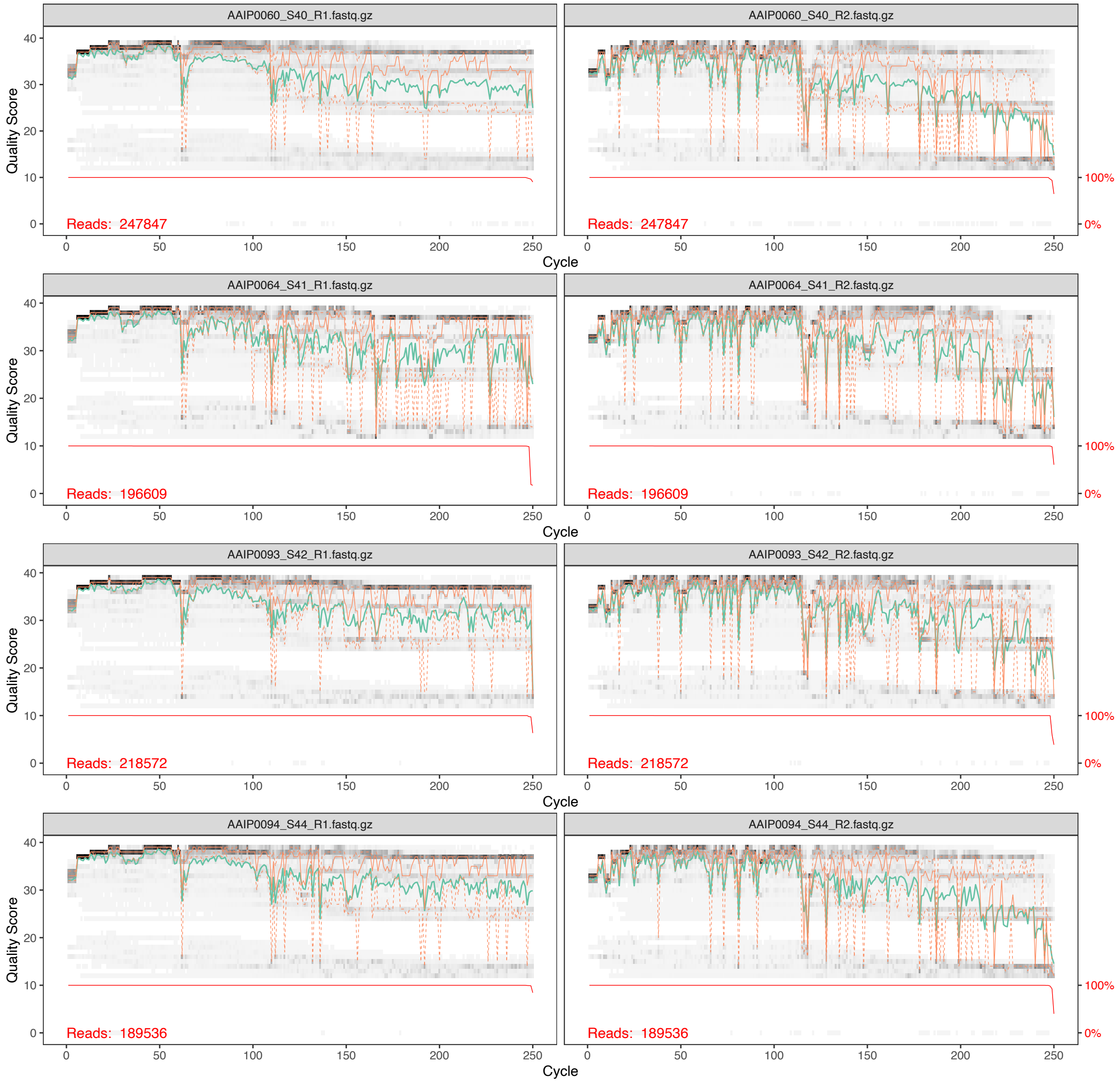

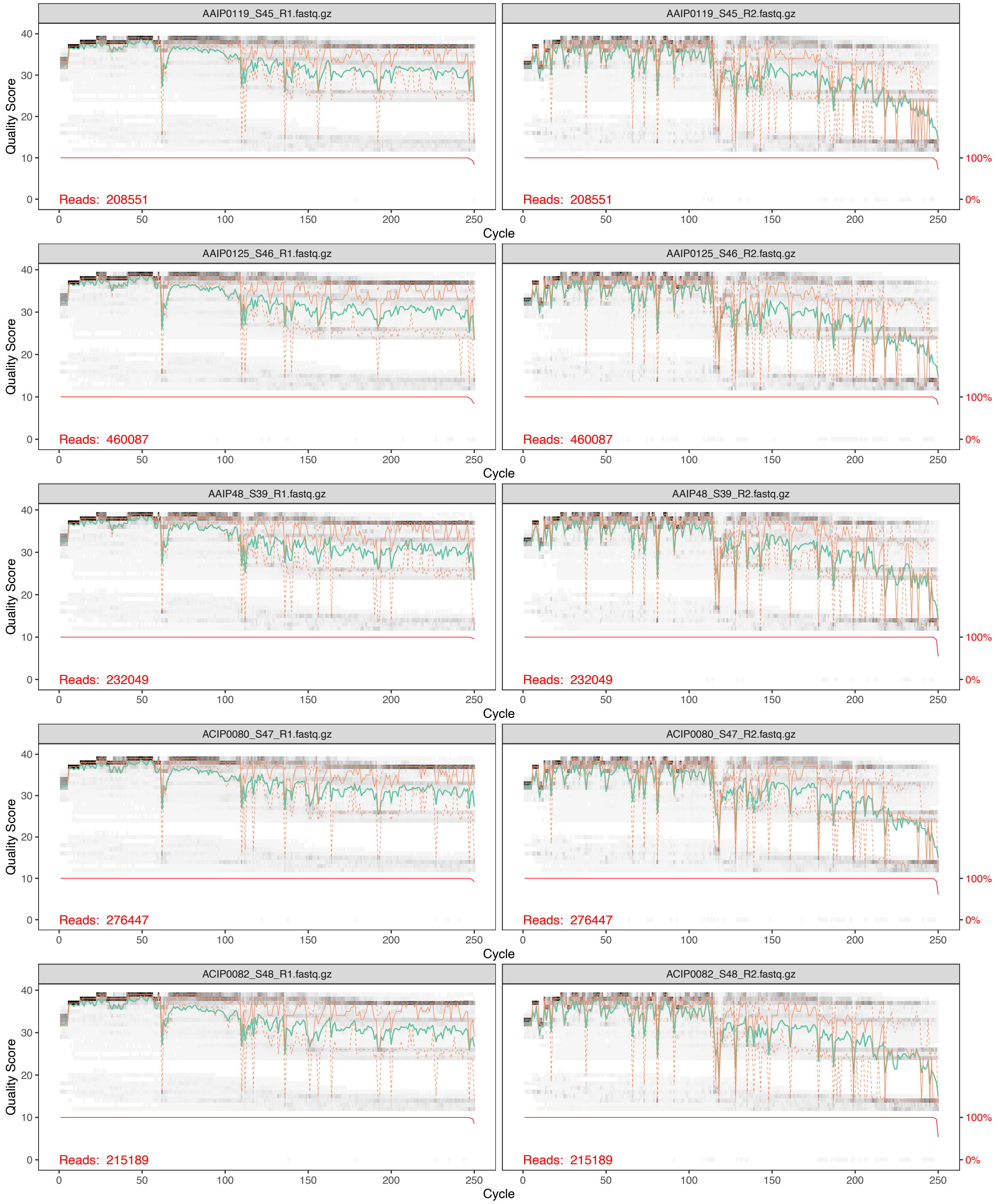

AAIP0119_S45_R1.fastq.gz
AAIP0119_S45_R2.fastq.gz
Reads: 208551
Reads: 208551
AAIP0125_S46_R1.fastq.gz
AAIP0125_S46_R2.fastq.gz
Reads: 460087
Reads: 460087
AAIP48_S39_R1.fastq.gz
AAIP48_S39_R2.fastq.gz
Reads: 232049
Reads: 232049
ACIP0080_S47_R1.fastq.gz
ACIP0080_S47_R2.fastq.gz
Reads: 276447
Reads: 276447
ACIP0082_S48_R1.fastq.gz
ACIP0082_S48_R2.fastq.gz
Reads: 215189
Reads: 215189
Quality Score
Cycle
100%
0%

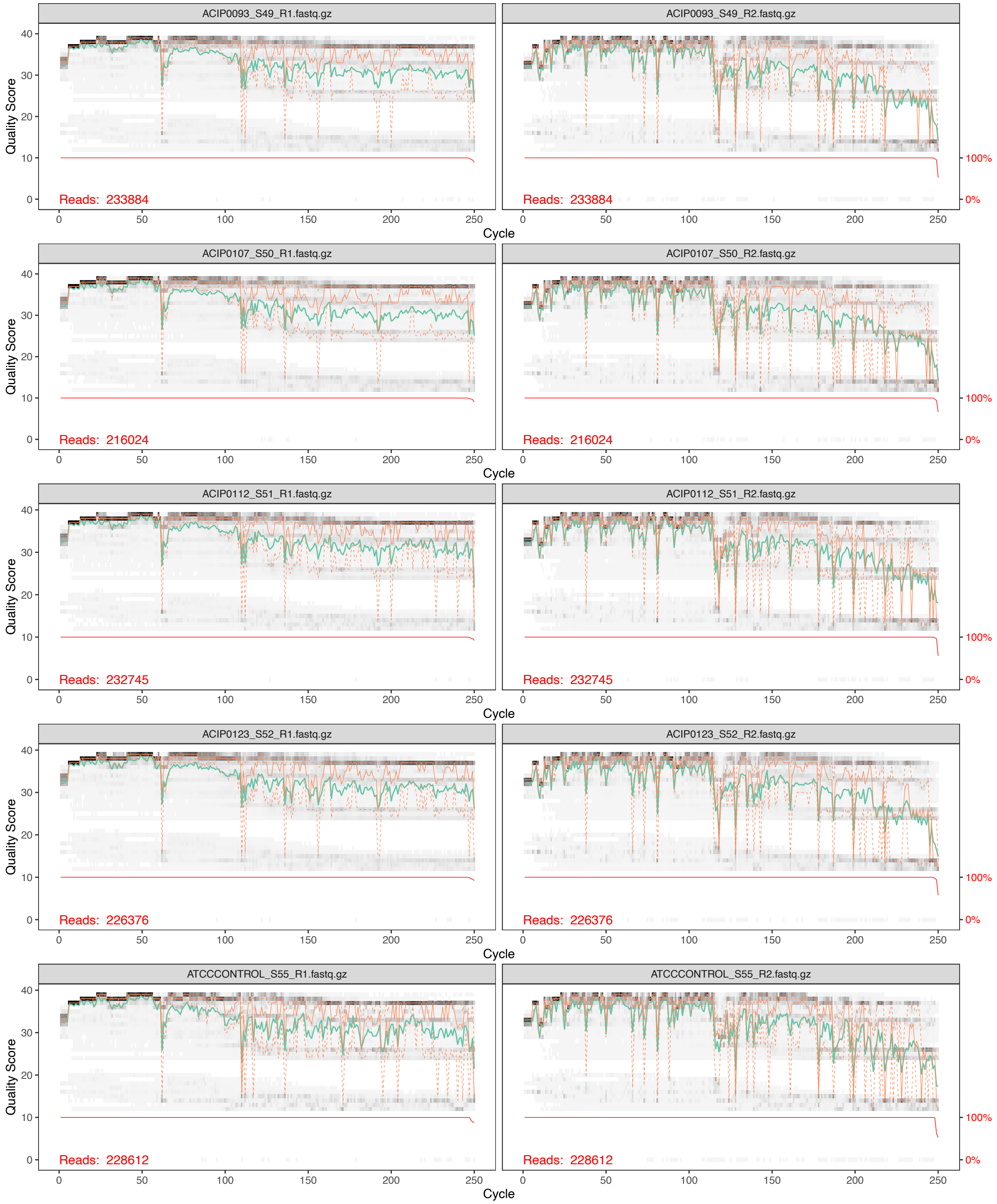
ACIP0093_S49_R1.fastq.gz
ACIP0093_S49_R2.fastq.gz
Reads: 233884
Reads: 233884
ACIP0107_S50_R1.fastq.gz
ACIP0107_S50_R2.fastq.gz
Reads: 216024
Reads: 216024
ACIP0112_S51_R1.fastq.gz
ACIP0112_S51_R2.fastq.gz
Reads: 232745
Reads: 232745
ACIP0123_S52_R1.fastq.gz
ACIP0123_S52_R2.fastq.gz
Reads: 226376
Reads: 226376
ATCCCONTROL_S55_R1.fastq.gz
ATCCCONTROL_S55_R2.fastq.gz
Reads: 228612
Reads: 228612
Quality Score
Cycle
100%
0%

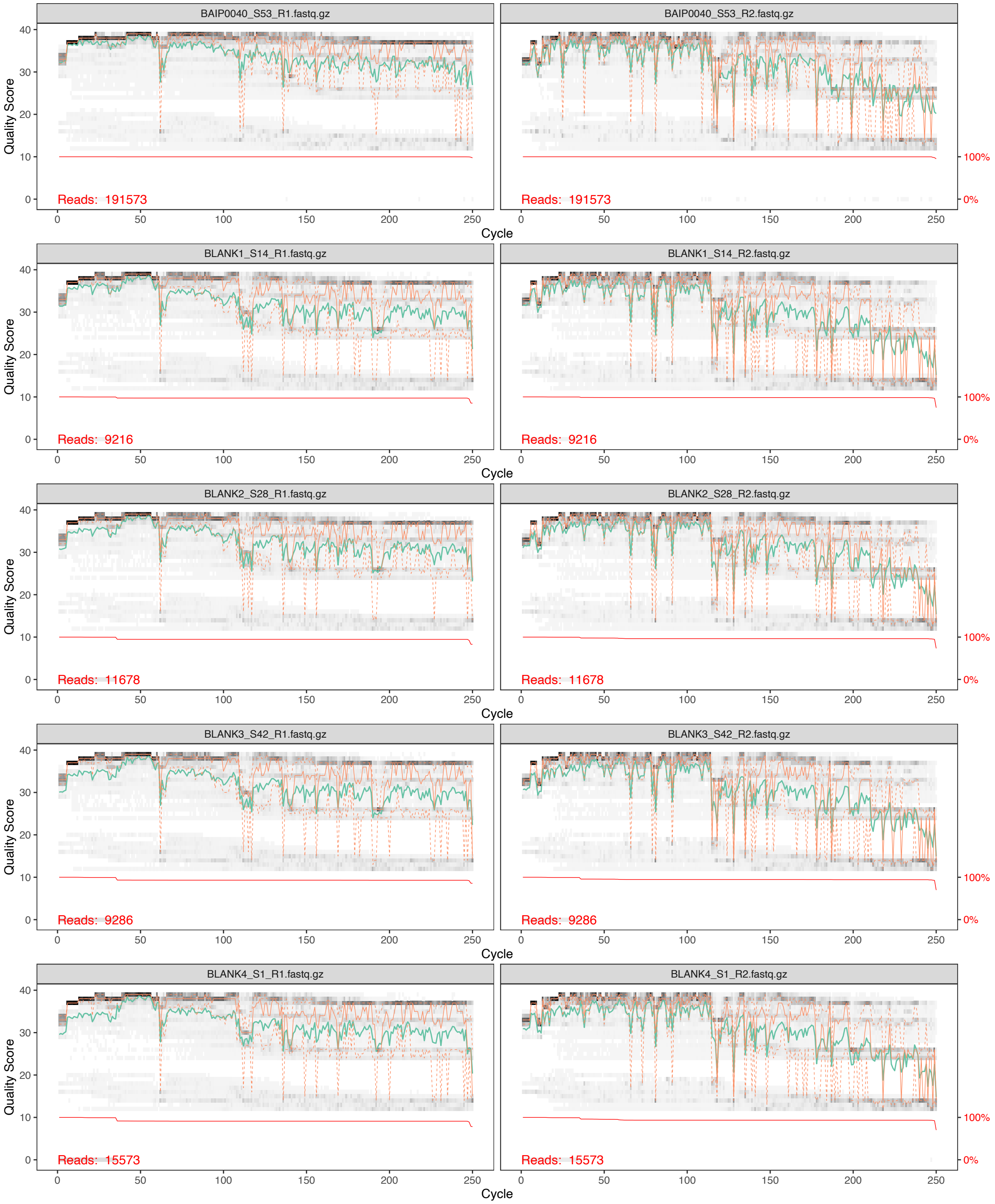
BAIP0040_S53_R1.fastq.gz
BAIP0040_S53_R2.fastq.gz
Reads: 191573
Reads: 191573
BLANK1_S14_R1.fastq.gz
BLANK1_S14_R2.fastq.gz
Reads: 9216
Reads: 9216
BLANK2_S28_R1.fastq.gz
BLANK2_S28_R2.fastq.gz
Reads: 11678
Reads: 11678
BLANK3_S42_R1.fastq.gz
BLANK3_S42_R2.fastq.gz
Reads: 9286
Reads: 9286
BLANK4_S1_R1.fastq.gz
BLANK4_S1_R2.fastq.gz
Reads: 15573
Reads: 15573
Quality Score
Cycle
100%
0%

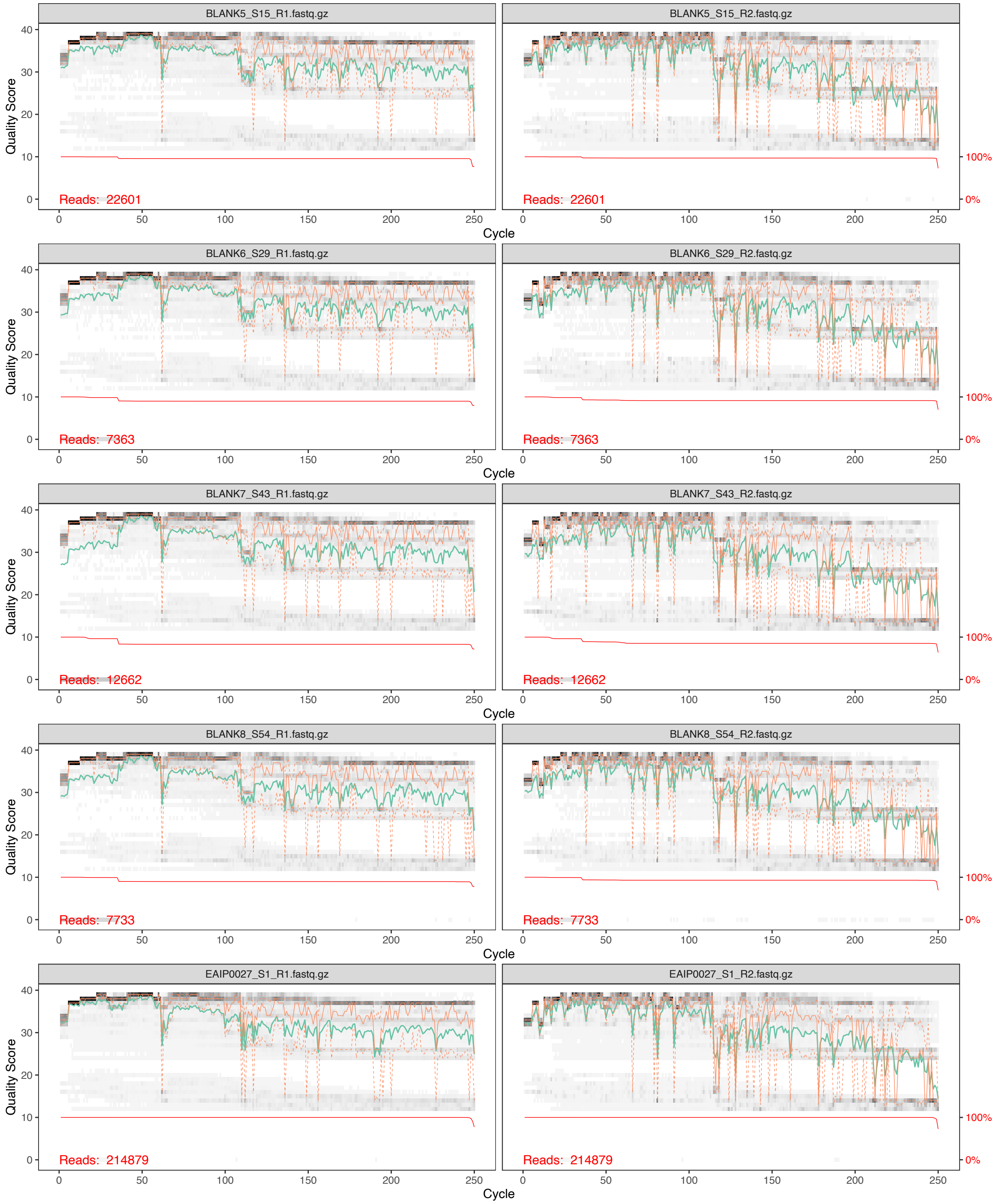

BLANK5_S15_R1.fastq.gz
BLANK5_S15_R2.fastq.gz
Reads: 22601
Reads: 22601
BLANK6_S29_R1.fastq.gz
BLANK6_S29_R2.fastq.gz
Reads: 7363
Reads: 7363
BLANK7_S43_R1.fastq.gz
BLANK7_S43_R2.fastq.gz
Reads: 12662
Reads: 12662
BLANK8_S54_R1.fastq.gz
BLANK8_S54_R2.fastq.gz
Reads: 7733
Reads: 7733
EAIP0027_S1_R1.fastq.gz
EAIP0027_S1_R2.fastq.gz
Reads: 214879
Reads: 214879
Quality Score
Cycle
100%
0%

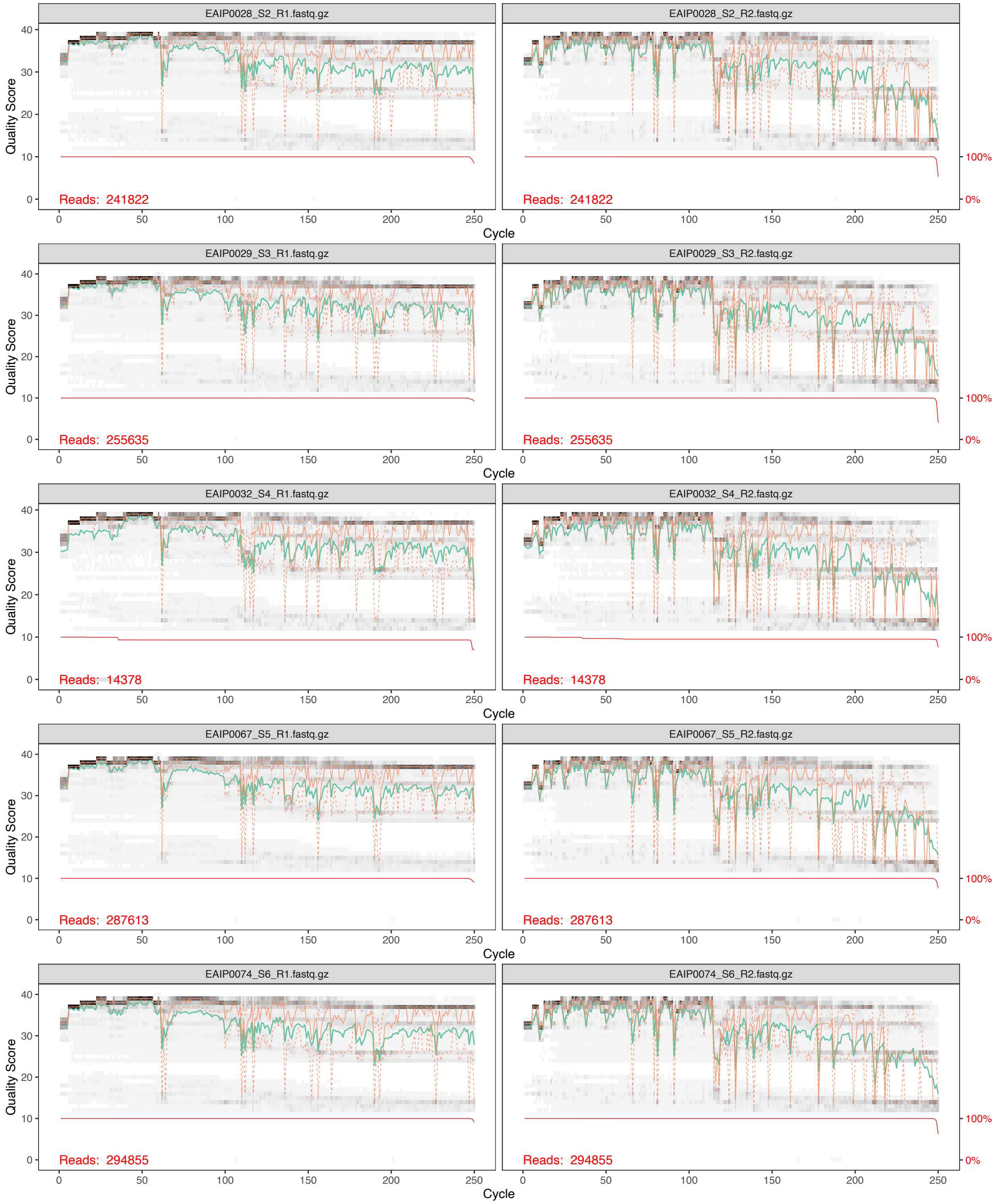
EAIP0028_S2_R1.fastq.gz
EAIP0028_S2_R2.fastq.gz
Reads: 241822
Reads: 241822
EAIP0029_S3_R1.fastq.gz
EAIP0029_S3_R2.fastq.gz
Reads: 255635
Reads: 255635
EAIP0032_S4_R1.fastq.gz
EAIP0032_S4_R2.fastq.gz
Reads: 14378
Reads: 14378
EAIP0067_S5_R1.fastq.gz
EAIP0067_S5_R2.fastq.gz
Reads: 287613
Reads: 287613
EAIP0074_S6_R1.fastq.gz
EAIP0074_S6_R2.fastq.gz
Reads: 294855
Reads: 294855
Quality Score
Cycle
100%
0%

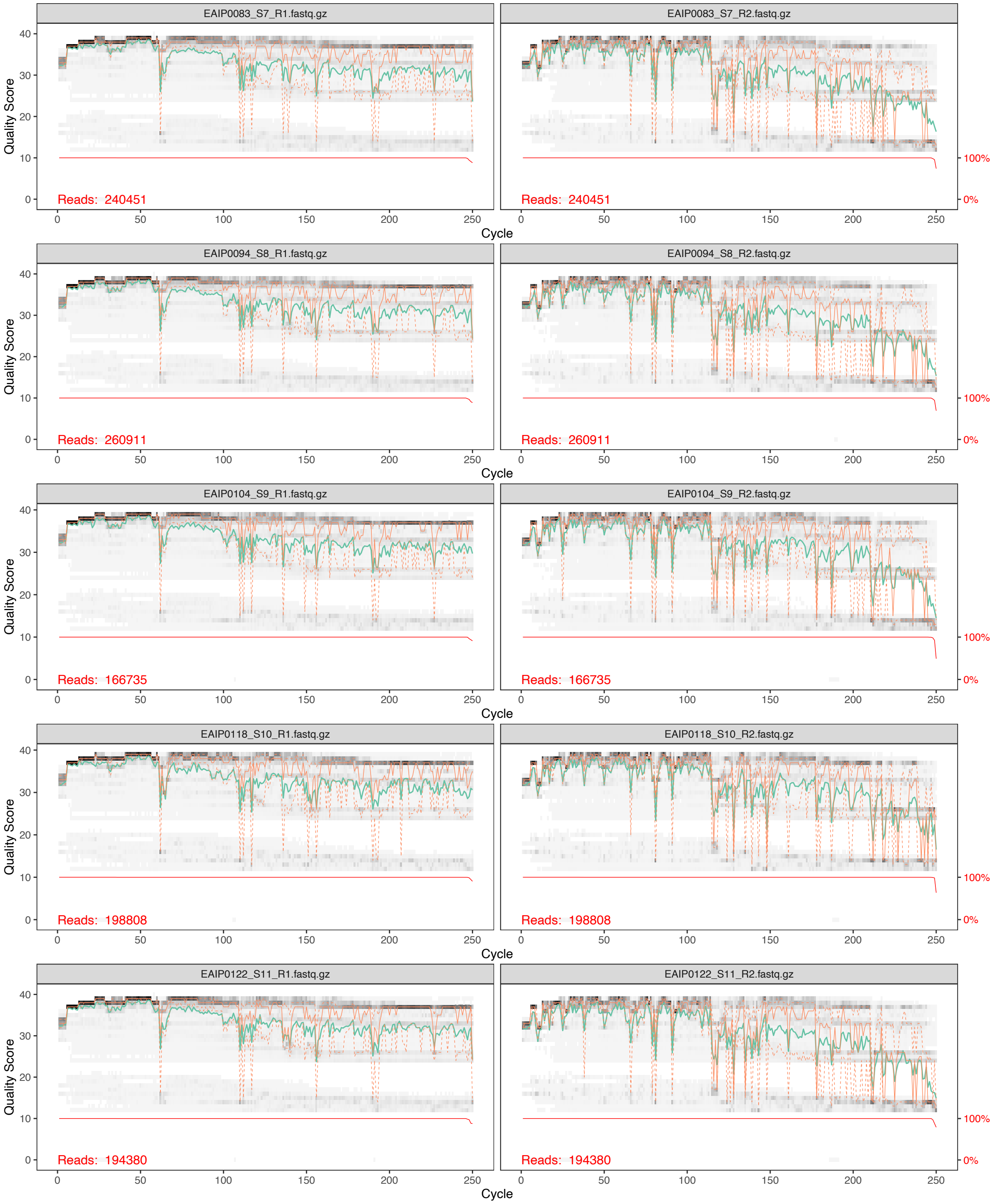
EAIP0083_S7_R1.fastq.gz
EAIP0083_S7_R2.fastq.gz
Reads: 240451
Reads: 240451
EAIP0094_S8_R1.fastq.gz
EAIP0094_S8_R2.fastq.gz
Reads: 260911
Reads: 260911
EAIP0104_S9_R1.fastq.gz
EAIP0104_S9_R2.fastq.gz
Reads: 166735
Reads: 166735
EAIP0118_S10_R1.fastq.gz
EAIP0118_S10_R2.fastq.gz
Reads: 198808
Reads: 198808
EAIP0122_S11_R1.fastq.gz
EAIP0122_S11_R2.fastq.gz
Reads: 194380
Reads: 194380
Quality Score
Cycle
100%
0%

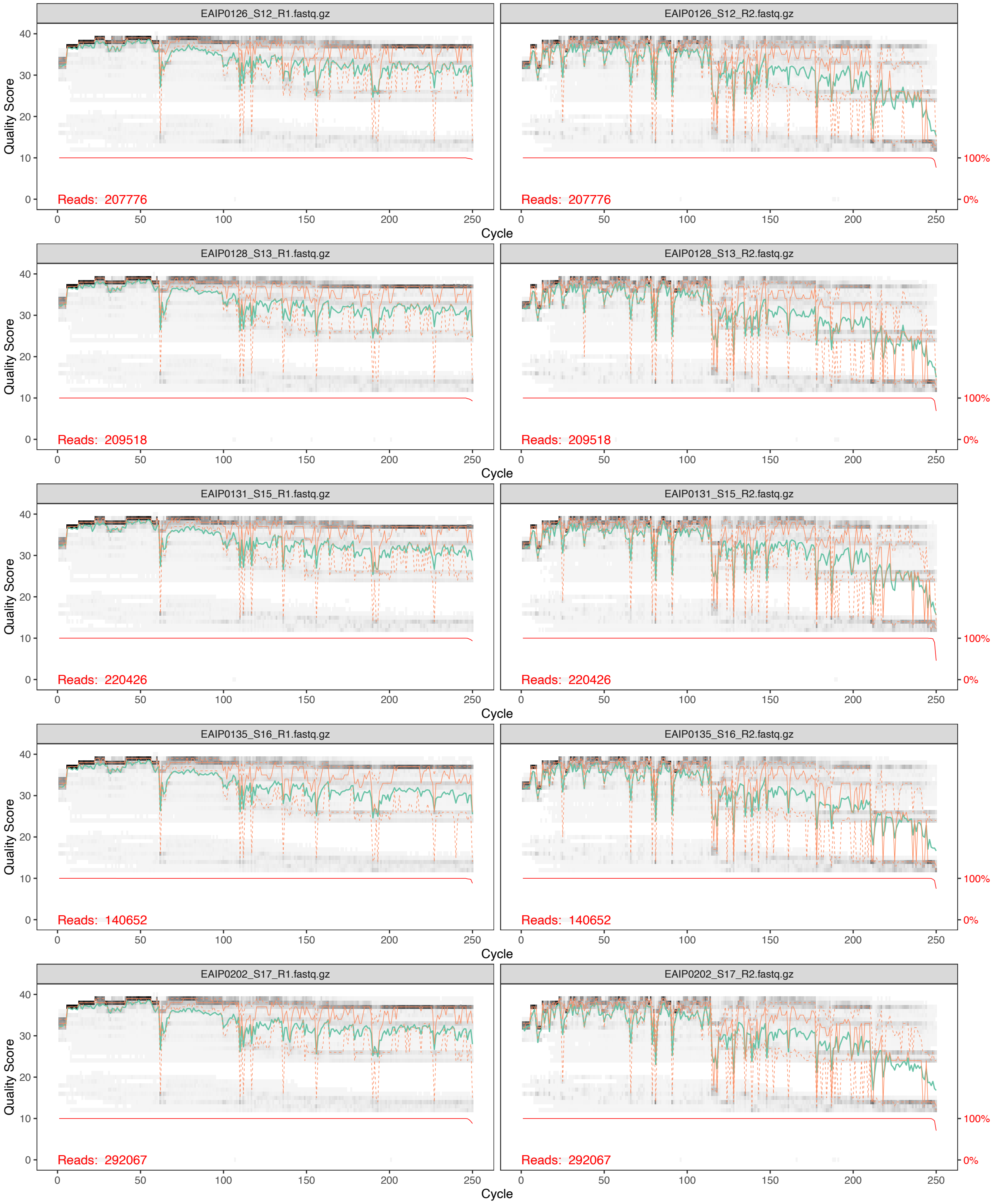
EAIP0126_S12_R1.fastq.gz
EAIP0126_S12_R2.fastq.gz
Reads: 207776
Reads: 207776
EAIP0128_S13_R1.fastq.gz
EAIP0128_S13_R2.fastq.gz
Reads: 209518
Reads: 209518
EAIP0131_S15_R1.fastq.gz
EAIP0131_S15_R2.fastq.gz
Reads: 220426
Reads: 220426
EAIP0135_S16_R1.fastq.gz
EAIP0135_S16_R2.fastq.gz
Reads: 140652
Reads: 140652
EAIP0202_S17_R1.fastq.gz
EAIP0202_S17_R2.fastq.gz
Reads: 292067
Reads: 292067
Quality Score
Cycle
100%
0%

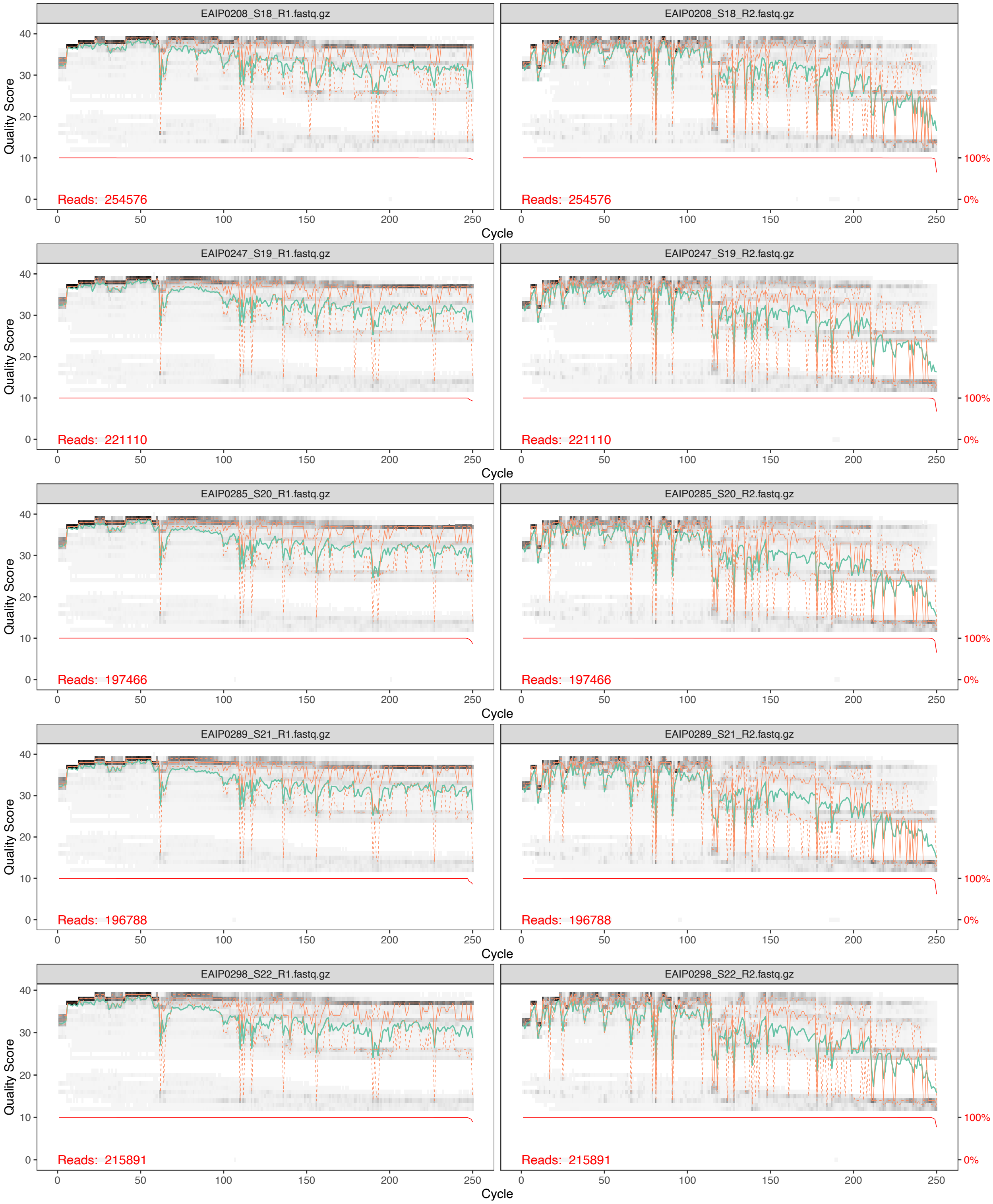
EAIP0208_S18_R1.fastq.gz
EAIP0208_S18_R2.fastq.gz
Reads: 254576
Reads: 254576
EAIP0247_S19_R1.fastq.gz
EAIP0247_S19_R2.fastq.gz
Reads: 221110
Reads: 221110
EAIP0285_S20_R1.fastq.gz
EAIP0285_S20_R2.fastq.gz
Reads: 197466
Reads: 197466
EAIP0289_S21_R1.fastq.gz
EAIP0289_S21_R2.fastq.gz
Reads: 196788
Reads: 196788
EAIP0298_S22_R1.fastq.gz
EAIP0298_S22_R2.fastq.gz
Reads: 215891
Reads: 215891
Quality Score
Cycle
100%
0%

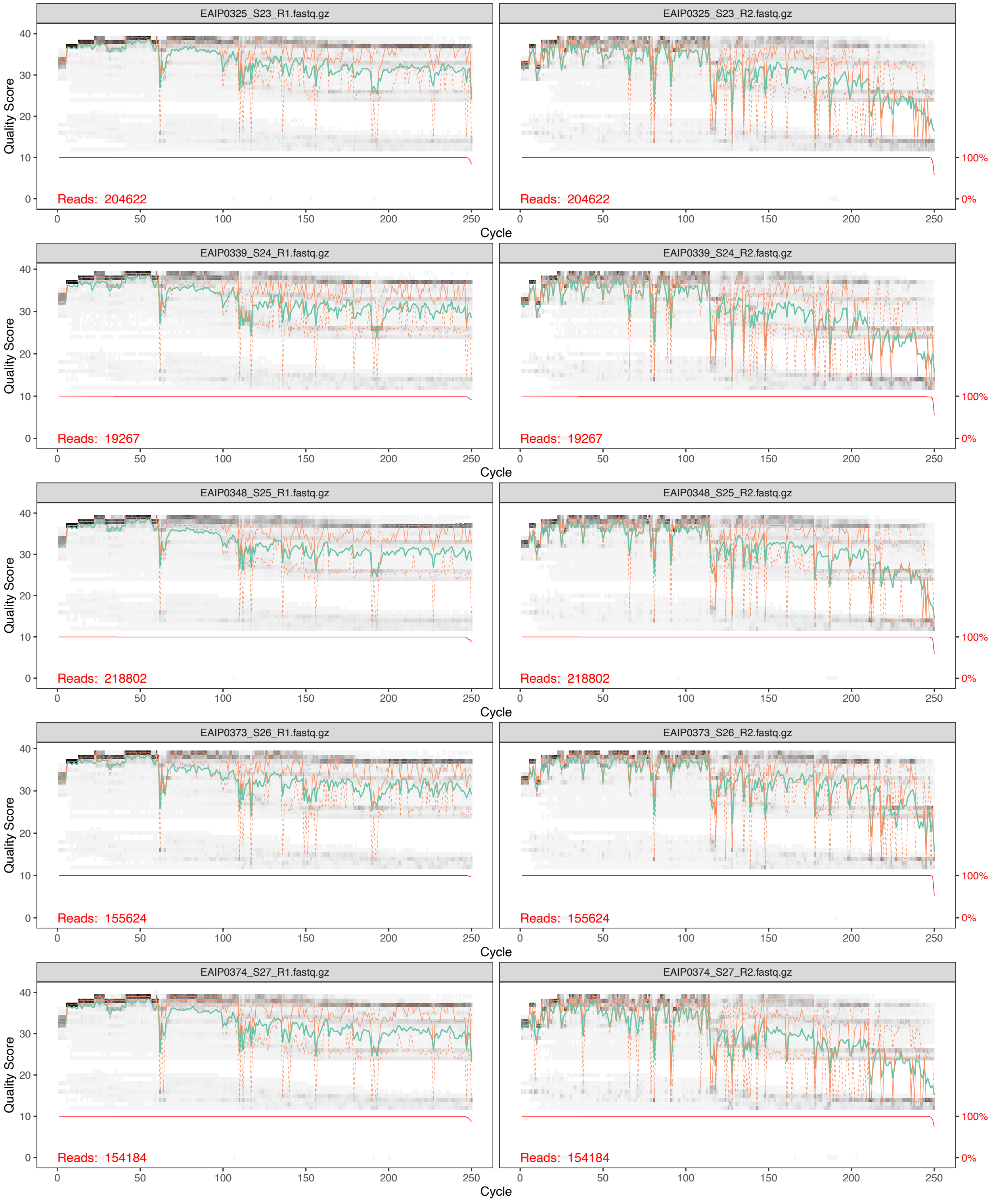
EAIP0325_S23_R1.fastq.gz
EAIP0325_S23_R2.fastq.gz
Reads: 204622
Reads: 204622
EAIP0339_S24_R1.fastq.gz
EAIP0339_S24_R2.fastq.gz
Reads: 19267
Reads: 19267
EAIP0348_S25_R1.fastq.gz
EAIP0348_S25_R2.fastq.gz
Reads: 218802
Reads: 218802
EAIP0373_S26_R1.fastq.gz
EAIP0373_S26_R2.fastq.gz
Reads: 155624
Reads: 155624
EAIP0374_S27_R1.fastq.gz
EAIP0374_S27_R2.fastq.gz
Reads: 154184
Reads: 154184
Quality Score
Cycle
0
10
20
30
40
50
100
150
200
250
100%
0%

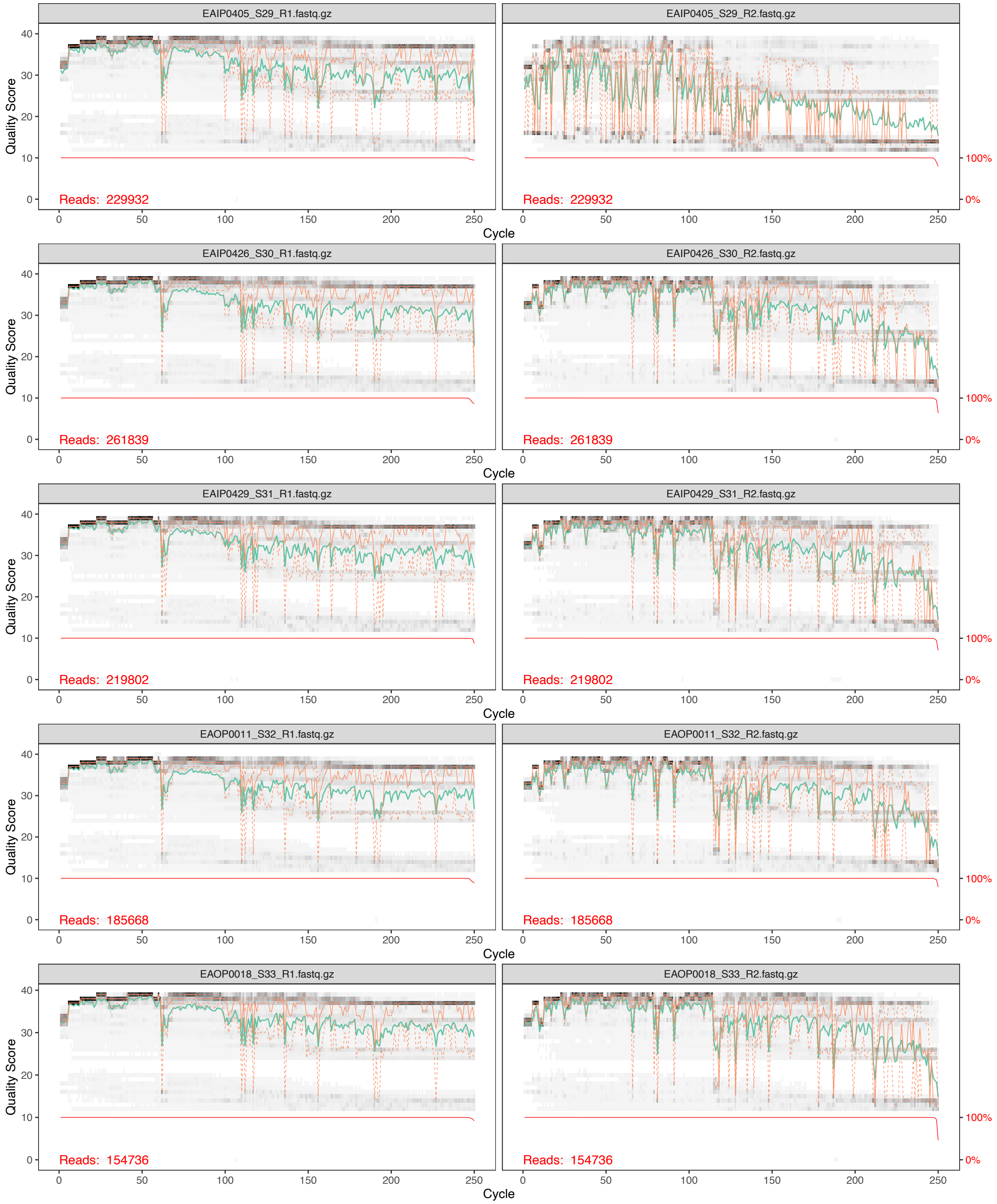
EAIP0405_S29_R1.fastq.gz
EAIP0405_S29_R2.fastq.gz
Reads: 229932
Reads: 229932
EAIP0426_S30_R1.fastq.gz
EAIP0426_S30_R2.fastq.gz
Reads: 261839
Reads: 261839
EAIP0429_S31_R1.fastq.gz
EAIP0429_S31_R2.fastq.gz
Reads: 219802
Reads: 219802
EAOP0011_S32_R1.fastq.gz
EAOP0011_S32_R2.fastq.gz
Reads: 185668
Reads: 185668
EAOP0018_S33_R1.fastq.gz
EAOP0018_S33_R2.fastq.gz
Reads: 154736
Reads: 154736
Quality Score
Cycle
100%
0%

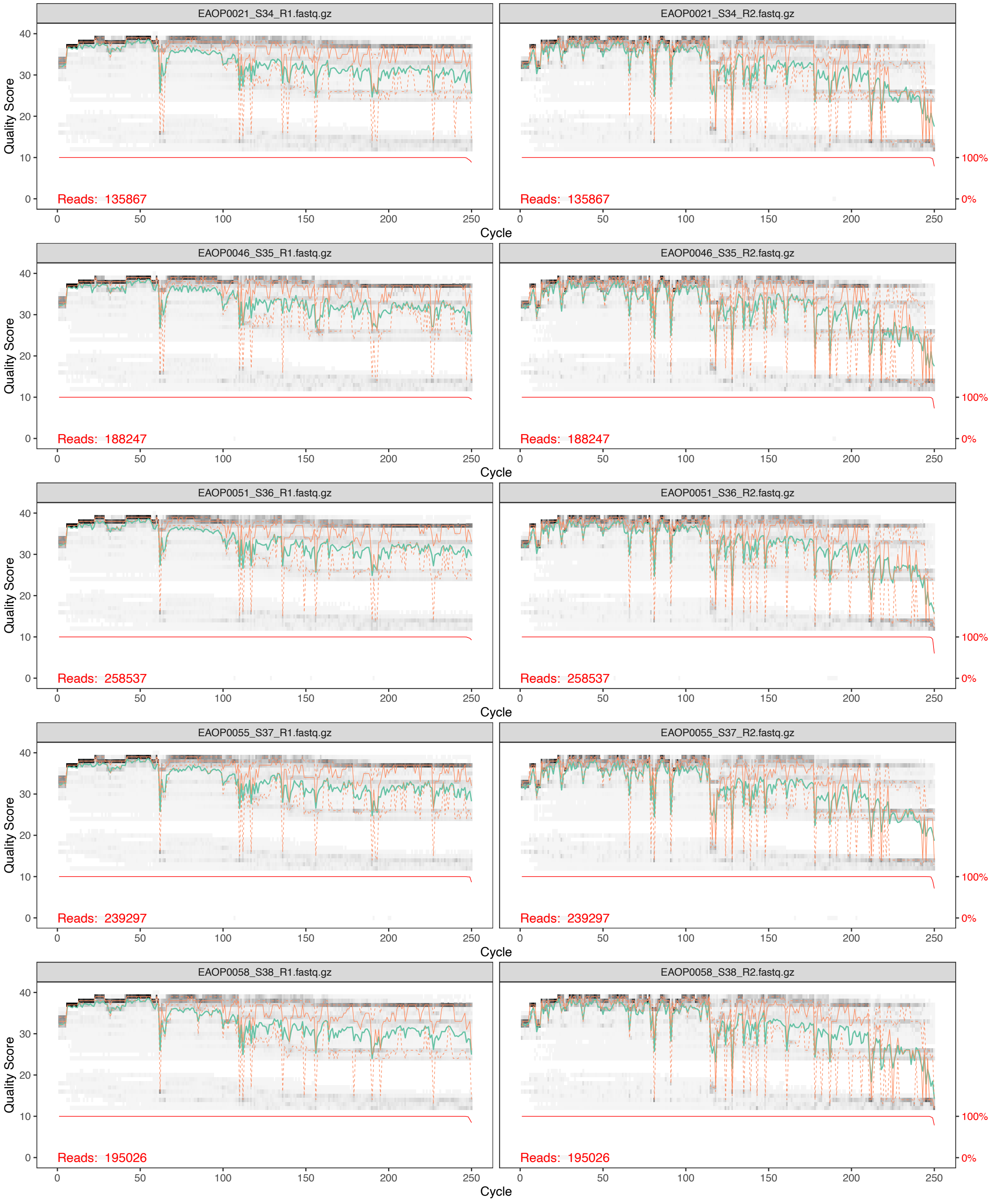
EAOP0021_S34_R1.fastq.gz
EAOP0021_S34_R2.fastq.gz
Reads: 135867
Reads: 135867
EAOP0046_S35_R1.fastq.gz
EAOP0046_S35_R2.fastq.gz
Reads: 188247
Reads: 188247
EAOP0051_S36_R1.fastq.gz
EAOP0051_S36_R2.fastq.gz
Reads: 258537
Reads: 258537
EAOP0055_S37_R1.fastq.gz
EAOP0055_S37_R2.fastq.gz
Reads: 239297
Reads: 239297
EAOP0058_S38_R1.fastq.gz
EAOP0058_S38_R2.fastq.gz
Reads: 195026
Reads: 195026
Quality Score
Cycle
0
10
20
30
40
50
100
150
200
250
100%
0%

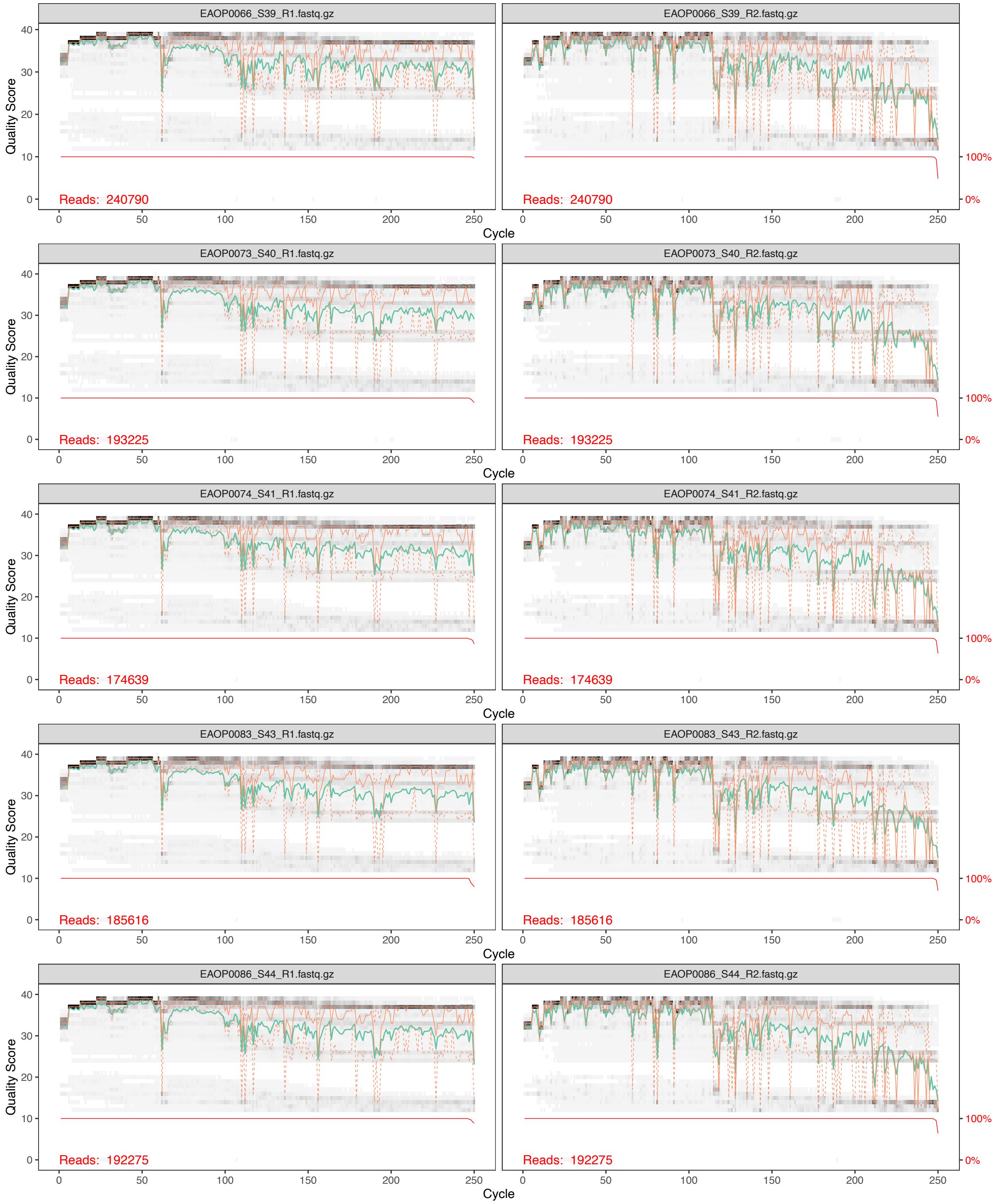
EAOP0066_S39_R1.fastq.gz
EAOP0066_S39_R2.fastq.gz
Reads: 240790
Reads: 240790
EAOP0073_S40_R1.fastq.gz
EAOP0073_S40_R2.fastq.gz
Reads: 193225
Reads: 193225
EAOP0074_S41_R1.fastq.gz
EAOP0074_S41_R2.fastq.gz
Reads: 174639
Reads: 174639
EAOP0083_S43_R1.fastq.gz
EAOP0083_S43_R2.fastq.gz
Reads: 185616
Reads: 185616
EAOP0086_S44_R1.fastq.gz
EAOP0086_S44_R2.fastq.gz
Reads: 192275
Reads: 192275
Quality Score
Cycle
100%
0%

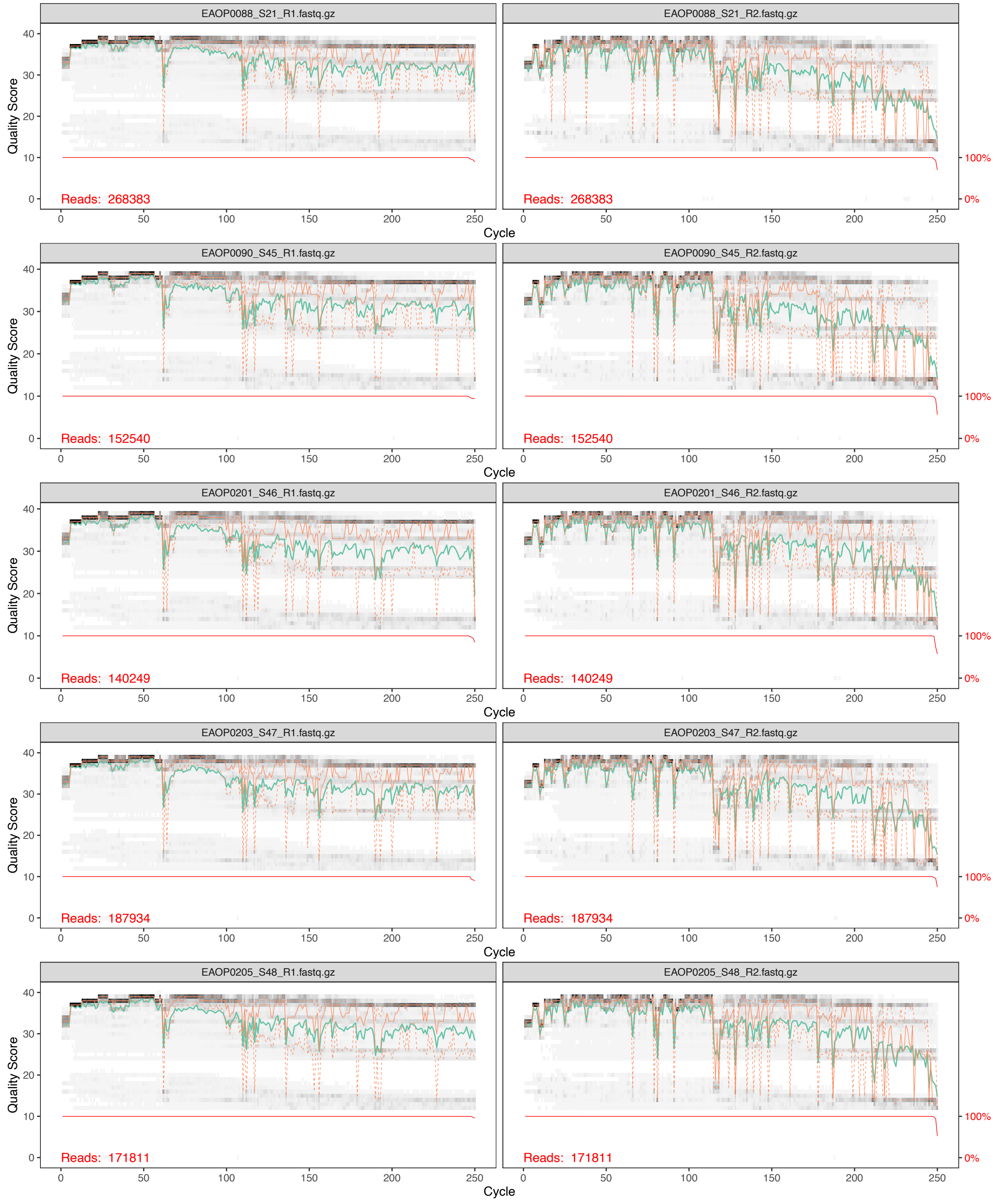
EAOP0088_S21_R1.fastq.gz
EAOP0088_S21_R2.fastq.gz
Reads: 268383
Reads: 268383
EAOP0090_S45_R1.fastq.gz
EAOP0090_S45_R2.fastq.gz
Reads: 152540
Reads: 152540
EAOP0201_S46_R1.fastq.gz
EAOP0201_S46_R2.fastq.gz
Reads: 140249
Reads: 140249
EAOP0203_S47_R1.fastq.gz
EAOP0203_S47_R2.fastq.gz
Reads: 187934
Reads: 187934
EAOP0205_S48_R1.fastq.gz
EAOP0205_S48_R2.fastq.gz
Reads: 171811
Reads: 171811
Quality Score
Cycle
100%
0%

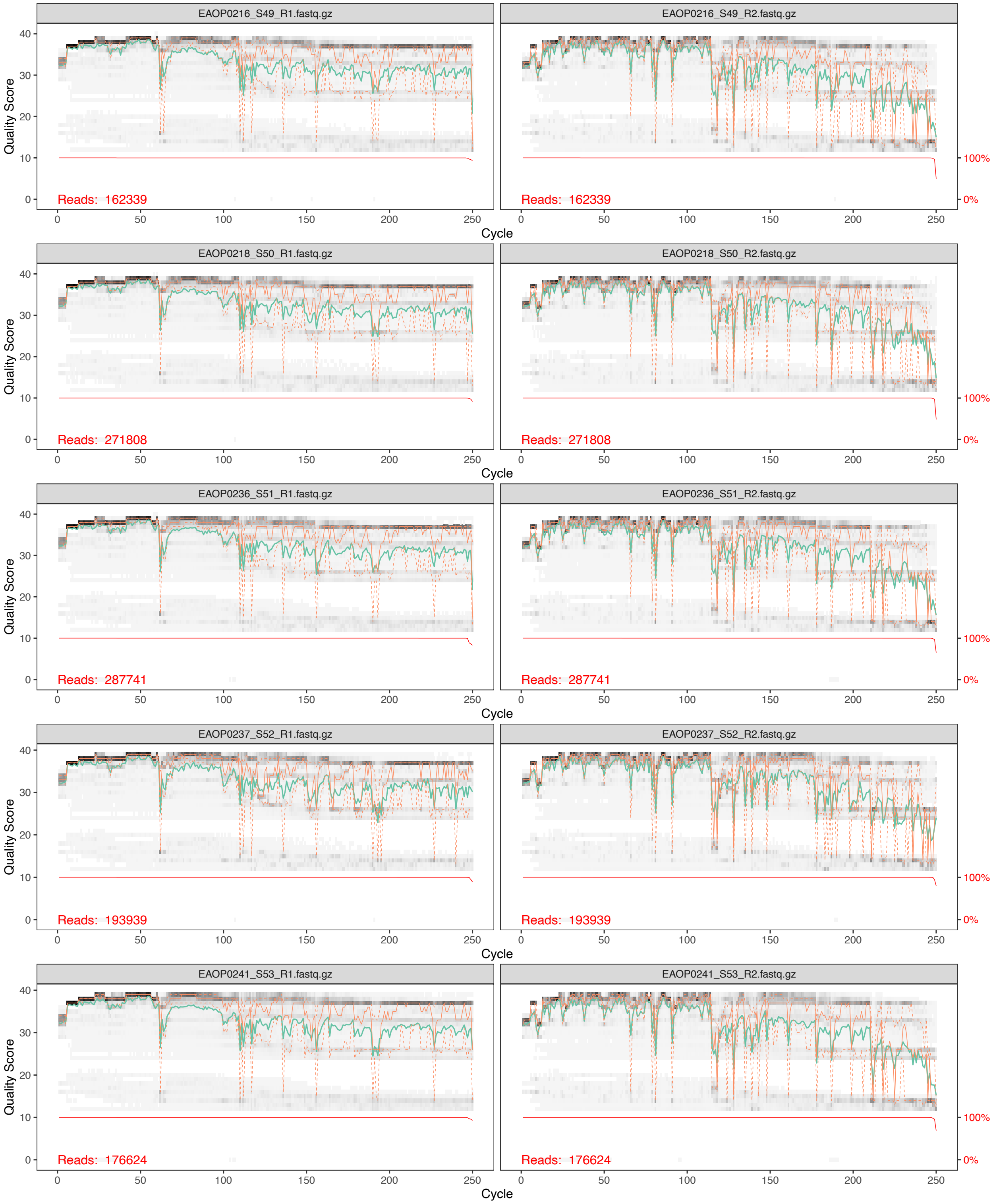
EAOP0216_S49_R1.fastq.gz
EAOP0216_S49_R2.fastq.gz
Reads: 162339
Reads: 162339
EAOP0218_S50_R1.fastq.gz
EAOP0218_S50_R2.fastq.gz
Reads: 271808
Reads: 271808
EAOP0236_S51_R1.fastq.gz
EAOP0236_S51_R2.fastq.gz
Reads: 287741
Reads: 287741
EAOP0237_S52_R1.fastq.gz
EAOP0237_S52_R2.fastq.gz
Reads: 193939
Reads: 193939
EAOP0241_S53_R1.fastq.gz
EAOP0241_S53_R2.fastq.gz
Reads: 176624
Reads: 176624
Quality Score
Cycle
100%
0%

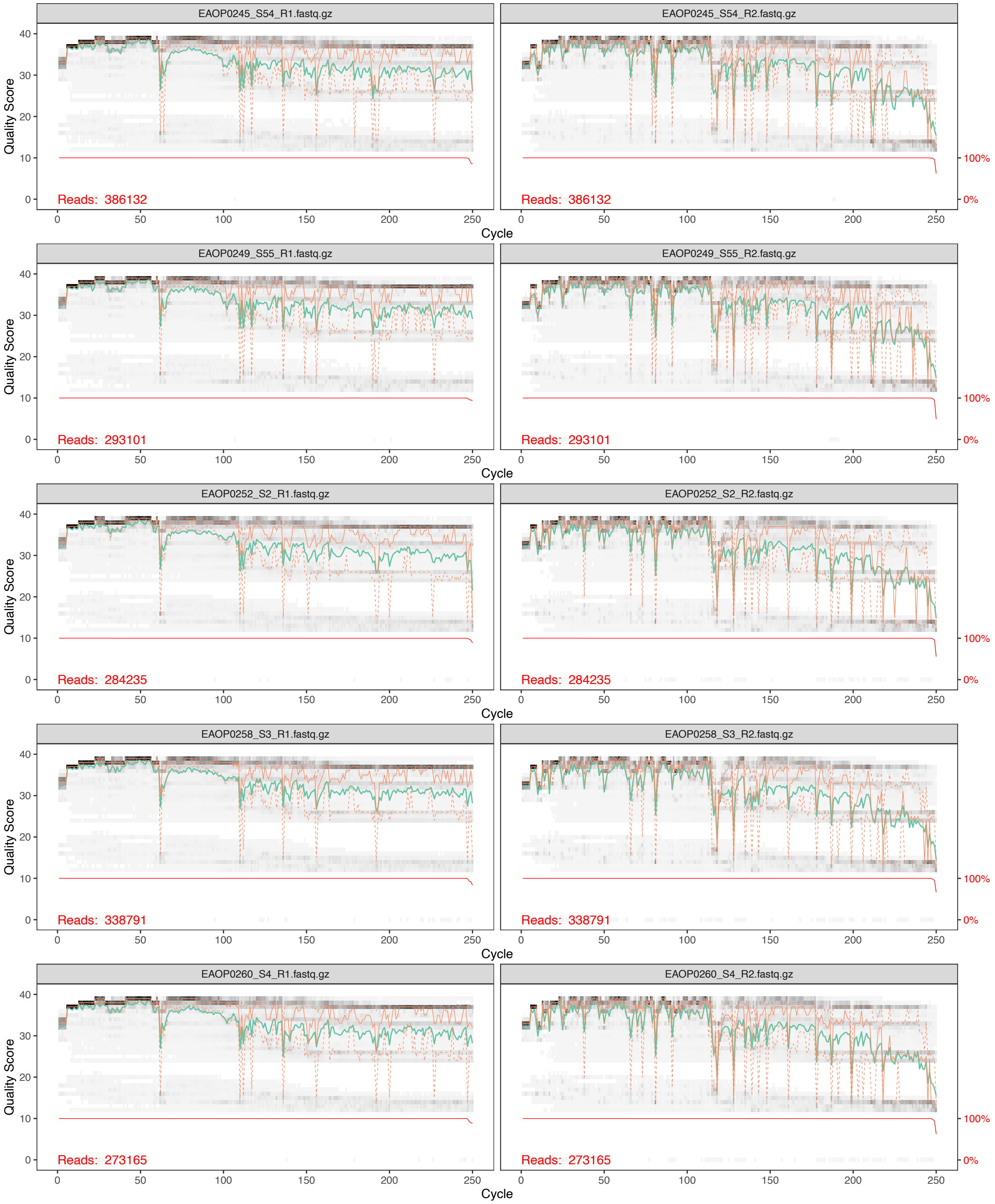
EAOP0245_S54_R1.fastq.gz
EAOP0245_S54_R2.fastq.gz
Reads: 386132
Reads: 386132
EAOP0249_S55_R1.fastq.gz
EAOP0249_S55_R2.fastq.gz
Reads: 293101
Reads: 293101
EAOP0252_S2_R1.fastq.gz
EAOP0252_S2_R2.fastq.gz
Reads: 284235
Reads: 284235
EAOP0258_S3_R1.fastq.gz
EAOP0258_S3_R2.fastq.gz
Reads: 338791
Reads: 338791
EAOP0260_S4_R1.fastq.gz
EAOP0260_S4_R2.fastq.gz
Reads: 273165
Reads: 273165
Quality Score
Cycle
100%
0%

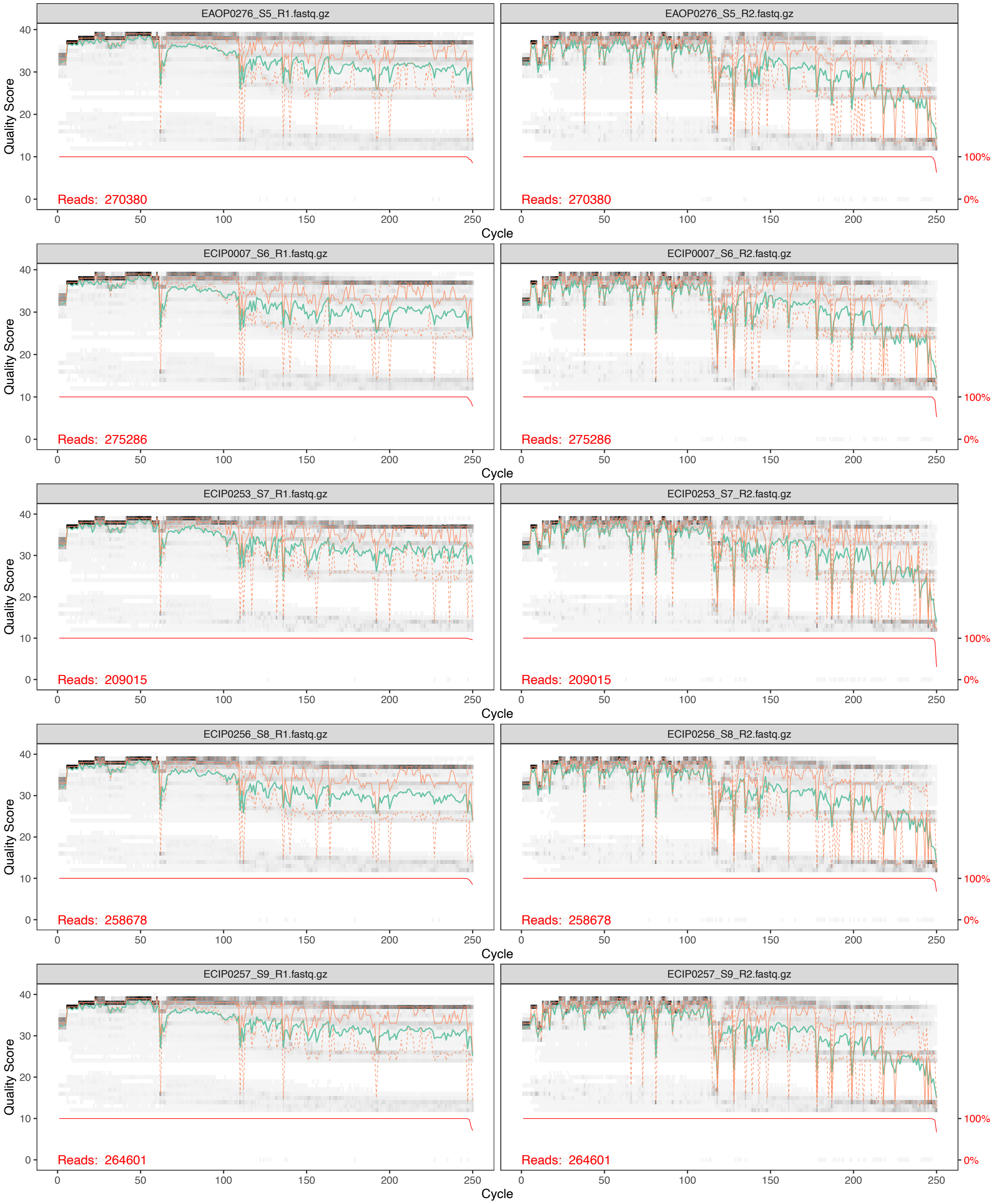

EAOP0276_S5_R1.fastq.gz
EAOP0276_S5_R2.fastq.gz
Reads: 270380
Reads: 270380
ECIP0007_S6_R1.fastq.gz
ECIP0007_S6_R2.fastq.gz
Reads: 275286
Reads: 275286
ECIP0253_S7_R1.fastq.gz
ECIP0253_S7_R2.fastq.gz
Reads: 209015
Reads: 209015
ECIP0256_S8_R1.fastq.gz
ECIP0256_S8_R2.fastq.gz
Reads: 258678
Reads: 258678
ECIP0257_S9_R1.fastq.gz
ECIP0257_S9_R2.fastq.gz
Reads: 264601
Reads: 264601
Quality Score
Cycle
0
10
20
30
40
50
100
150
200
250
100%
0%

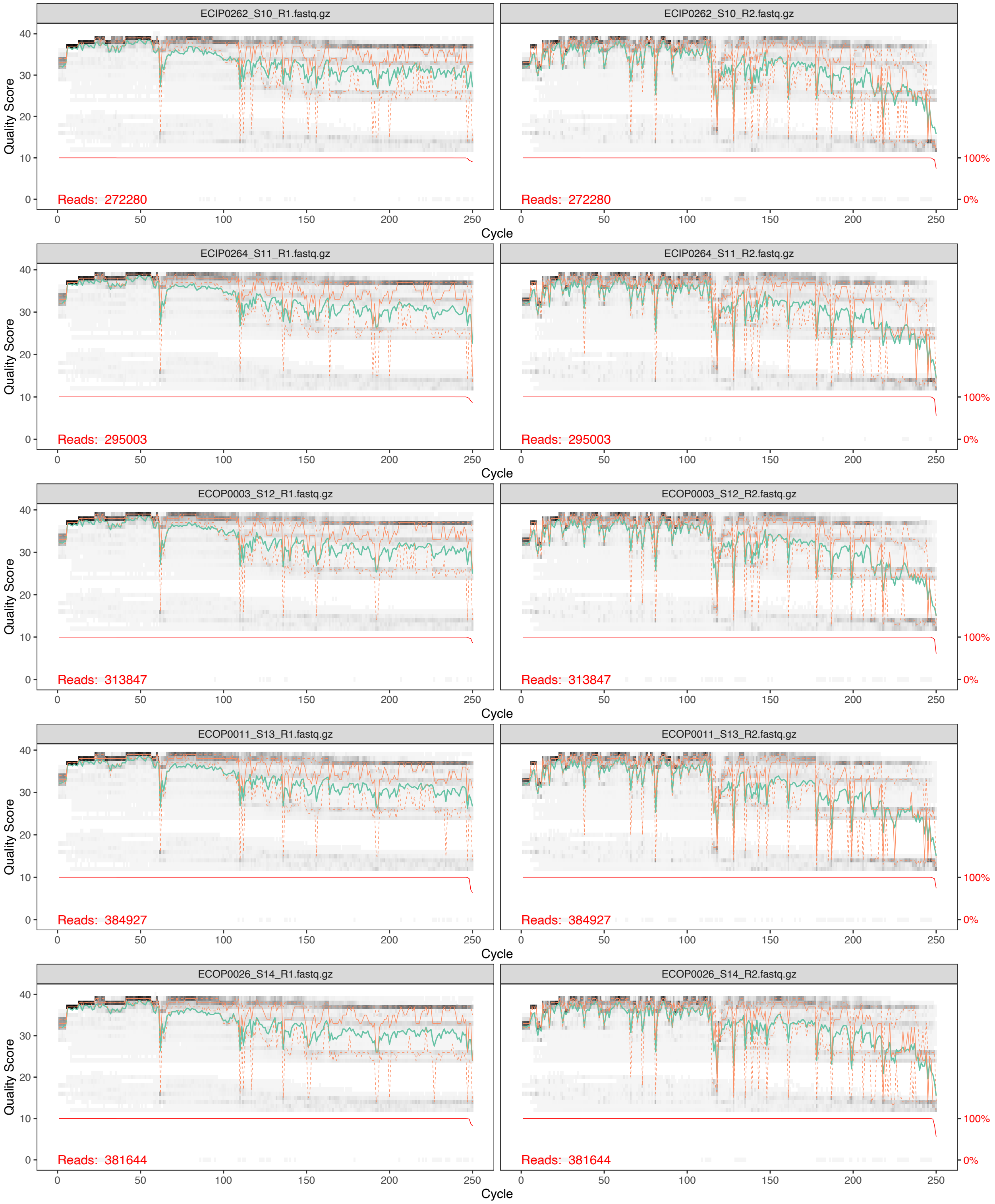
ECIP0262_S10_R1.fastq.gz
ECIP0262_S10_R2.fastq.gz
Reads: 272280
Reads: 272280
ECIP0264_S11_R1.fastq.gz
ECIP0264_S11_R2.fastq.gz
Reads: 295003
Reads: 295003
ECOP0003_S12_R1.fastq.gz
ECOP0003_S12_R2.fastq.gz
Reads: 313847
Reads: 313847
ECOP0011_S13_R1.fastq.gz
ECOP0011_S13_R2.fastq.gz
Reads: 384927
Reads: 384927
ECOP0026_S14_R1.fastq.gz
ECOP0026_S14_R2.fastq.gz
Reads: 381644
Reads: 381644
Quality Score
Cycle
100%
0%

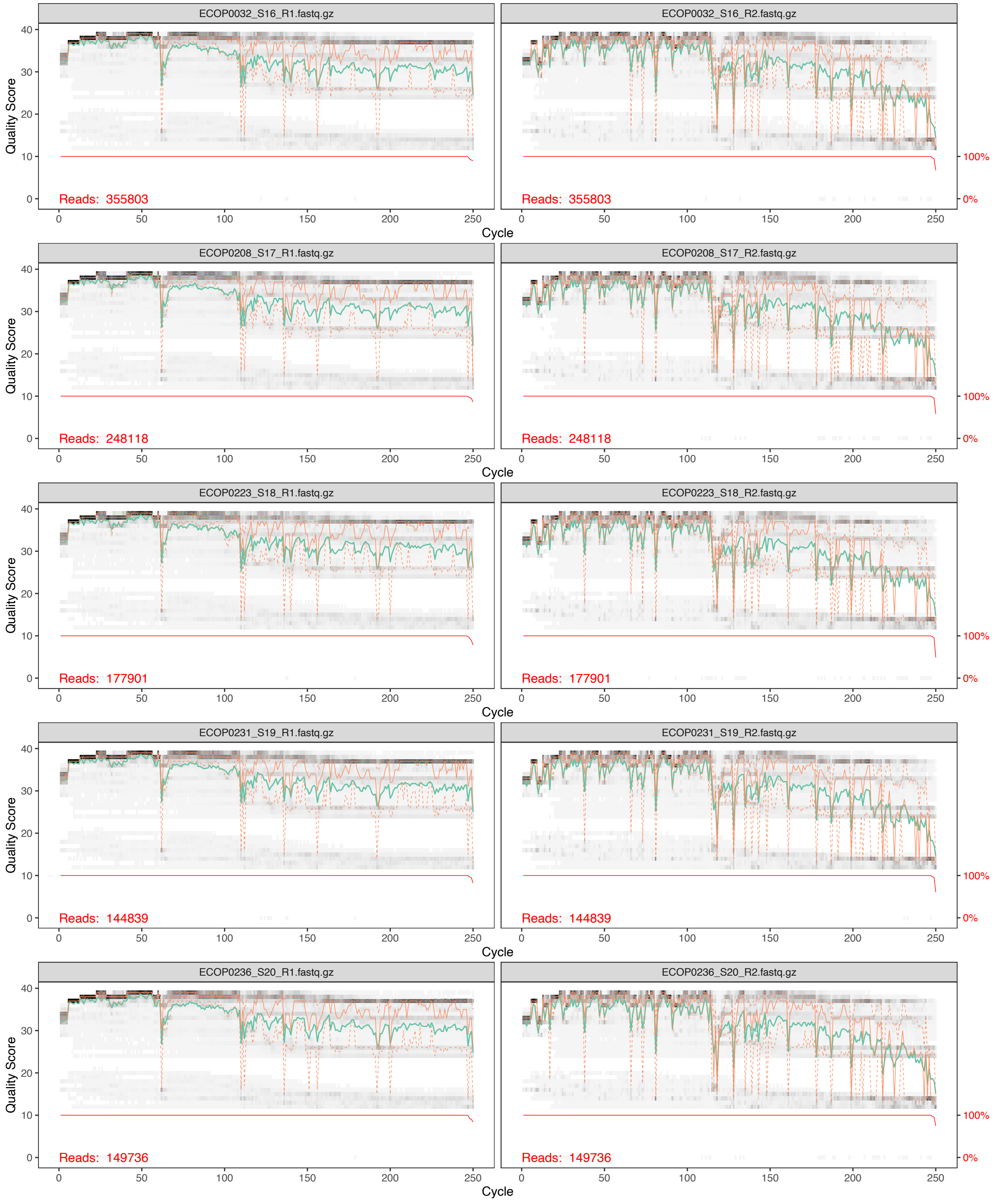
ECOP0032_S16_R1.fastq.gz
ECOP0032_S16_R2.fastq.gz
Reads: 355803
Reads: 355803
ECOP0208_S17_R1.fastq.gz
ECOP0208_S17_R2.fastq.gz
Reads: 248118
Reads: 248118
ECOP0223_S18_R1.fastq.gz
ECOP0223_S18_R2.fastq.gz
Reads: 177901
Reads: 177901
ECOP0231_S19_R1.fastq.gz
ECOP0231_S19_R2.fastq.gz
Reads: 144839
Reads: 144839
ECOP0236_S20_R1.fastq.gz
ECOP0236_S20_R2.fastq.gz
Reads: 149736
Reads: 149736
Quality Score
Cycle
0
10
20
30
40
50
100
150
200
250
100%
0%

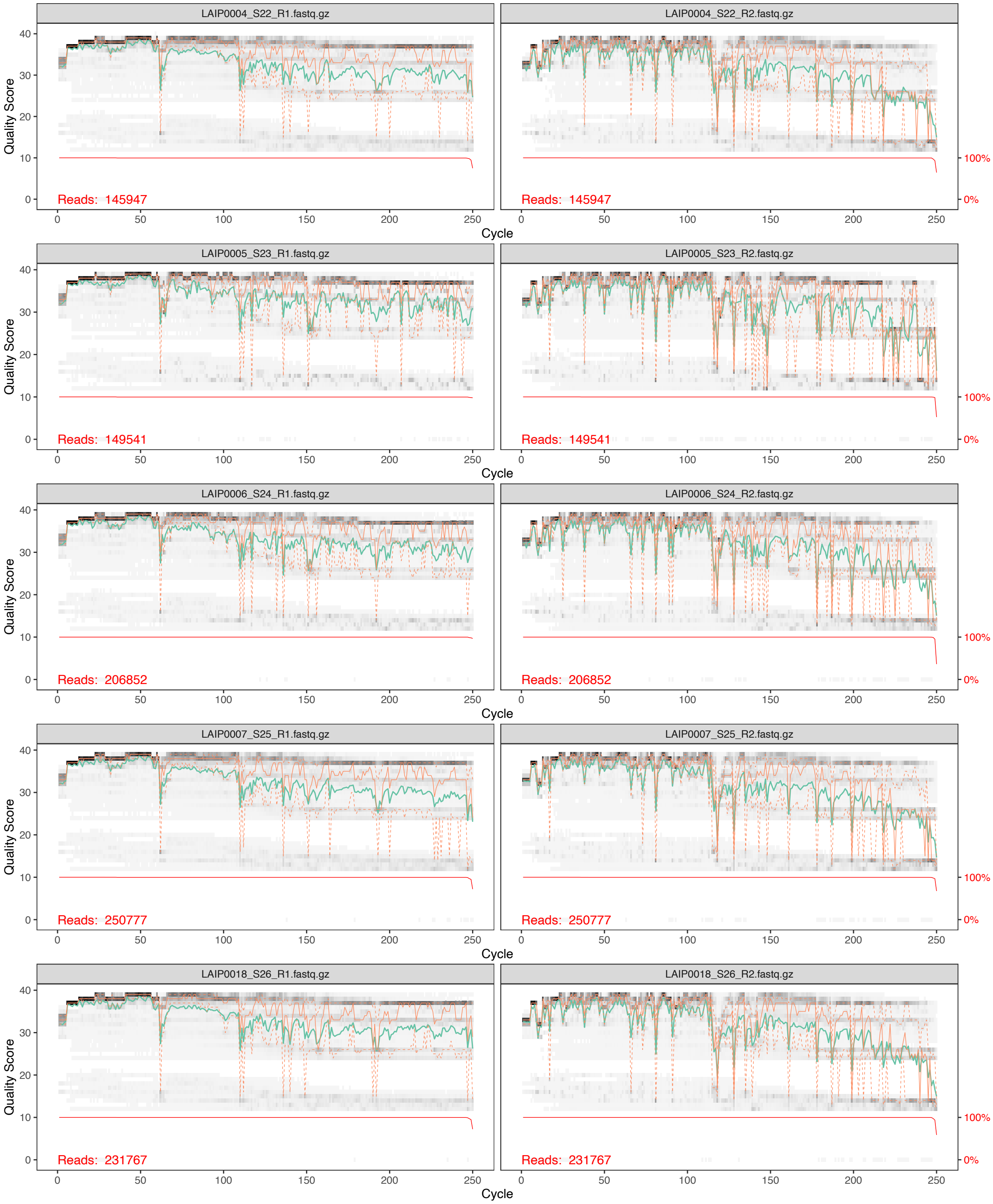
LAIP0004_S22_R1.fastq.gz
LAIP0004_S22_R2.fastq.gz
Reads: 145947
Reads: 145947
LAIP0005_S23_R1.fastq.gz
LAIP0005_S23_R2.fastq.gz
Reads: 149541
Reads: 149541
LAIP0006_S24_R1.fastq.gz
LAIP0006_S24_R2.fastq.gz
Reads: 206852
Reads: 206852
LAIP0007_S25_R1.fastq.gz
LAIP0007_S25_R2.fastq.gz
Reads: 250777
Reads: 250777
LAIP0018_S26_R1.fastq.gz
LAIP0018_S26_R2.fastq.gz
Reads: 231767
Reads: 231767
Quality Score
Cycle
100%
0%

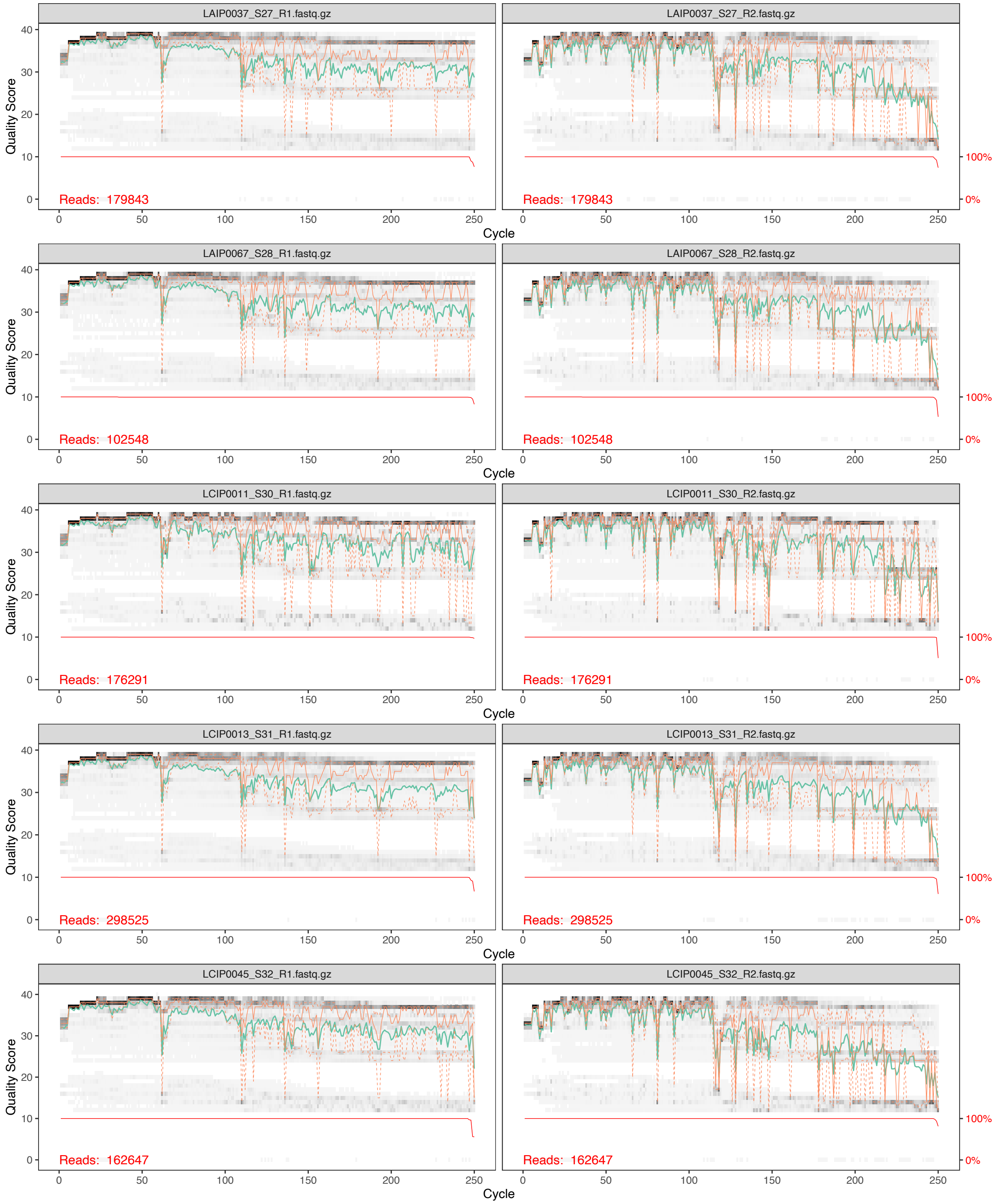
LAIP0037_S27_R1.fastq.gz
LAIP0037_S27_R2.fastq.gz
Reads: 179843
Reads: 179843
LAIP0067_S28_R1.fastq.gz
LAIP0067_S28_R2.fastq.gz
Reads: 102548
Reads: 102548
LCIP0011_S30_R1.fastq.gz
LCIP0011_S30_R2.fastq.gz
Reads: 176291
Reads: 176291
LCIP0013_S31_R1.fastq.gz
LCIP0013_S31_R2.fastq.gz
Reads: 298525
Reads: 298525
LCIP0045_S32_R1.fastq.gz
LCIP0045_S32_R2.fastq.gz
Reads: 162647
Reads: 162647
Quality Score
Cycle
100%
0%

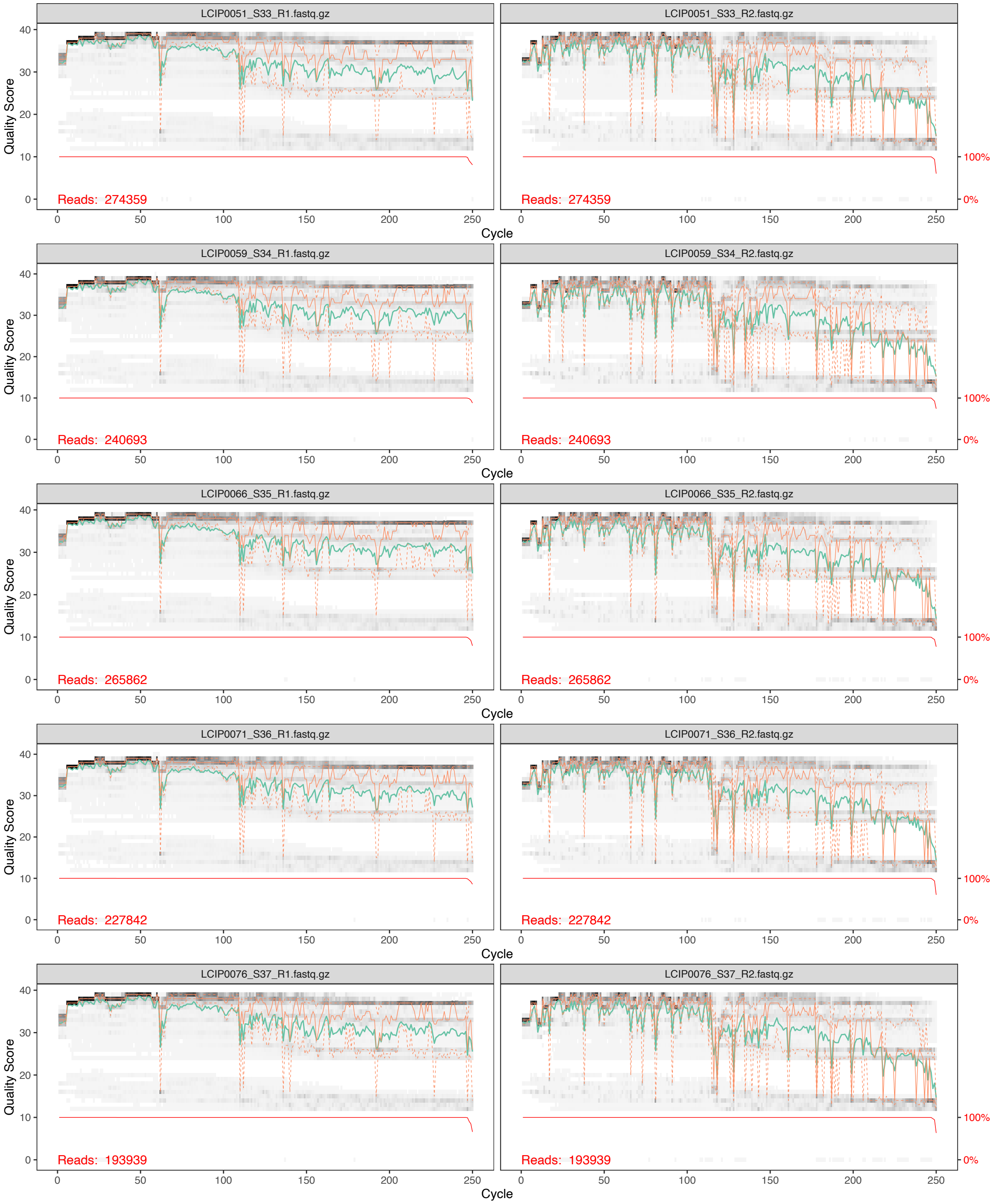
LCIP0051_S33_R1.fastq.gz
LCIP0051_S33_R2.fastq.gz
Reads: 274359
Reads: 274359
LCIP0059_S34_R1.fastq.gz
LCIP0059_S34_R2.fastq.gz
Reads: 240693
Reads: 240693
LCIP0066_S35_R1.fastq.gz
LCIP0066_S35_R2.fastq.gz
Reads: 265862
Reads: 265862
LCIP0071_S36_R1.fastq.gz
LCIP0071_S36_R2.fastq.gz
Reads: 227842
Reads: 227842
LCIP0076_S37_R1.fastq.gz
LCIP0076_S37_R2.fastq.gz
Reads: 193939
Reads: 193939
Quality Score
Cycle
100%
0%
0
10
20
30
40
50
100
150
200
250

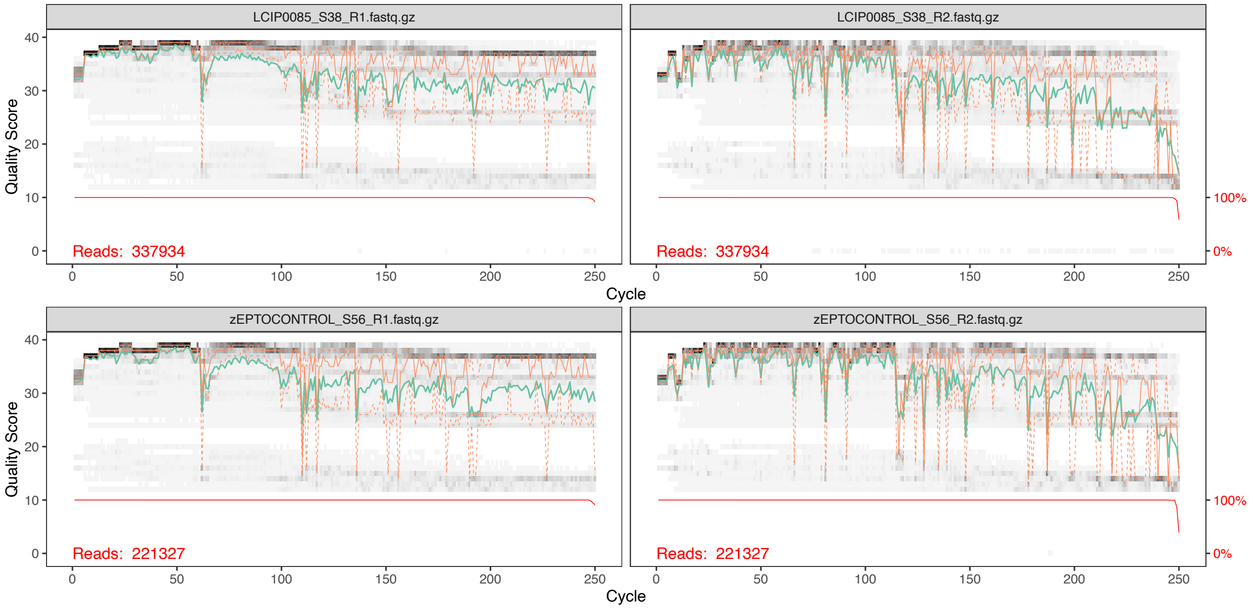
LCIP0085_S38_R1.fastq.gz
LCIP0085_S38_R2.fastq.gz
zEPTOCONTROL_S56_R1.fastq.gz
zEPTOCONTROL_S56_R2.fastq.gz
Quality Score
Cycle
Reads: 337934
Reads: 337934
Reads: 221327
Reads: 221327
100%
0%
0
10
20
30
40
50
100
150
200
250
